# Global, regional, and national epidemiology of childhood Burkitt Lymphoma from 1990 to 2021: statistical analysis of incidence, mortality, and DALYs

**DOI:** 10.3389/fpubh.2025.1560003

**Published:** 2025-07-16

**Authors:** Bing Zou, Jiezhi Jiang, Mengmei Liu, Yaxue Chen, Yan Jin, Hongjiang Pu

**Affiliations:** ^1^Department of Pediatric Surgery, Suining Central Hospital, Suining, Sichuan, China; ^2^Department of Radiology, the Third Affiliated Hospital of Kunming Medical University, Yunnan Cancer Hospital, Yunnan Cancer Centre, Kunming, China; ^3^School of Public Health, Kunming Medical University, Kunming, China; ^4^Dazhou Vocational and Technical College, Dazhou, Sichuan, China; ^5^Department of Oncology, Dazhou Central Hospital, Dazhou, Sichuan, China

**Keywords:** children, Burkitt Lymphoma, global burden of disease, disability-adjusted life years, estimated annual percentage changes

## Abstract

**Objective:**

Epidemiological data concerning Burkitt Lymphoma (BL) in children aged 0–14 years remain limited. This study examines the trends in incidence, mortality, and disability-adjusted life years (DALYs), as well as the estimated annual percentage change (EAPC) associated with childhood Burkitt Lymphoma within this demographic from 1990 to 2021.

**Methods:**

The 2021 Global Burden of Disease, Injuries, and Risk Factors Study (GBD) analytical tools were utilized to evaluate the incidence, mortality, and disability-adjusted life years (DALYs) associated with childhood Burkitt Lymphoma in individuals aged 0–14 years. This analysis incorporated variables such as age, sex, region, and sociodemographic index (SDI), utilizing data from 204 countries or regions. A comprehensive examination of health disparities was undertaken to investigate variations in mortality and DALYs among different population groups. Additionally, the Bayesian age-period-cohort (BAPC) model was employed to forecast incidence, mortality, and DALYs through the year 2035.

**Results:**

In 2021, a total of 4,083 cases of childhood Burkitt Lymphoma were documented globally. This represents an increase from 2,800 cases reported in 1990, with a 95% uncertainty interval (UI) ranging from 1,609.001 to 3,989.282, to 4,083 cases in 2021 (95% UI, 2,619.594 to 5,376.872), indicating a 31.45% rise over the period. During this 30-year span, the global incidence rate escalated from 0.172 per 100,000 individuals in 1990 (95% UI, 0.099 to 0.245) to 0.216 per 100,000 individuals in 2021 (95% UI, 0.138 to 0.285). Concurrently, the mortality rate associated with childhood Burkitt Lymphoma increased from 0.152 per 100,000 individuals in 1990 (95% UI, 0.079 to 0.224) to 0.163 per 100,000 individuals in 2021 (95% UI, 0.100 to 0.216). The highest incidence in 2021 was recorded in Sub-Saharan East Africa, while Sub-Saharan Southern Africa experienced the most pronounced increase in incidence, with an annual percent change (APC) of 3.226% (95% confidence interval [CI]: 2.092–4.373%). The age group most affected was children aged 5–9 years, who constituted 33.3% of the cases.

**Conclusion:**

Between 1990 and 2021, there was a notable rise in the incidence of childhood Burkitt Lymphoma, with a pronounced increase observed in Sub-Saharan Africa. The age group of 5–9 years exhibited the highest incidence and mortality rates, underscoring the critical need for early diagnosis and intervention. Although regions with high Socio-Demographic Index (SDI) have demonstrated advancements in reducing mortality rates, areas with low SDI necessitate improved medical resources and the implementation of standardized treatment protocols. The escalating incidence in specific regions underscores the urgent need for comprehensive research into the disease’s etiology and the development of enhanced prevention strategies.

## Introduction

Burkitt Lymphoma (BL) is recognized as one of the most prevalent forms of non-Hodgkin lymphoma in pediatric populations, characterized by its notable aggressiveness and rapid proliferation (1). This malignancy primarily affects children and adolescents, particularly in regions with high malaria prevalence, such as sub-Saharan Africa, often referred to as the “Burkitt Lymphoma Belt” (2). In Africa, Burkitt Lymphoma ranks among the most common childhood cancers, with its incidence closely associated with environmental factors, including malaria and the Epstein–Barr virus (EBV) (3). Despite these significant challenges, improved treatment protocols, similar to those implemented in Brazil, have yielded substantial therapeutic success (4). In low- and middle-income countries, resource limitations contribute to increased mortality rates among pediatric cancer patients, with persistent infections being a leading cause of death and a significant risk factor for childhood cancers (5). The management of Burkitt Lymphoma in these regions is hindered by inadequate diagnostic capabilities, resulting in reduced cure rates. Nevertheless, interventions such as public health awareness campaigns and telepathology offer scalable and cost-effective solutions that have the potential to substantially improve outcomes (6). Advances in molecular biology have enhanced our understanding of the molecular mechanisms underlying Burkitt Lymphoma, revealing novel therapeutic targets and paving the way for innovative treatments, including targeted therapy and immunotherapy, thereby providing renewed hope for better patient prognoses. For example, the aberrant expression of proteins such as EZH2, BET, and PRMT is closely associated with the pathogenesis of Burkitt Lymphoma, and clinical trials targeting these proteins have demonstrated promising therapeutic efficacy (7). Moreover, immunotherapy is increasingly recognized as a promising therapeutic approach, with immune checkpoint inhibitors such as nivolumab demonstrating favorable outcomes in the treatment of various B-cell lymphomas (8). Empirical research has revealed significant variations in the incidence of Burkitt Lymphoma across different age groups and geographical regions. For example, studies conducted across four continents have identified incidence peaks at approximately ages 10, 40, and 70, indicating potential distinct etiological or biological factors at different life stages (9). The Global Burden of Disease (GBD) study highlights that incidence rates vary by location and age, and systematic analyses can enhance our understanding of these epidemiological patterns. This understanding is crucial for the development of targeted public health policies and interventions. Such measures include improving early diagnostic capabilities, refining treatment protocols, and developing vaccines, all aimed at reducing global incidence and mortality rates (10). In sub-Saharan Africa, Burkitt Lymphoma is often linked to HIV infection, and the implementation of traditional high-intensity treatments is frequently unfeasible in resource-constrained environments. This situation necessitates the formulation of adaptable treatment protocols. Current research suggests that combined therapeutic approaches, incorporating anthracyclines and/or high-dose methotrexate, are demonstrating potential efficacy. This highlights the urgent need for international collaboration and innovation to address the disparity in treatment effectiveness between low- and high-income countries (11). Analyzing regional incidence, mortality, and disease burden is essential for identifying high-risk populations and areas, thereby providing data-driven insights for public health policy. For example, a study conducted in England, utilizing data from 1990 to 2013, revealed ongoing health improvements alongside persistent inequalities. This underscores the necessity for systemic interventions aimed at risk mitigation, promotion of healthy behaviors, and reduction of chronic disease severity (12). This study aims to utilize the Global Burden of Disease (GBD) database to analyze global, regional, and national trends in the incidence, mortality, and disability-adjusted life years (DALYs) associated with childhood Burkitt Lymphoma from 1990 to 2021. Through a systematic examination of extensive data, this research seeks to identify changes in the disease burden of childhood Burkitt Lymphoma, providing scientific evidence to guide global health policymakers in optimizing resource allocation and improving disease prevention and treatment strategies.

## Methods

### Data sources and analytical framework

The Global Burden of Disease (GBD) database, led by the Institute for Health Metrics and Evaluation (IHME) at the University of Washington, serves as a leading repository for global epidemiological research. Its primary aim is to rigorously quantify health detriments attributable to a wide range of diseases, injuries, and risk factors. The GBD initiative represents disease burden through three key indicators: mortality, incidence, and disability-adjusted life years (DALYs). DALYs integrate years of life lost (YLL) due to premature death and years lived with disability (YLD), calculated as follows: DALYs = YLL + YLD. The YLL metric is determined by multiplying the number of deaths by the standard life expectancy, while YLD is calculated based on disease prevalence and associated disability weights (13). Disability weights, determined by expert consensus, range from 0 (indicating perfect health) to 1 (representing death). In this study, we employed the Global Burden of Disease (GBD) database to investigate the incidence, mortality rates, and disability-adjusted life years (DALYs) associated with childhood Burkitt Lymphoma among individuals aged 0 to 14 years, spanning the period from 1990 to 2021. Data were retrieved from the GBD database^1^ on December 1, 2024, encompassing 204 countries and regions. The analysis was stratified by sex, age groups (<1 year, 1–2 years, 2–4 years, 5–9 years, and 10–14 years), and geographic location. Due to the absence of racial or ethnic data in the GBD database, such analyses were not conducted. This cross-sectional study, which did not involve any personally identifiable information, was granted ethical approval by the Ethics Committee of Kunming Medical University, which waived the need for informed consent, in full compliance with the guidelines established by the Strengthening the Reporting of Observational Studies in Epidemiology (STROBE) statement (14).

### Sociodemographic index (SDI)

The Socio-Demographic Index (SDI) functions as an integrative measure of a nation or region’s socioeconomic development, encompassing dimensions such as economic structure, educational attainment, living standards, and social welfare. The SDI is quantified on a scale ranging from 0 to 1, with higher values indicating more advanced socioeconomic progress. Within the Global Burden of Disease (GBD) framework, countries and regions are categorized into five distinct tiers based on their SDI: low, low-middle, middle, high-middle, and high. This categorization enables a detailed analysis of the impact of socioeconomic and geographical disparities on the incidence and burden of childhood Burkitt Lymphoma.

### Statistical analysis

Using the GBD database, we calculated incidence rates, mortality rates, and Disability-Adjusted Life Years (DALYs) per 100,000 population, along with their corresponding 95% Uncertainty Intervals (UIs). The Joinpoint regression model was utilized to determine the Annual Percentage Change (APC) and its 95% Confidence Interval (CI) for trend analysis across different temporal periods (15). Additionally, a log-linear regression model was employed to estimate the Average Annual Percent Change (EAPC) and its CI, providing insights into long-term trends in childhood Burkitt Lymphoma from 1990 to 2021. An EAPC value with a 95% CI lower bound greater than zero indicates an upward trend, whereas a lower bound less than zero indicates a downward trend. Furthermore, we investigated the correlation between disease burden indicators and the SDI through curve fitting. All statistical analyses were conducted using R version 4.3.3, with statistical significance set at *p* < 0.05.

### Bayesian age-period-cohort (BAPC) modeling and projections

Future disease burden was projected using the Bayesian Age-Period-Cohort (BAPC) model, implemented through the “BAPC” R package. This model integrates GBD 2021 data and IHME population forecasts, accounting for age, period, and cohort effects using integrated nested Laplace approximations (INLA).

### Cross-national inequality assessment

Inequalities in the burden of Childhood Burkitt Lymphoma across countries were assessed using the SII and concentration index. The SII was derived by regressing disability rates against the cumulative population distribution ranked by SDI. Data covering 204 countries and regions from 1990 to 2021 were analyzed using robust linear models (RLM) to mitigate the impact of outliers. The concentration index was calculated based on the Lorenz curve of DALYs against the cumulative population distribution ranked by SDI.

### Ethical statement

This study was approved by the Ethics Committee of Kunming Medical University with a waiver for informed consent.

### Funding

The funding entities had no involvement in the conceptualization, data collation, analysis, interpretation, or manuscript drafting. All authors had unfettered access to the study data and assume full responsibility for the decision to submit the manuscript for publication.

## Results

### Global burden of childhood Burkitt Lymphoma: a trend analysis

#### Incidence

**Figure 1:**
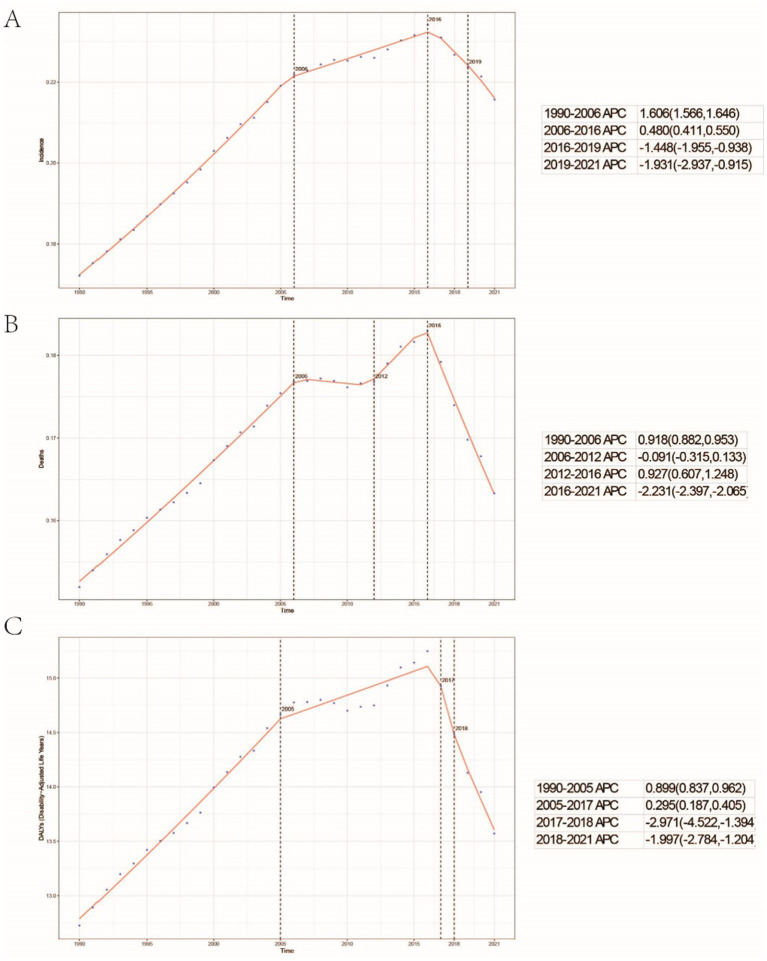
Global trends in the incidence rate **(A)**, mortality rate **(B)**, and disability-adjusted life years (DALYs) rate **(C)** of childhood Burkitt lymphoma from 1990 to 2021.

**Figure 2:**
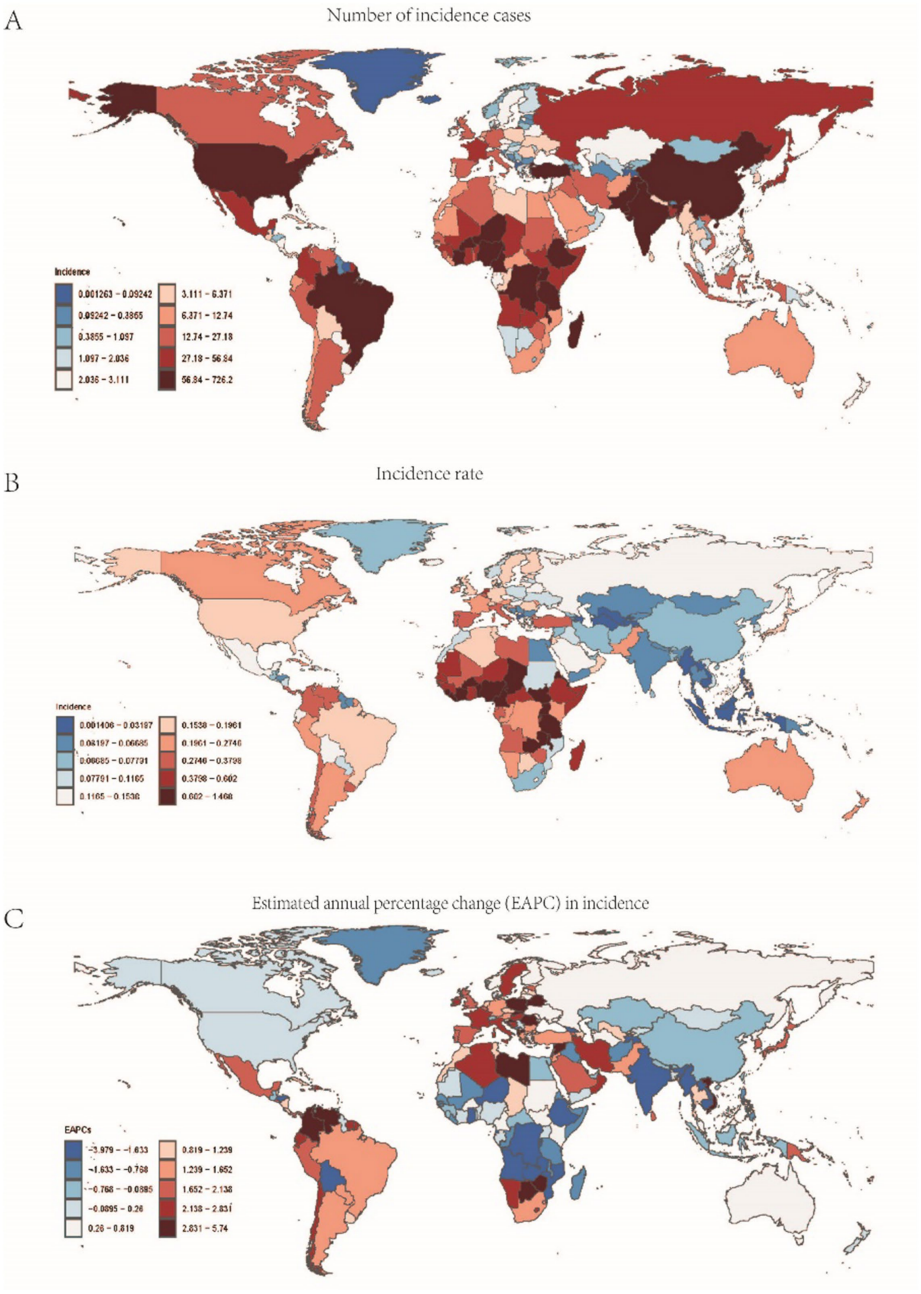
Data related to the incidence of childhood Burkitt Lymphoma: **(A)** Number of incidence cases; **(B)** Incidence rate; **(C)** Annual percentage change (EAPC) in incidence rate.

**Figure 3:**
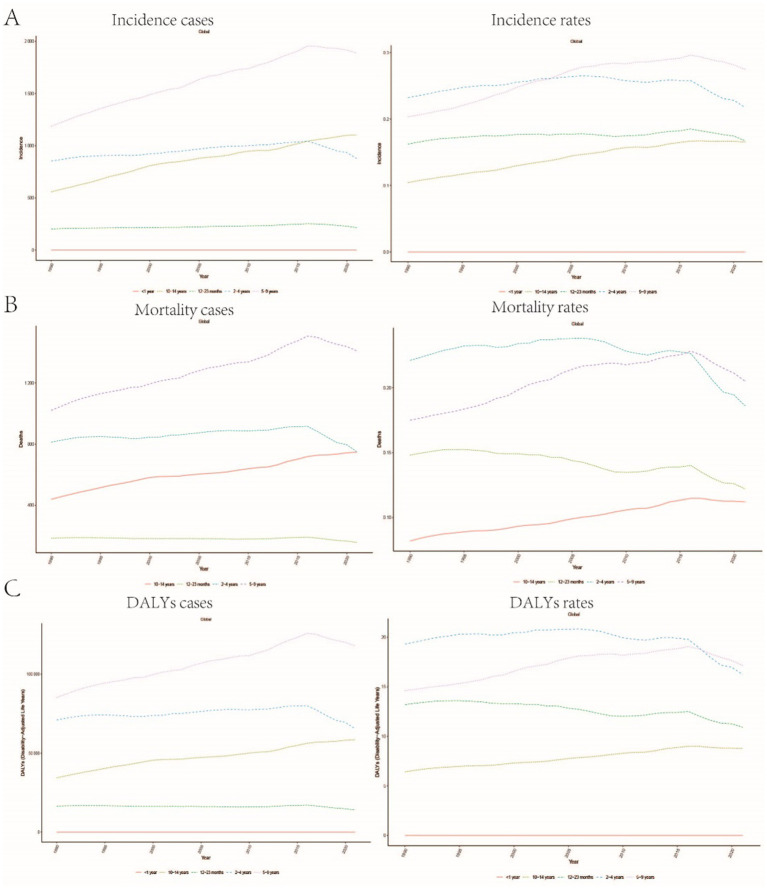
Trends in age-stratified incidence, mortality, and DALYs rates for childhood Burkitt Lymphoma: **(A)** Incidence rate trends over time for different age groups (<1 year, 12–23 months, 2–4 years, 5–9 years, 10–14 years). **(B)** Age-stratified mortality rate trends. **(C)** Changes in DALYs rate across different age groups.

**Figure 4:**
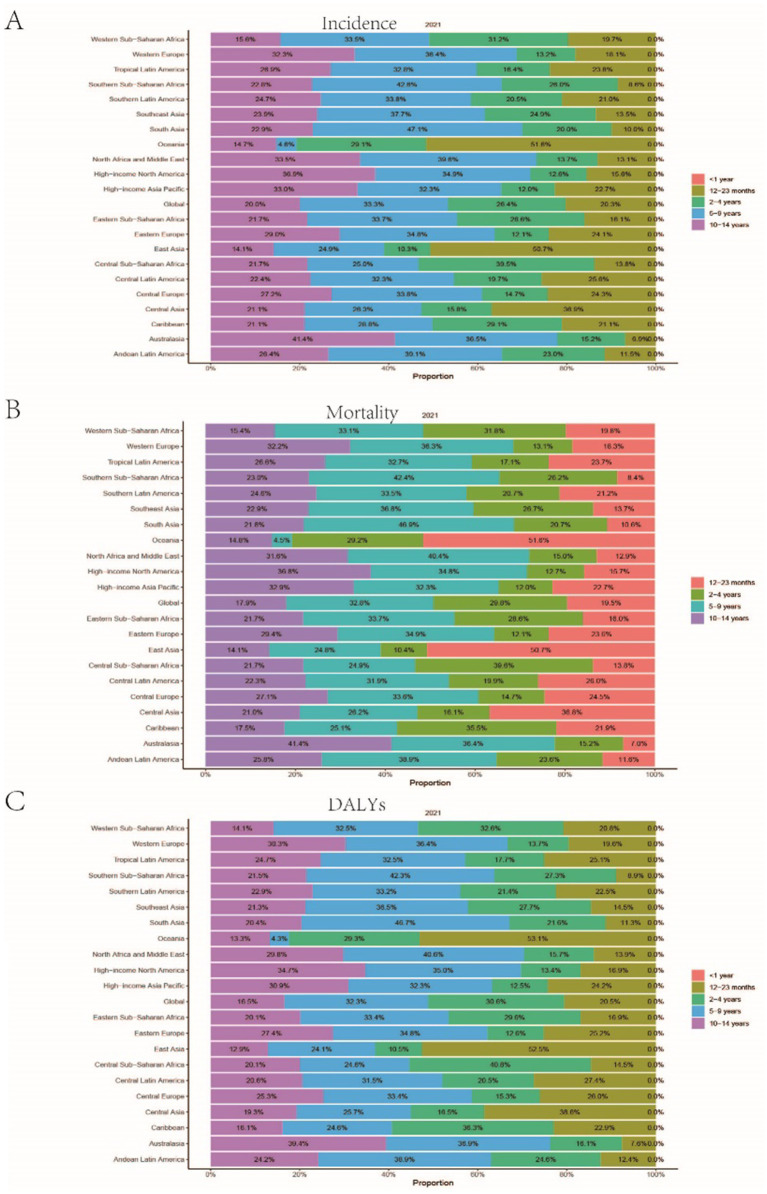
Presents the age distribution ratio of childhood Burkitt Lymphoma across various global regions in 2021. **(A–C)** respectively, indicate the distribution proportion of incidence rate, mortality rate, and Disability-Adjusted Life Years (DALYs) for different age groups across these regions.

A comprehensive analysis of data sourced from the Global Burden of Disease (GBD) database indicates significant variability in the global incidence trends of Burkitt Lymphoma. Initially, there is an increase in incidence, followed by a subsequent decline. From 1990 to 2006, the annual percentage change (APC) reached its peak at 1.606% (95% Uncertainty Interval [UI], 1.566 to 1.646%) (Figure 1A), with the highest incidence observed in 2016 (Figure 1A). Globally, the incidence of Burkitt Lymphoma rose from 2,800 cases (95% UI, 1,609.001 to 3,989.282) in 1990 to 4,083 cases (95% UI, 2,619.594 to 5,376.872) in 2021, representing a total increase of 31.45%. Similarly, the incidence rate increased from 0.172 per 100,000 individuals (95% UI, 0.099 to 0.245) in 1990 to 0.216 per 100,000 individuals (95% UI, 0.138 to 0.285) in 2021. The estimated annual percentage change (EAPC) was 0.883% (95% Confidence Interval [CI], 0.064 to 1.709) (Table 1 and Figure 2). A significant increase in incidence rates was observed among children aged 5–9 years, identifying them as the cohort with the highest incidence rate, comprising 33.3% of the cases. Although there was an increase in the incidence rate among children aged 10–14 years, it remained the second-lowest, while the incidence rates declined for both the 2–4 and 12–23 age groups. The incidence rate for infants under 1 year of age remained stable (Figures 3A, 4A). Across genders, males generally exhibited higher incidence rates compared to females, with the exception of the 12–23 age group, where females predominated. This gender disparity was particularly pronounced in children aged 5–9 years (Supplementary Figure S1A).

**Table 1:** The number of incident cases and incidence rates of Burkitt Lymphoma in children globally, in 5 SDI regions, and in 21 regions in 1990 and 2021, as well as the annual percentage change (EAPC) from 1990 to 2021.

Location	Rate per 100,000 (95% UI)				
	1990		2021		1990–2021
	Incident cases	Incident rate	Incident cases	Incident rate	EAPC
Global	2799.735(1609.001,3989.282)	0.172(0.099,0.245)	4083.401(2619.594,5376.872)	0.216(0.138,0.285)	0.883(0.064,1.709)
Regions					
East Asia	166.364(69.578,287.070)	0.054(0.023,0.094)	177.785(91.641,324.389)	0.071(0.036,0.129)	−0.479(−1.244,0.291)
Southeast Asia	40.636(12.862,80.118)	0.025(0.008,0.050)	60.302(26.515,110.549)	0.037(0.016,0.068)	0.493(−0.557,1.554)
Oceania	0.580(0.192,1.671)	0.023(0.008,0.065)	2.042(0.529,5.606)	0.042(0.011,0.115)	2.100(1.798,2.403)
Central Asia	7.889(4.234,14.411)	0.034(0.018,0.061)	6.858(3.797,11.406)	0.027(0.015,0.044)	−1.149(−2.136,-0.151)
Central Europe	16.618(9.694,30.594)	0.059(0.035,0.110)	20.355(8.954,30.877)	0.121(0.053,0.183)	2.642(1.430,3.868)
Eastern Europe	59.789(32.670,95.309)	0.122(0.067,0.195)	40.515(16.296,60.675)	0.116(0.046,0.175)	0.837(−0.048,1.729)
High-income Asia Pacific	30.213(13.731,51.219)	0.088(0.040,0.150)	36.291(16.012,54.919)	0.164(0.072,0.251)	2.064(0.886,3.255)
Australasia	7.759(4.588,12.459)	0.179(0.106,0.288)	11.344(5.230,19.075)	0.204(0.094,0.344)	0.440(−0.707,1.600)
Western Europe	98.656(63.985,160.406)	0.146(0.095,0.238)	161.096(66.104,257.943)	0.245(0.101,0.394)	1.907(0.780,3.046)
Southern Latin America	22.632(13.883,35.761)	0.162(0.099,0.256)	35.609(21.045,53.167)	0.255(0.150,0.382)	1.824(0.930,2.726)
High-income North America	122.568(81.129,175.597)	0.213(0.141,0.305)	121.893(74.409,174.263)	0.192(0.116,0.274)	0.206(−1.136,1.566)
Caribbean	23.255(12.173,47.157)	0.219(0.115,0.443)	19.222(9.103,38.506)	0.179(0.084,0.360)	0.446(−0.205,1.102)
Andean Latin America	17.918(9.229,32.068)	0.130(0.067,0.232)	31.875(15.396,58.457)	0.188(0.091,0.344)	1.268(0.427,2.116)
Central Latin America	56.511(37.102,91.117)	0.095(0.062,0.152)	100.813(55.781,144.896)	0.168(0.093,0.242)	2.151(1.420,2.888)
Tropical Latin America	64.098(42.349,98.775)	0.127(0.084,0.196)	84.094(42.820,121.817)	0.179(0.091,0.259)	1.367(0.571,2.169)
North Africa and Middle East	125.323(63.615,227.998)	0.096(0.049,0.175)	206.484(125.841,316.830)	0.119(0.072,0.182)	0.864(−0.161,1.899)
South Asia	274.789(93.253,527.885)	0.067(0.023,0.129)	332.691(174.329,555.892)	0.069(0.036,0.117)	−0.018(−0.489,0.456)
Central Sub-Saharan Africa	132.278(34.349,245.947)	0.541(0.147,0.996)	140.602(63.142,228.999)	0.256(0.115,0.417)	−2.044(−3.334,-0.738)
Eastern Sub-Saharan Africa	876.591(364.690,1374.708)	1.035(0.434,1.616)	1136.858(642.112,1662.022)	0.684(0.387,0.999)	−1.261(−1.990,-0.527)
Southern Sub-Saharan Africa	12.362(6.150,21.375)	0.064(0.032,0.111)	32.087(15.069,55.916)	0.142(0.066,0.247)	3.226(2.092,4.373)
Western Sub-Saharan Africa	642.907(274.460,998.166)	0.773(0.337,1.189)	1324.585(700.382,1889.375)	0.661(0.352,0.940)	−0.334(−1.237,0.578)
SDI					
High-middle SDI	261.536(154.877,396.973)	0.102(0.060,0.155)	322.621(193.019,449.933)	0.145(0.086,0.203)	1.231(0.321,2.150)
High SDI	245.040(170.416,363.019)	0.139(0.097,0.206)	306.274(153.051,435.605)	0.184(0.091,0.262)	1.233(0.029,2.452)
Low-middle SDI	567.860(288.284,864.689)	0.128(0.065,0.195)	874.385(573.772,1262.660)	0.161(0.105,0.233)	0.768(0.039,1.502)
Low SDI	1373.091(599.575,2180.712)	0.635(0.280,1.002)	2021.868(1144.070,2803.392)	0.472(0.268,0.653)	−0.836(−1.637,-0.028)
Middle SDI	350.256(209.942,495.827)	0.065(0.039,0.092)	555.788(317.643,778.255)	0.103(0.059,0.145)	1.494(0.729,2.265)

#### Mortality

The trends in mortality closely paralleled those observed in incidence, with mortality related to Burkitt Lymphoma initially experiencing an increase before declining over the past three decades. Between 2012 and 2016, the APC reached a peak of 0.927% (95% CI, 0.607 to 1.248%), with mortality rates reaching their highest point in 2016 (Figure 1B). In 1990, there were 2,457 deaths (95% UI, 1,278.712 to 3,631.749) globally attributable to Burkitt Lymphoma, which increased to 3,065 deaths (95% UI, 1,880.691 to 4,046.269) in 2021, representing an overall increase of 19.84% (Supplementary Table S1 and Supplementary Figure S2). Similarly, the mortality rate rose from 0.152 per 100,000 individuals (95% UI, 0.079 to 0.224) in 1990 to 0.163 per 100,000 individuals (95% UI, 0.100 to 0.216) in 2021, indicating a 6.75% increase. The EAPC was 0.402% (95% CI, −0.375 to 1.185) (Supplementary Table S1). Consistent with incidence trends, mortality rates increased in the 5–9 and 10-14-year age groups while decreasing in the 2–4 and 12-23-year age groups. By 2021, the highest mortality rate was recorded in children aged 5–9 years, followed by the 2–4, 12–23, and 10-14-year age groups (Figures 3B, 4B). In terms of gender differences, boys aged 2 to 14 years exhibited higher mortality rates compared to girls, particularly in the 5 to 9 years age group. In contrast, girls aged 12 to 23 years demonstrated slightly higher mortality rates than boys, as illustrated in Supplementary Figure S1B.

### Disability-adjusted life years (DALYs)

Associated with Burkitt Lymphoma mirrored the patterns observed in both incidence and mortality, characterized by an initial increase followed by a subsequent decrease over the past three decades. Between 1990 and 2005, the APC reached its maximum at 0.899% (95% CI, 0.837 to 0.962%) (Figure 1C), with DALYs peaking in 2016 (Figure 1C). In 1990, the global burden of Burkitt Lymphoma, measured in DALYs, was 207,119.995 (95% UI, 107,272.072 to 306,869.858), which increased to 255,842.921 (95% UI, 156,206.467 to 338,269.710) by 2021, representing an overall growth of 19.04% (Supplementary Table S2). The rate of DALYs increased from 12.725 per 100,000 individuals (95% UI, 6.604 to 18.836) in 1990 to 13.571 per 100,000 individuals (95% UI, 8.236 to 17.994) in 2021, reflecting a 6.23% increase. The EAPC was 0.375% (95% CCI, −0.402 to 1.158%) (refer to Supplementary Table S2). Notably, there was a significant rise in the number of DALYs among the 5–9 and 10–14-year age groups, with the most pronounced increase observed in the 5–9 age cohort. In contrast, a decline was noted in the 2–4 and 12–23-year age groups (see Figure 3C). By 2021, the majority of DALYs attributable to Burkitt Lymphoma were observed in children aged 5–9 years, accounting for 32.3% of the total DALYs cases. Conversely, the 10–14-year age group consistently exhibited the lowest DALYs rate, representing only 16.5% of the cases (refer to Figures 3C, 4C). Gender disparities in DALYs were identified, with minimal differences observed between boys and girls under 1 year of age. However, a slight increase in DALYs was noted among girls aged 12–23 years. In contrast, boys aged 2–14 years demonstrated higher DALYs compared to their female counterparts, with the disparity being particularly significant in the 5–9 years age group (refer to Supplementary Figure S1C).

### Trends in sociodemographic index (SDI) regions for childhood Burkitt Lymphoma

**Figure 5:**
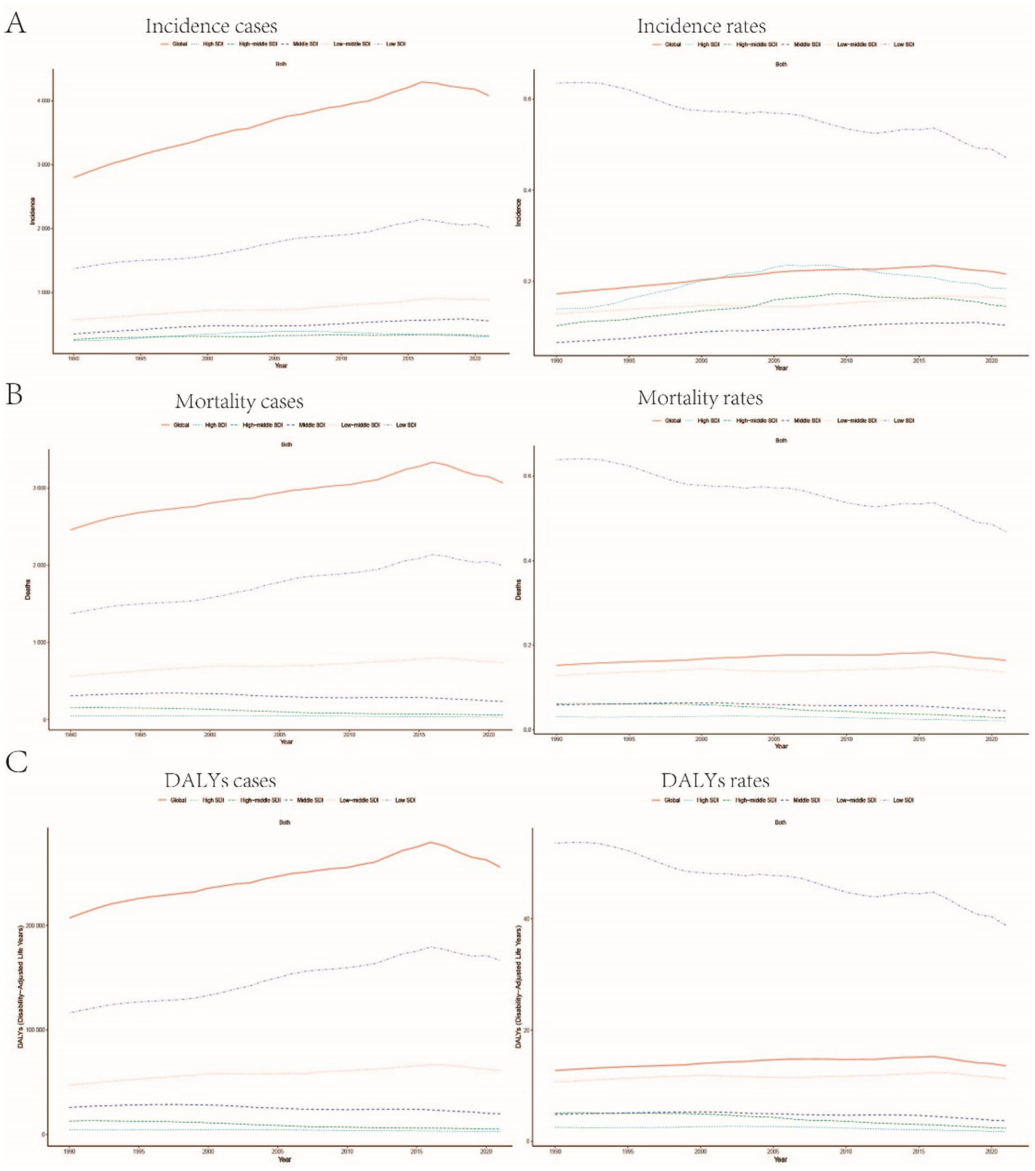
Trends in the incidence rate, mortality rate, and DALYs rate of childhood Burkitt Lymphoma across different Sociodemographic Index (SDI) regions: **(A–C)** Illustrate the changes in incidence rate, mortality rate, and DALYs rate globally and across regions with different SDI levels (high SDI, high-middle SDI, middle SDI, low-middle SDI, low SDI).

Compared to 1990, the incidence rates in 2021 increased across all regions except those with low SDI, with the most pronounced increase observed in middle-SDI regions. In contrast, mortality rates decreased in all regions except for low-middle SDI regions, with the most significant decline occurring in high-middle SDI regions. The trends in DALYs paralleled those observed in mortality rates. Furthermore, the EAPC for Burkitt Lymphoma mortality was highest in low-middle SDI regions at 0.265% (95% CI, −0.460 to 0.995), whereas the lowest EAPC was recorded in high-middle SDI regions at −2.802% (95% CI, −3.664 to −1.933) (refer to Supplementary Table S1 and Figure 5B). Similarly, the EAPC for DALYs was greatest in low-middle SDI regions at 0.247% (95% CI, −0.481 to 0.981) and lowest in high-middle SDI regions at −2.772% (95% CI, −3.621 to −1.915) (refer to Supplementary Table S2 and Figure 5C). In contrast to the incidence trends, the lowest EAPC for incidence was observed in low-SDI regions (−0.836, 95% CI, −1.637 to −0.028), while the highest was in middle-SDI regions (1.494, 95% CI, 0.729 to 2.265) (refer to Table 1 and Figure 5A).

### National trends in childhood Burkitt Lymphoma

#### Incidence

In 2021, Sub-Saharan West Africa reported the highest number of Burkitt Lymphoma cases globally, totaling 1,324 cases (95% UI: 700.382–1889.375). The region with the highest incidence rate was Sub-Saharan East Africa, with 0.684 cases per 100,000 individuals (95% UI: 0.387–0.999). Between 1990 and 2021, Sub-Saharan Southern Africa exhibited the most significant increase in incidence, with an annual percent change (APC) of 3.226% (95%CI: 2.092–4.373%). Conversely, Sub-Saharan Central Africa experienced the most pronounced decline in incidence, with an APC of −2.044% (95% CI: −3.334% to −0.738%). By 2021, the global incidence rate of Burkitt Lymphoma was 0.216 cases per 100,000 individuals (95% UI: 0.138–0.285), which exceeded the incidence rates in 16 regions but was lower than those in 5 regions (see Supplementary Table S3 and Figure 2).

#### Mortality

In 2021, Sub-Saharan West Africa reported the highest number of Burkitt Lymphoma-related fatalities globally, totaling 1,203 deaths (95% UI: 640.159–1681.108). The region with the highest mortality rate was Sub-Saharan East Africa, recording 0.676 deaths per 100,000 individuals (95% UI: 0.379–0.981). Between 1990 and 2021, Sub-Saharan Southern Africa exhibited the most rapid increase in mortality rate, with an annual percentage change (APC) of 2.874% (95% CI: 1.730–4.030%). In contrast, East Asia experienced the most pronounced decline in mortality rate, with an APC of −5.863% (95% CI: −6.643% to −5.075%). By 2021, the global mortality rate for Burkitt Lymphoma was 0.163 deaths per 100,000 individuals (95% UI: 0.100–0.216), which was higher than the rates in 18 regions but lower than those in 3 regions (refer to Supplementary Table S4 and Supplementary Figure S2).

### DALYs

In 2021, Sub-Saharan West Africa recorded the highest global burden of Burkitt Lymphoma-related DALYs, amounting to 100,922.799 years (95% UI: 53,347.220-141,467.692). Sub-Saharan East Africa exhibited the highest DALYs rate, with 55.826 years per 100,000 individuals (95% UI: 31.253–81.212). Between 1990 and 2021, Sub-Saharan Southern Africa experienced the most rapid increase in DALYs (APC = 2.876%; 95% CI: 1.735–4.029%), whereas East Asia demonstrated the most significant decrease (APC = -5.806%; 95% CI: −6.572% to −5.034%). By 2021, the global DALYs rate for Burkitt Lymphoma stood at 13.571 years per 100,000 individuals (95% UI: 8.236–17.994), surpassing the rates in 18 regions while remaining below those in 3 regions (see Supplementary Table S5 and Supplementary Figure S3 for details).

### BAPC projections

For childhood Burkitt Lymphoma, it is projected that by 2035, the global number of incident cases will reach 4,083 (95% uncertainty interval: 2,619.594–5,376.872), with the incidence rate (ASIR) decreasing to 0.22 (95% uncertainty interval: 0.138–0.285). The number of prevalent cases will increase to 25,495 (95% uncertainty interval: 15,619.075–35,376.872), while the prevalence rate (ASPR) will decrease to 1.70 (95% uncertainty interval: 1.00–2.40). The number of DALYs will decrease to 254,842.921 (95% uncertainty interval: 156,206.467–338,269.710), with the DALYs rate (ASDR) decreasing to 13.57 (95% uncertainty interval: 8.236–17.994). The number of deaths will decrease to 3,065 (95% uncertainty interval: 1,880.691–4,046.269), with the mortality rate (ASMR) decreasing to 0.16 (95% uncertainty interval: 0.100–0.216) (Supplementary Figures S4, S5).

### Inequality analysis

**Figure 6:**
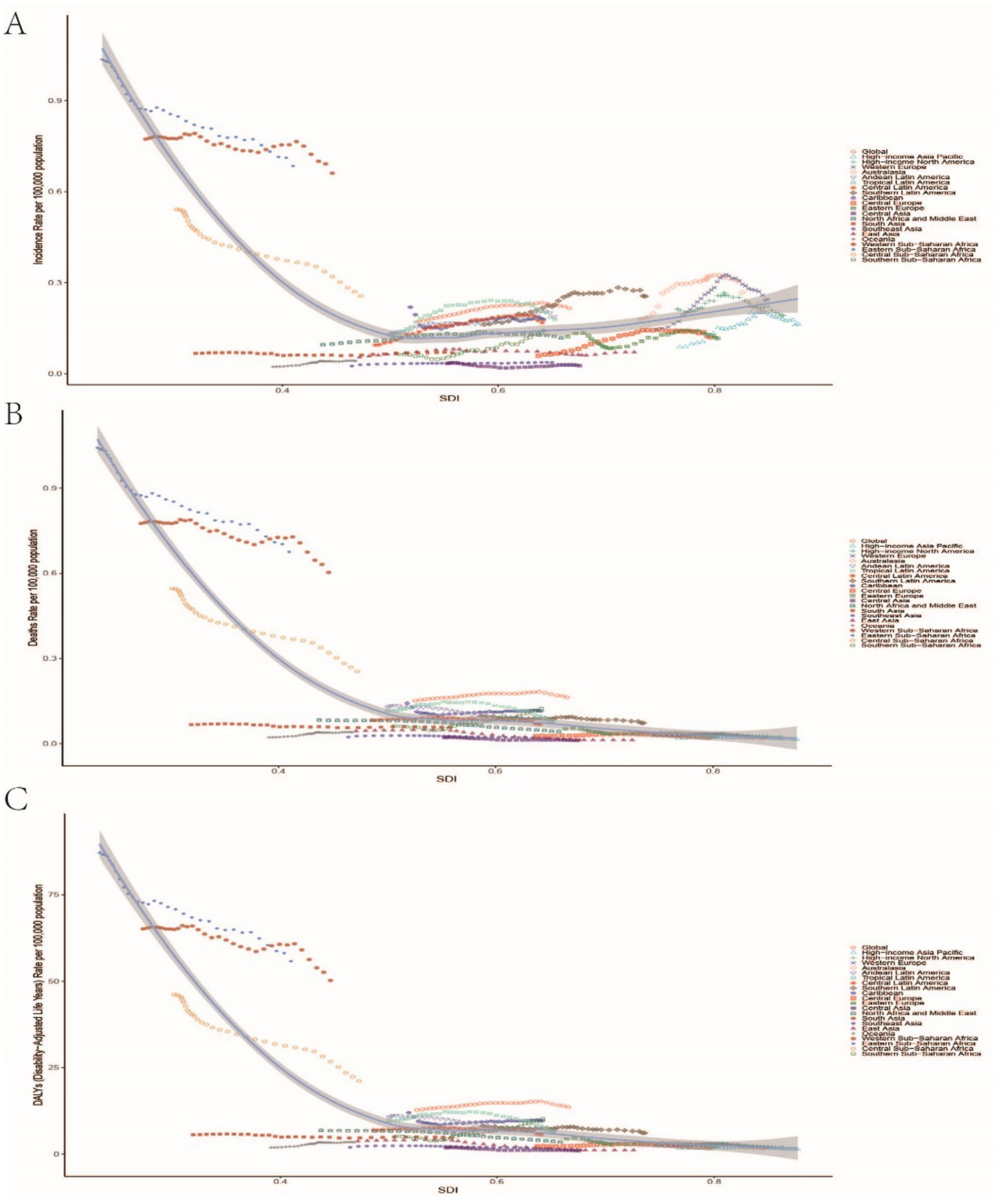
Relationship between the incidence rate, mortality rate, and DALYs rate of childhood Burkitt Lymphoma and the Sociodemographic Index (SDI). **(A–C)** Illustrate the correlations between SDI and incidence rate, mortality rate, nd DALYs rate, respectively.

From 1990 to 2021, global inequalities in DALYs and mortality rates for childhood Burkitt Lymphoma significantly worsened. In 1990, the slope index of inequality (SII) coefficient for DALYs was −32.82 (−37.70, −27.94), and the concentration index (CI) was −0.57 (−0.66, −0.46), indicating significant inequality with increased burden in low SDI regions. By 2021, the SII coefficient for DALYs decreased to −21.99 (−25.48, −18.50), and the CI decreased to −0.64 (−0.70, −0.56), showing significant inequality with increased burden in low SDI regions (Supplementary Figures S6A,B). Similarly, mortality rate inequality increased from an SII coefficient of −0.39 (−0.45, −0.34) and a CI of −0.57 (−0.66, −0.46) in 1990 to an SII coefficient of −0.27 (−0.31, −0.22) and a CI of −0.64 (−0.70, −0.56) in 2021, indicating significant increases in inequality with increased mortality burden in low SDI regions (Supplementary Figures S6C,D and Figure 6).

## Discussion

Pediatric Burkitt Lymphoma is an exceptionally aggressive subtype of non-Hodgkin lymphoma, accounting for 30–50% of pediatric lymphoma cases and ranking as the fourth most common malignancy in children. Over the past three decades, the implementation of short-term intensive multi-drug chemotherapy has markedly improved survival rates. However, the associated toxicity and the potential for developing resistance highlight the critical need for the development of targeted therapies with reduced toxicity (16). Between 1990 and 2021, the global incidence, mortality, and Disability-Adjusted Life Years (DALYs) related to Burkitt Lymphoma initially increased before subsequently declining, a pattern largely attributable to advancements in medical technology and a deeper understanding of the disease’s pathophysiology. Progress in early diagnosis and therapeutic strategies, including the use of multi-drug combination immunochemotherapy regimens, has resulted in favorable prognoses for the majority of patients (17). In high-resource countries, risk-adapted short-term intensive chemotherapy has achieved cure rates exceeding 85% for pediatric B-cell non-Hodgkin lymphoma. Recent large-scale pediatric cooperative group trials have reduced the intensity of cytotoxic therapy and incorporated targeted treatments, such as rituximab, thereby further improving patient outcomes. Nonetheless, innovative therapeutic strategies remain essential for high-risk patients (18). In low- and middle-income countries, advancements in early and accurate diagnosis, reductions in treatment-related toxic fatalities, and the prevention of therapy interruptions can significantly optimize therapeutic outcomes, improve survival rates, and decrease mortality and disability-adjusted life years (DALYs). Significant therapeutic advancements, such as the combination of high-dose methotrexate with rituximab, have become crucial in enhancing patient prognoses (19). Targeted therapies and immunotherapies are expanding the spectrum of therapeutic options available for the treatment of relapsed and refractory lymphomas. Innovative pharmaceuticals, including monoclonal antibodies, immune checkpoint inhibitors, and small molecules, are transforming the management of these conditions. Notably, CAR-T cell therapy and immune checkpoint inhibitors, such as nivolumab, demonstrate significant efficacy and safety, offering novel strategies for addressing relapsed and refractory cases (20). The targeting of B-cell receptor signaling pathways with agents like ibrutinib, alongside apoptosis-targeting drugs such as venetoclax, is the focus of extensive research and clinical application. These efforts aim to enhance treatment efficacy by directly targeting tumor cell pathways or modulating the immune response within the tumor microenvironment (8). The refinement of chemotherapy protocols, coupled with the introduction of advanced targeted immunotherapies, signals a promising future characterized by improved survival rates and more personalized treatment regimens. Ongoing clinical trials and forthcoming regulatory approvals are expected to drive therapeutic advancements, offering renewed hope to patients. Intensifying global efforts in the screening and surveillance of pediatric tumors is crucial for the early detection and intervention of Burkitt Lymphoma, which can significantly reduce the incidence of missed and erroneous diagnoses and thereby improve cure rates. Recent advancements in treatment methodologies for pediatric acute lymphoblastic leukemia have markedly increased global cure rates, establishing it as one of the most curable human malignancies. Progress in global screening and monitoring, alongside enhanced therapeutic strategies, is continually improving cure rates for childhood cancers and enhancing the quality of life for patients. These advancements are largely dependent on international collaborative research and network building, which aim to extend the successful treatment paradigms of high-income countries to low- and middle-income regions (21). The advancement of molecularly targeted anticancer therapies presents a promising avenue for achieving more effective treatments with reduced side effects, thereby necessitating collaboration among industry, academia, and the healthcare sector (22). While less aggressive treatment regimens have the potential to produce comparable outcomes with fewer adverse events, the evidence supporting the relative efficacy of current interventions remains inconclusive due to limited study sizes, capacity constraints, and variability. Consequently, further research is essential to determine the optimal treatment protocols (23). Health education and increased public awareness play a crucial role in encouraging parents and healthcare professionals to maintain vigilance regarding children’s health, facilitating early detection and intervention, and thereby reducing the disease burden (24). Support from institutions such as school health centers, which provide accessible health services within the educational setting, can help alleviate the burden on both families and healthcare systems (25). Recent research and policy recommendations emphasize the critical importance of implementing comprehensive and preventive strategies within children’s health policies. Key components such as basic income support, familial assistance, and universal health insurance are essential for significantly improving the health and well-being of children and adolescents, a necessity that was particularly underscored during the COVID-19 pandemic (26). Despite a general decline in the global burden of Burkitt Lymphoma, significant disparities persist across regions and countries with varying Socio-Demographic Index (SDI) levels. In low-SDI areas, the incidence rate decreases slowly or may even increase, reflecting insufficient detection and diagnostic capabilities in these regions (9). Similarly, studies on chronic myeloid leukemia indicate that although the global disease burden is decreasing, incidence and mortality rates are rising in low-SDI countries, primarily due to population growth (27). Research on the global burden of kidney cancer reveals a decline in incidence rates in high-Socio-Demographic Index (SDI) countries, contrasted by an increase in low and middle-SDI regions (28). In low-SDI areas, the lack of advanced diagnostic equipment and skilled healthcare professionals hinders early diagnosis and treatment of Burkitt Lymphoma, resulting in a heavier disease burden. Patients often receive care only at advanced stages, complicating treatment efforts (29). Despite significant therapeutic advancements in recent years, uncertainties persist, particularly among pediatric patients. Less aggressive treatment regimens may yield similar outcomes with fewer adverse events; however, the evidence is weak due to small sample sizes, limited capacity, and variability (23). The growing application of artificial intelligence algorithms in medical image classification has made computer-aided diagnostic systems indispensable for disease diagnosis. Nevertheless, in remote areas where essential equipment and skilled personnel are scarce, data gaps impede the effective use of these systems, highlighting the urgent need for alternative data acquisition methods to address this global issue (30). In regions with low Socio-Demographic Index (SDI), inadequate public health infrastructure and limited health education exacerbate the disease burden, with insufficient resources to support health education and disease prevention, as demonstrated by the high prevalence of severe vitamin A deficiency among children in these areas (31). Enhancing public health infrastructure and health education in low-SDI regions, along with raising public awareness about disease prevention and early detection, are crucial strategies to mitigate the disease burden. Cross-departmental collaboration and policy support can significantly improve health outcomes and reduce health disparities (32). In regions with a high SDI, the availability of extensive medical resources, advanced technology, and robust public health systems significantly enhances the prevention and management of Burkitt Lymphoma. The combination of timely and accurate diagnosis, intensive chemotherapy, and comprehensive supportive care leads to high cure rates. Moreover, patients benefit from innovative treatment options and personalized care plans, which considerably reduce the disease burden (11). In contrast, low-SDI regions face challenges such as delayed diagnoses, treatment interruptions, and mortality due to treatment toxicity, resulting in a higher disease burden and lower cure rates. Despite the positive outcomes observed in high-SDI regions, the pronounced global disparities in disease burden highlight the urgent need for international collaboration and resource sharing to address these inequities (6). The incidence and mortality rates of pediatric Burkitt Lymphoma exhibit significant disparities across various age groups and genders. Children aged 5 to 9 years, in particular, show the highest incidence and mortality rates, likely due to their developing immune systems, which increase their susceptibility to the disease (33). This age group is also at risk for other health issues, such as Lyme disease, with the highest incidence possibly linked to their increased activity levels and greater exposure to pathogens (34). Although their exposure to carcinogenic factors is limited due to a narrower range of activities, the health risks for this age group should not be underestimated. Between 1990 and 2016, the mortality rate among children aged 5–14 decreased by 51%; however, this demographic continues to require focused attention and intervention (35). In low- and middle-income countries, children aged 5–14 face relatively higher mortality risks, highlighting the need for global health policies to prioritize this group (36). Despite children aged 5–9 years being less frequently exposed to carcinogens, the rapid progression of cancer and the complexities associated with treatment upon diagnosis may be linked to a “vulnerable window” during their growth and development, which makes target organs more susceptible to damage. Therefore, enhancing health surveillance and early intervention is crucial (37). Male children generally exhibit higher incidence and mortality rates compared to females, a disparity that may arise from genetic factors, hormonal differences, and varying levels of exposure to carcinogens. Boys show significantly higher incidence rates of cancers such as leukemia, lymphoma, and central nervous system tumors compared to girls. These gender disparities are primarily attributed to endogenous risk factors, with boys being more susceptible to neuroblastoma and liver tumors, while girls occasionally exhibit higher incidences of kidney tumors. Gender differences manifest distinct patterns across various cancer types (38). Biological factors, including levels of sex hormones, play a significant role in the development and functioning of children’s immune systems, underscoring the importance of gender in the incidence and mortality of pediatric cancers. Further research is necessary to elucidate the underlying mechanisms to more effectively address these disparities (39). Studies have demonstrated that certain genetic susceptibility genes exhibit higher activity in male children, thereby increasing their risk of developing Burkitt Lymphoma. Although autoimmune diseases are more prevalent in females, the gender disparity in childhood onset is not pronounced, possibly due to differing hormonal environments during childhood. The expression levels of miRNA on sex chromosomes and gender differences are associated with the development of autoimmune diseases, offering insights into understanding gender disparities (38). Male children are more susceptible to encountering carcinogens, including specific chemicals and radiation, during their developmental years, which increases their risk of disease. Research indicates that early exposure to carcinogenic chemicals and ionizing radiation significantly elevates the likelihood of developing cancer later in life. This is attributed to the rapid cell growth and organ system development occurring in children and adolescents, combined with the long latency periods characteristic of many cancers (40). Exposure to environmental pollutants and harmful anthropogenic factors, such as radiation, can further exacerbate the risk of cancer in children. A study conducted in Poland’s Silesia Province underscored the significance of exposure duration in cancer risk assessment, revealing variations in cancer incidence based on age, gender, and region. The findings suggest that prolonged exposure to environmental pollutants may particularly increase cancer risk in children, with a notable impact on boys (41). In developing public health strategies for pediatric Burkitt Lymphoma, it is imperative to consider factors such as regional medical resources, levels of economic development, and the prevalence of the disease. Tailored and targeted prevention and control measures are crucial. Investment in medical infrastructure and the advancement of medical technology are critical for enhancing diagnostic and treatment capabilities in regions with low SDI, which necessitates professional training to strengthen medical teams. The transformation of tuberculosis service delivery in China illustrates the importance of equipping healthcare systems with adequate resources and well-trained personnel (42). Similarly, Nepal’s emphasis on investing in mental health services underscores the necessity for broader resource allocation to improve medical outcomes in resource-constrained areas (43). Over the past 50 years, Oman’s healthcare system innovation has provided a valuable model. However, challenges persist in sustaining these achievements due to population growth and financial dependency, highlighting the necessity for continued investment and adaptable healthcare systems. Strengthening medical infrastructure, advancing medical technology, and investing in the training of healthcare personnel are crucial strategies for enhancing medical services in regions with low Socio-Demographic Index (SDI), which is essential for combating diseases such as Burkitt Lymphoma and ensuring high-quality medical care (44). Enhancing health education and public awareness campaigns is critical for increasing awareness of cancer screening. National early detection programs for breast and cervical cancers have successfully increased the utilization of screening services through education and the resolution of barriers. Similar strategies could be applied to the prevention and early detection of pediatric cancers. Increasing parental and community awareness about the importance of regular pediatric check-ups and screenings can facilitate early detection, diagnosis, and treatment (45). In summary, the examination of global burden trends associated with pediatric Burkitt Lymphoma offers critical insights into the epidemiological characteristics and determinants of the disease, thereby facilitating the development of effective prevention and control strategies. Enhanced international collaboration and resource allocation have the potential to significantly advance prevention initiatives, mitigate the disease burden, and enhance both survival rates and the quality of life for affected patients.

## Data Availability

The datasets presented in this study can be found in online repositories. The names of the repository/repositories and accession number(s) can be found in the article/Supplementary material.

## Footnotes

^1^ https://vizhub.healthdata.org/gbd-results/

## Supplementary material

SUPPLEMENTARY FIGURE S1 Incidence rate of childhood Burkitt Lymphoma stratified by gender and age: **(A)** Shows the absolute incidence numbers and incidence rates per 100,000 population across different age groups, separated by females and males (including incidence rate and 95% uncertainty interval, as well as case numbers and 95% uncertainty interval). **(B,C)** Represent similarly stratified data for mortality rate and DALYs rate by gender and age.

SUPPLEMENTARY FIGURE S2 Data related to the mortality of childhood Burkitt Lymphoma: **(A)** Number of mortality cases; **(B)** Mortality rate; **(C)** Annual percentage change (EAPC) in mortality rate.

SUPPLEMENTARY FIGURE S3 Data related to DALYs for childhood Burkitt Lymphoma: **(A)** Number of DALYs cases; **(B)** DALYs rate; **(C)** Annual percentage change (EAPC) in DALYs rate.

SUPPLEMENTARY FIGURE S4 Temporal trends of incident cases **(A)**, DALYs **(B)**, and deaths **(C)** for childhood Burkitt Lymphoma from 1990 to 2035. Red lines represent the ASIR, ASPR, ASDR, and ASMR predicted by the Bayesian Age-Period-Cohort (BAPC) model. BAPC: Bayesian Age-Period-Cohort.

SUPPLEMENTARY FIGURE S5 Temporal trends of ASIR **(A)**, ASDR **(B)**, and ASMR **(C)** for childhood Burkitt Lymphoma from 1990 to 2035. Red lines represent the ASIR, ASPR, ASDR, and ASMR predicted by the Bayesian Age-Period-Cohort (BAPC) model. BAPC: Bayesian Age-Period-Cohort.

SUPPLEMENTARY FIGURE S6 Health inequality regression curves and concentration curves for global childhood Burkitt Lymphoma in 1990 and 2021. **(A,C)** Show the Slope Index of Inequality, depicting the relationship between SDI and age-standardized DALYs rate, with points representing individual countries and regions stratified by population size. **(B,D)** Show the Concentration Index, quantifying relative inequality by integrating the Lorenz curve, aligning DALYs distribution with population distribution stratified by SDI. Blue represents data from 1990, and red represents data from 2021.
