# Supplementary figures and images for: Global, regional, and national epidemiology of childhood Burkitt Lymphoma from 1990 to 2021: statistical analysis of incidence, mortality, and DALYs
Source: Front Public Health. 2025 Jul 16;13:1560003. doi: 10.3389/fpubh.2025.1560003 (PMC12307453; doi:10.3389/fpubh.2025.1560003)

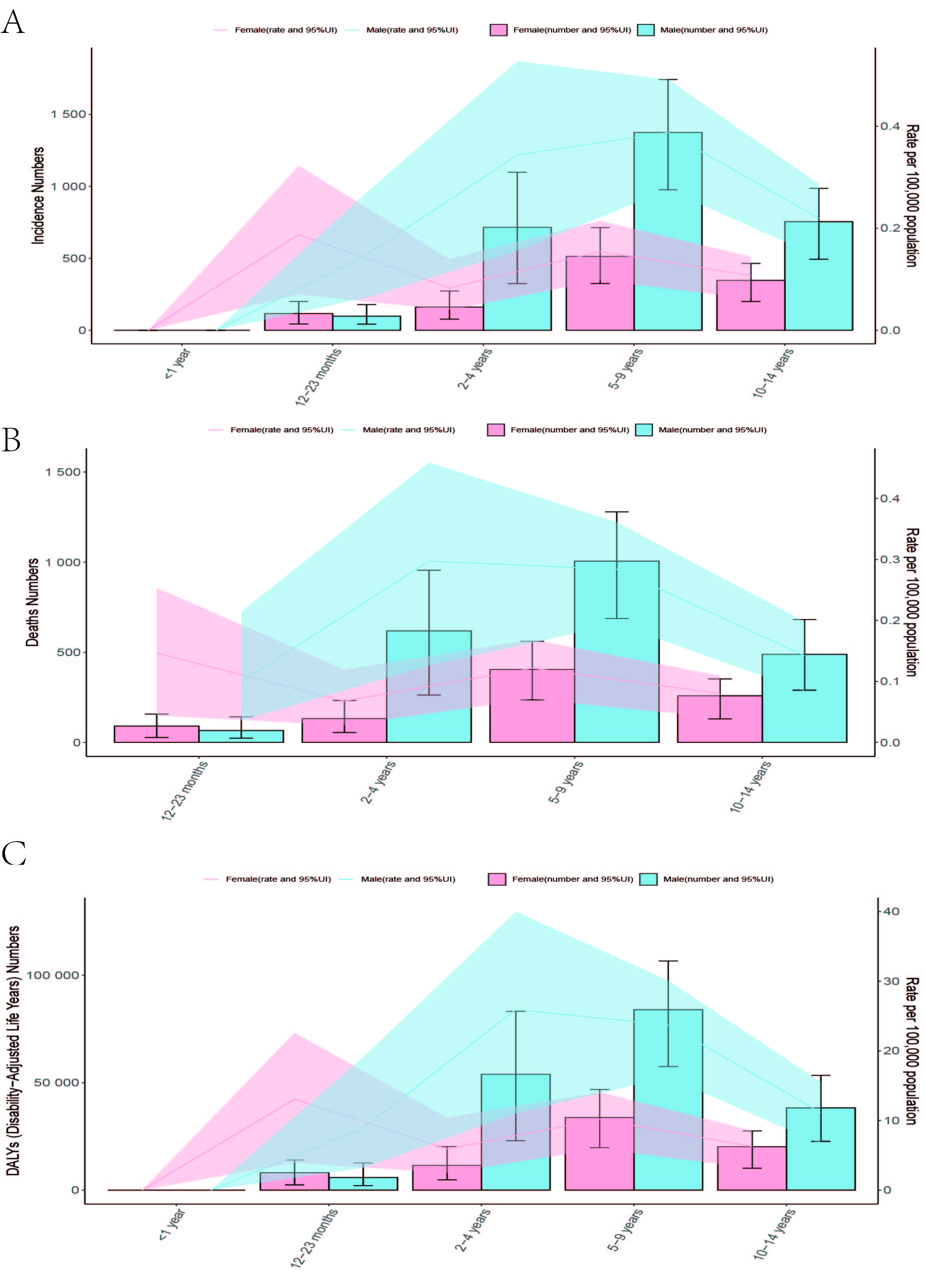

Supplement: SUPPLEMENTARY FIGURE S1 — Incidence rate of childhood Burkitt Lymphoma stratified by gender and age: (A) Shows the absolute incidence numbers and incidence rates per 100,000 population across different age groups, separated by females and males (including incidence rate and 95% uncertainty interval, as well as case numbers and 95% uncertainty interval). (B,C) Represent similarly stratified data for mortality rate and DALYs rate by gender and age. [file Image_1.JPEG]

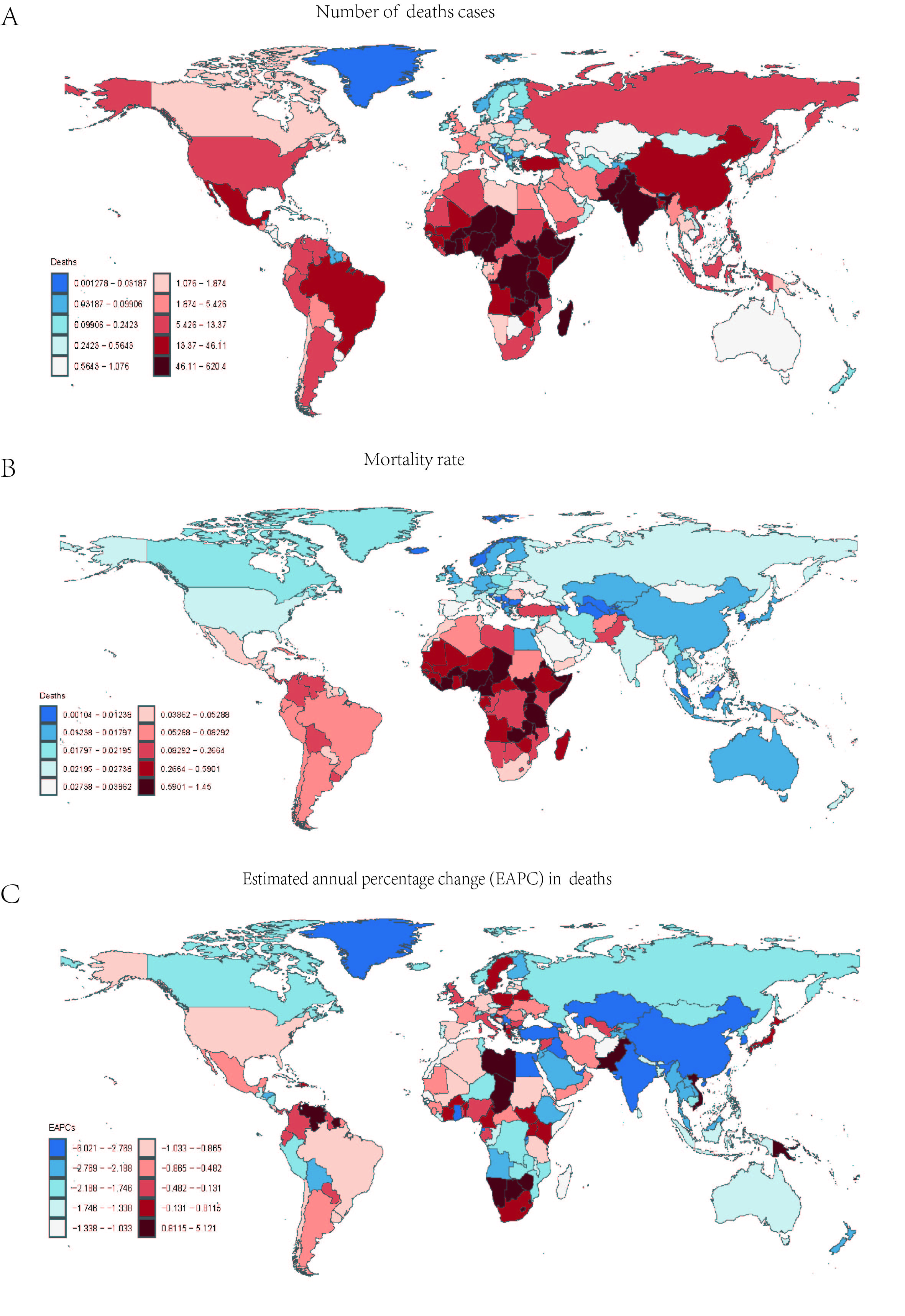

Supplement: SUPPLEMENTARY FIGURE S2 — Data related to the mortality of childhood Burkitt Lymphoma: (A) Number of mortality cases; (B) Mortality rate; (C) Annual percentage change (EAPC) in mortality rate. [file Image_2.JPEG]

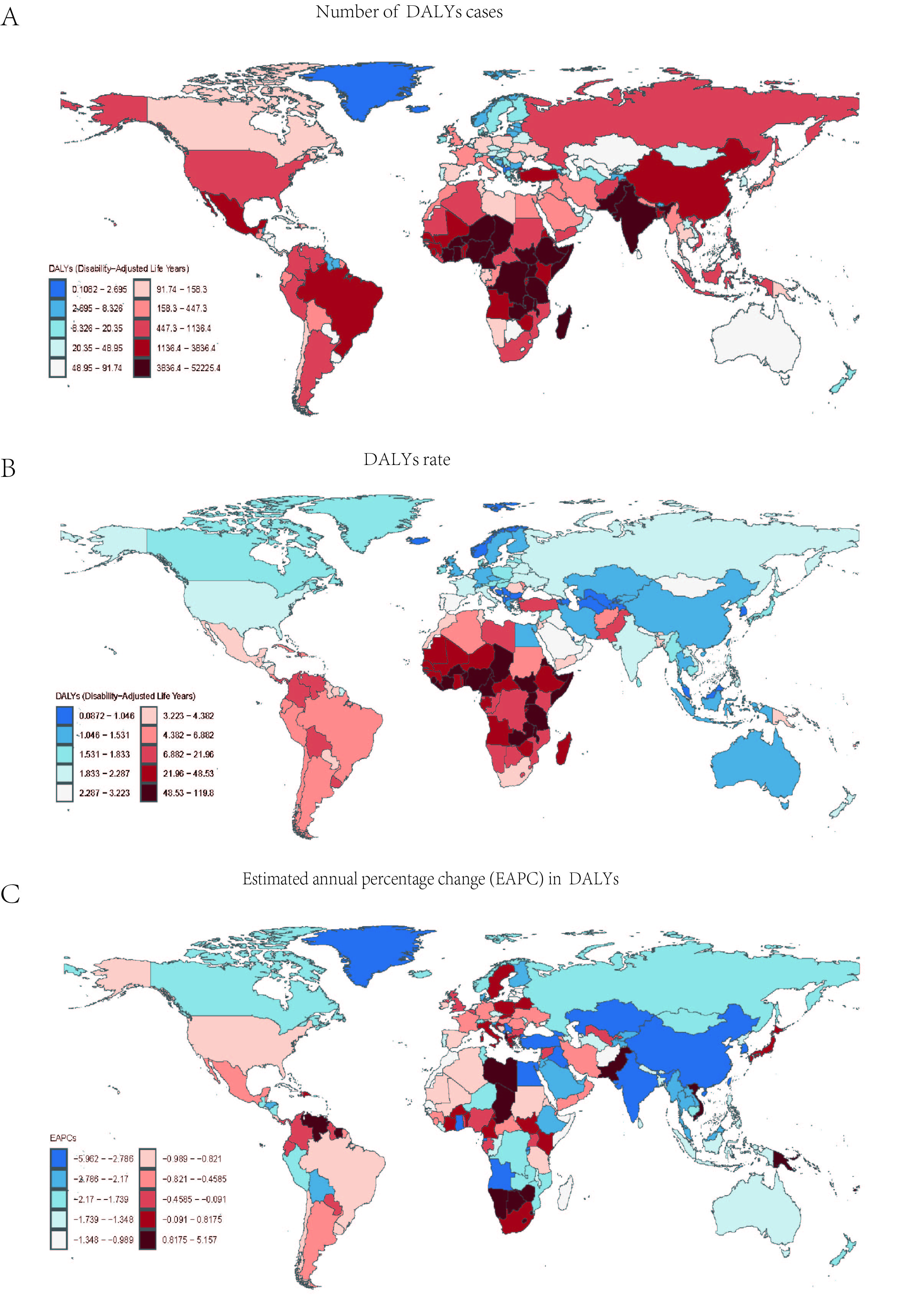

Supplement: SUPPLEMENTARY FIGURE S3 — Data related to DALYs for childhood Burkitt Lymphoma: (A) Number of DALYs cases; (B) DALYs rate; (C) Annual percentage change (EAPC) in DALYs rate. [file Image_3.JPEG]

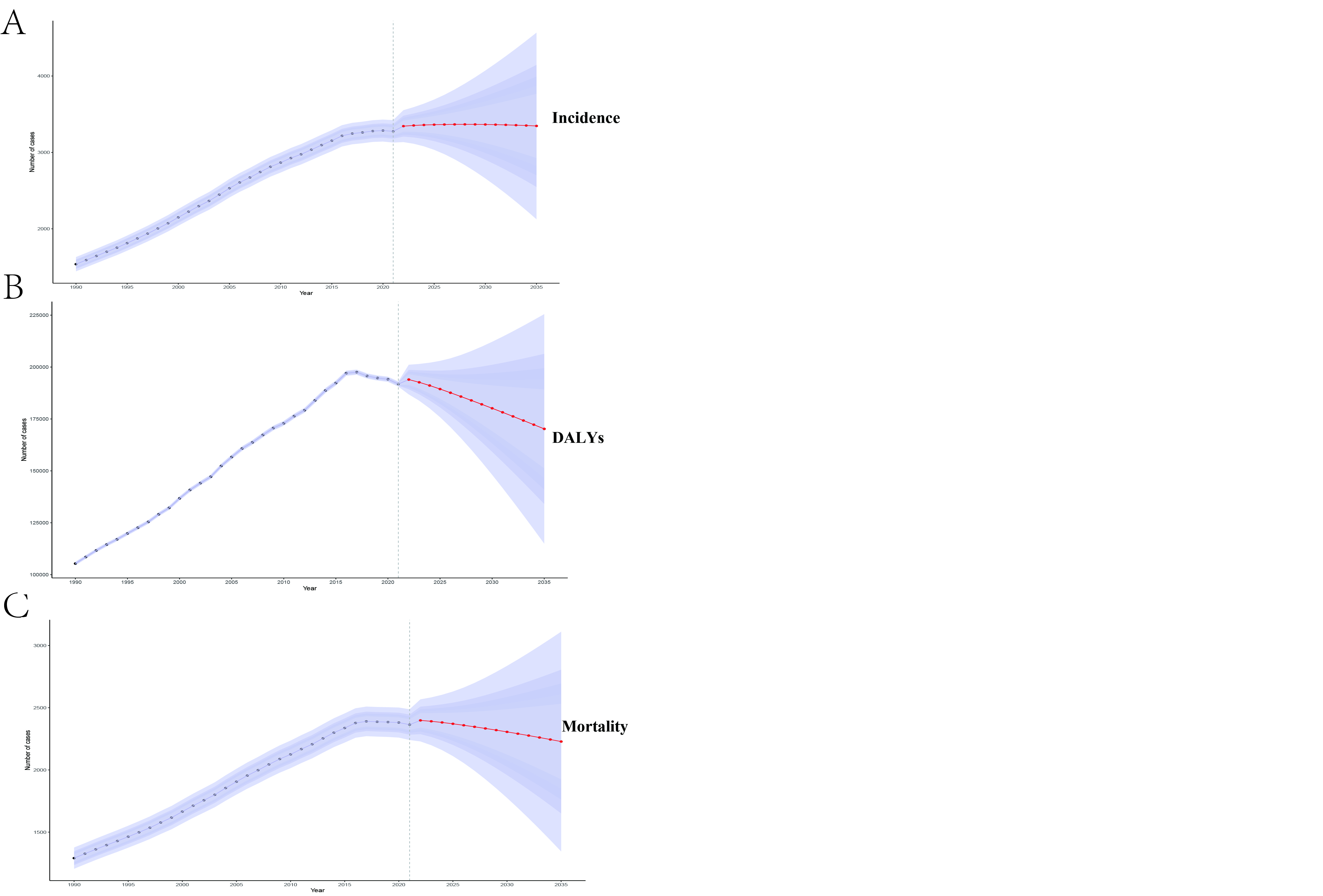

Supplement: SUPPLEMENTARY FIGURE S4 — Temporal trends of incident cases (A), DALYs (B), and deaths (C) for childhood Burkitt Lymphoma from 1990 to 2035. Red lines represent the ASIR, ASPR, ASDR, and ASMR predicted by the Bayesian Age-Period-Cohort (BAPC) model. BAPC: Bayesian Age-Period-Cohort. [file Image_4.JPEG]

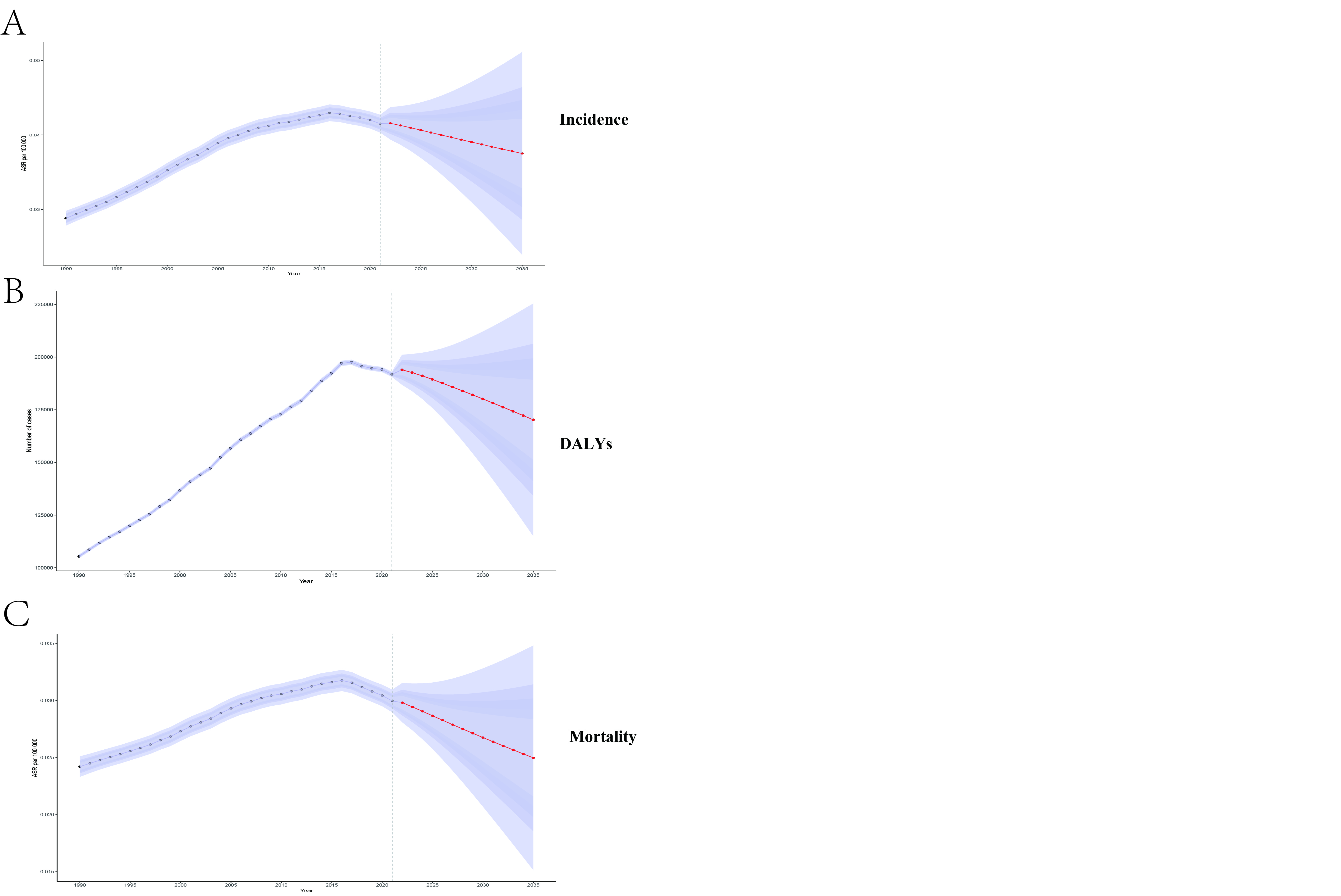

Supplement: SUPPLEMENTARY FIGURE S5 — Temporal trends of ASIR (A), ASDR (B), and ASMR (C) for childhood Burkitt Lymphoma from 1990 to 2035. Red lines represent the ASIR, ASPR, ASDR, and ASMR predicted by the Bayesian Age-Period-Cohort (BAPC) model. BAPC: Bayesian Age-Period-Cohort. [file Image_5.JPEG]

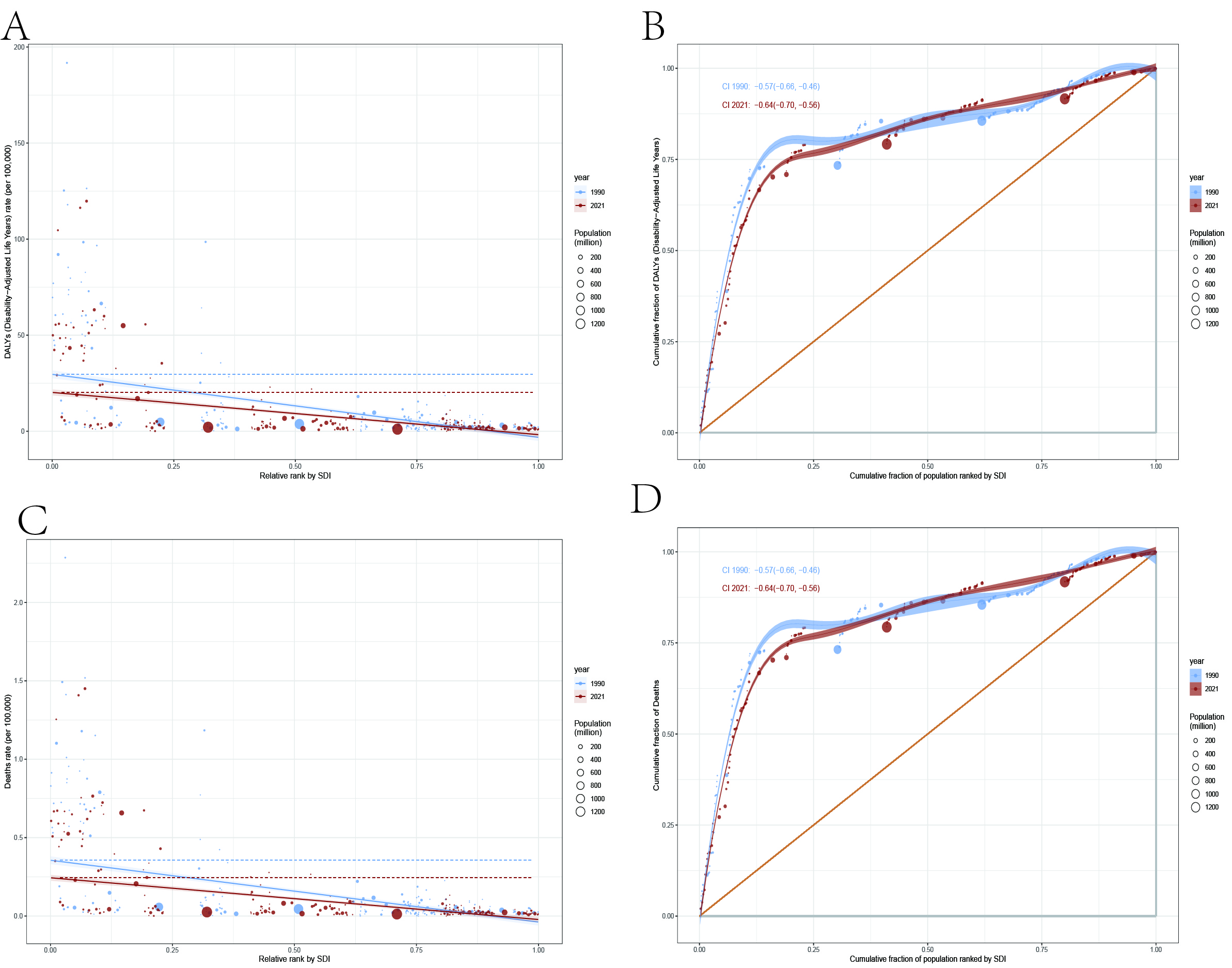

Supplement: SUPPLEMENTARY FIGURE S6 — Health inequality regression curves and concentration curves for global childhood Burkitt Lymphoma in 1990 and 2021. (A,C) Show the Slope Index of Inequality, depicting the relationship between SDI and age-standardized DALYs rate, with points representing individual countries and regions stratified by population size. (B,D) Show the Concentration Index, quantifying relative inequality by integrating the Lorenz curve, aligning DALYs distribution with population distribution stratified by SDI. Blue represents data from 1990, and red represents data from 2021. [file Image_6.JPEG]
