# Supplementary material for: Global, regional, and national epidemiology of childhood Burkitt Lymphoma from 1990 to 2021: statistical analysis of incidence, mortality, and DALYs
Source: Front Public Health. 2025 Jul 16;13:1560003. doi: 10.3389/fpubh.2025.1560003 (PMC12307453; doi:10.3389/fpubh.2025.1560003)
Supplement: Supplementary file 7 [file Table_1.docx]

Table S1: The number of deaths and mortality rates of Burkitt lymphoma in children globally, in 5 SDI regions, and in 21 regions in 1990 and 2021, as well as the annual percentage change (EAPC) from 1990 to 2021.

| Location |  | Rate per 100,000 (95% UI) |  |  |  |
| --- | --- | --- | --- | --- | --- |
|  | 1990 |  | 2021 |  | 1990-2021 |
|  | Number of deaths | Mortality rate | Number of deaths | Mortality rate | EAPC |
| Global | 2457.854(1278.712,3631.749) | 0.152(0.079,0.224) | 3065.538(1880.691,4046.269) | 0.163(0.100,0.216) | 0.402(-0.375,1.185) |
| Regions |  |  |  |  |  |
| East Asia | 134.683(55.351,226.590) | 0.044(0.018,0.074) | 31.828(17.010,57.007) | 0.013(0.007,0.023) | -5.863(-6.643,-5.075) |
| Southeast Asia | 36.992(11.879,73.755) | 0.023(0.007,0.047) | 32.355(15.956,54.288) | 0.020(0.010,0.034) | -1.213(-2.210,-0.206) |
| Oceania | 0.560(0.183,1.627) | 0.022(0.007,0.064) | 1.956(0.469,5.564) | 0.040(0.010,0.115) | 2.062(1.762,2.364) |
| Central Asia | 5.124(2.889,9.050) | 0.022(0.012,0.039) | 2.948(1.732,4.903) | 0.011(0.007,0.019) | -2.656(-3.617,-1.686) |
| Central Europe | 7.431(4.422,14.052) | 0.027(0.016,0.051) | 3.526(1.649,5.212) | 0.021(0.010,0.031) | -0.578(-1.720,0.578) |
| Eastern Europe | 25.439(14.878,39.374) | 0.052(0.031,0.081) | 8.830(3.774,13.150) | 0.025(0.011,0.038) | -1.719(-2.533,-0.898) |
| High-income Asia Pacific | 6.620(3.365,10.677) | 0.019(0.010,0.031) | 3.348(1.510,4.815) | 0.015(0.007,0.022) | -0.754(-1.878,0.382) |
| Australasia | 1.302(0.806,2.071) | 0.030(0.019,0.048) | 0.986(0.483,1.581) | 0.018(0.009,0.029) | -1.772(-2.835,-0.697) |
| Western Europe | 19.267(13.237,30.536) | 0.029(0.020,0.045) | 14.537(6.230,21.689) | 0.022(0.010,0.033) | -0.662(-1.727,0.416) |
| Southern Latin America | 13.667(8.602,20.601) | 0.098(0.062,0.148) | 10.138(6.132,15.361) | 0.073(0.044,0.111) | -0.598(-1.461,0.272) |
| High-income North America | 21.049(14.593,29.252) | 0.037(0.026,0.051) | 14.458(9.079,19.785) | 0.023(0.014,0.031) | -0.966(-2.280,0.365) |
| Caribbean | 15.072(7.228,34.944) | 0.143(0.069,0.329) | 12.333(4.801,30.417) | 0.116(0.045,0.287) | 0.162(-0.337,0.665) |
| Andean Latin America | 17.645(9.237,31.258) | 0.129(0.067,0.228) | 13.115(6.650,23.129) | 0.078(0.039,0.137) | -1.844(-2.642,-1.039) |
| Central Latin America | 49.626(33.461,77.890) | 0.084(0.056,0.131) | 40.445(23.525,56.784) | 0.068(0.039,0.096) | -0.432(-1.105,0.247) |
| Tropical Latin America | 57.624(39.013,86.879) | 0.115(0.077,0.174) | 37.514(20.165,52.520) | 0.080(0.043,0.112) | -0.929(-1.690,-0.161) |
| North Africa and Middle East | 108.661(57.002,198.765) | 0.084(0.044,0.153) | 75.118(45.062,128.029) | 0.043(0.026,0.074) | -2.099(-3.073,-1.116) |
| South Asia | 273.671(92.944,528.719) | 0.067(0.023,0.130) | 276.004(141.968,474.938) | 0.058(0.030,0.100) | -0.621(-1.068,-0.172) |
| Central Sub-Saharan Africa | 132.574(34.084,246.951) | 0.546(0.147,1.006) | 139.090(62.814,226.830) | 0.255(0.115,0.415) | -2.070(-3.355,-0.767) |
| Eastern Sub-Saharan Africa | 877.378(359.023,1389.714) | 1.042(0.430,1.643) | 1115.920(624.278,1621.345) | 0.676(0.379,0.981) | -1.300(-2.026,-0.570) |
| Southern Sub-Saharan Africa | 11.970(5.979,20.777) | 0.063(0.031,0.108) | 27.905(12.916,50.503) | 0.124(0.057,0.225) | 2.874(1.730,4.030) |
| Western Sub-Saharan Africa | 641.497(266.079,997.650) | 0.776(0.329,1.196) | 1203.186(640.159,1681.108) | 0.603(0.323,0.840) | -0.619(-1.522,0.292) |
| SDI |  |  |  |  |  |
| High-middle SDI | 155.068(88.663,237.393) | 0.061(0.035,0.093) | 62.760(40.510,84.877) | 0.028(0.018,0.038) | -2.802(-3.664,-1.933) |
| High SDI | 52.873(38.352,74.884) | 0.030(0.022,0.043) | 34.227(17.817,45.664) | 0.021(0.011,0.028) | -1.039(-2.162,0.097) |
| Low-middle SDI | 563.358(284.411,856.525) | 0.128(0.065,0.194) | 735.475(475.026,1023.153) | 0.136(0.088,0.190) | 0.265(-0.460,0.995) |
| Low SDI | 1374.342(593.154,2190.104) | 0.639(0.279,1.012) | 1994.051(1115.540,2740.304) | 0.468(0.262,0.642) | -0.869(-1.668,-0.063) |
| Middle SDI | 310.769(188.939,425.225) | 0.058(0.035,0.079) | 237.354(142.396,316.292) | 0.044(0.026,0.059) | -0.898(-1.640,-0.151) |
