# Supplementary material for: Global, regional, and national epidemiology of childhood Burkitt Lymphoma from 1990 to 2021: statistical analysis of incidence, mortality, and DALYs
Source: Front Public Health. 2025 Jul 16;13:1560003. doi: 10.3389/fpubh.2025.1560003 (PMC12307453; doi:10.3389/fpubh.2025.1560003)
Supplement: Supplementary file 8 [file Table_2.docx]

Table S2: The number of DALYs and DALYs rates of Burkitt lymphoma in children globally, in 5 SDI regions, and in 21 regions in 1990 and 2021, as well as the annual percentage change (EAPC) from 1990 to 2021.

| Location |  | Rate per 100,000 (95% UI) |  |  |  |
| --- | --- | --- | --- | --- | --- |
|  | 1990 |  | 2021 |  | 1990-2021 |
|  | Number of DALYs | DALYs rate | Number of DALYs | DALYs rate | EAPC |
| Global | 207119.995(107272.072,306869.858) | 12.725(6.604,18.836) | 255842.921(156206.467,338269.710) | 13.571(8.236,17.994) | 0.375(-0.402,1.158) |
| Regions |  |  |  |  |  |
| East Asia | 11381.772(4630.373,19239.435) | 3.719(1.515,6.280) | 2724.160(1450.192,4884.071) | 1.093(0.572,1.952) | -5.806(-6.572,-5.034) |
| Southeast Asia | 3092.260(980.117,6205.168) | 1.942(0.612,3.907) | 2690.483(1318.525,4520.537) | 1.659(0.809,2.793) | -1.223(-2.215,-0.221) |
| Oceania | 47.633(15.527,138.546) | 1.859(0.609,5.398) | 166.054(39.936,472.169) | 3.396(0.815,9.653) | 2.057(1.785,2.331) |
| Central Asia | 430.550(242.943,761.662) | 1.835(1.036,3.235) | 247.096(144.828,410.945) | 0.954(0.560,1.586) | -2.649(-3.597,-1.692) |
| Central Europe | 616.632(366.960,1170.494) | 2.212(1.314,4.215) | 297.783(138.920,441.471) | 1.773(0.826,2.634) | -0.519(-1.651,0.625) |
| Eastern Europe | 2120.791(1238.865,3288.688) | 4.345(2.535,6.741) | 738.704(314.366,1098.870) | 2.127(0.897,3.176) | -1.684(-2.491,-0.870) |
| High-income Asia Pacific | 552.167(279.933,889.784) | 1.612(0.819,2.608) | 289.535(129.985,414.175) | 1.318(0.588,1.901) | -0.630(-1.753,0.506) |
| Australasia | 109.942(68.023,174.465) | 2.546(1.574,4.044) | 85.376(41.453,137.883) | 1.541(0.744,2.495) | -1.670(-2.743,-0.586) |
| Western Europe | 1617.399(1108.594,2562.633) | 2.401(1.645,3.804) | 1262.294(540.247,1891.933) | 1.928(0.825,2.900) | -0.541(-1.606,0.536) |
| Southern Latin America | 1135.945(714.921,1714.190) | 8.139(5.123,12.284) | 847.811(512.028,1284.647) | 6.097(3.670,9.282) | -0.567(-1.422,0.295) |
| High-income North America | 1769.481(1227.903,2474.720) | 3.078(2.137,4.305) | 1230.993(769.341,1682.047) | 1.942(1.207,2.655) | -0.914(-2.223,0.412) |
| Caribbean | 1269.326(602.044,2975.367) | 11.942(5.698,27.839) | 1034.058(398.081,2566.712) | 9.669(3.683,24.141) | 0.153(-0.338,0.647) |
| Andean Latin America | 1469.067(765.997,2612.345) | 10.653(5.558,18.928) | 1092.121(552.474,1927.725) | 6.432(3.252,11.356) | -1.839(-2.627,-1.044) |
| Central Latin America | 4148.423(2795.303,6520.886) | 6.944(4.682,10.909) | 3371.488(1958.766,4735.282) | 5.640(3.264,7.943) | -0.422(-1.088,0.249) |
| Tropical Latin America | 4807.895(3252.923,7257.249) | 9.553(6.435,14.462) | 3113.837(1669.695,4362.501) | 6.622(3.548,9.281) | -0.945(-1.697,-0.186) |
| North Africa and Middle East | 8975.103(4681.690,16486.216) | 6.878(3.603,12.589) | 6225.818(3727.043,10604.094) | 3.577(2.140,6.093) | -2.082(-3.051,-1.103) |
| South Asia | 22785.647(7682.214,44169.321) | 5.579(1.890,10.799) | 22813.335(11687.448,39448.360) | 4.772(2.428,8.313) | -0.628(-1.072,-0.183) |
| Central Sub-Saharan Africa | 11315.445(2860.954,21153.381) | 46.120(12.231,85.329) | 11613.280(5208.058,19000.079) | 21.128(9.501,34.520) | -2.131(-3.432,-0.813) |
| Eastern Sub-Saharan Africa | 74079.742(30243.457,117604.671) | 87.192(35.890,137.747) | 92764.428(51847.081,135124.872) | 55.826(31.253,81.212) | -1.337(-2.055,-0.614) |
| Southern Sub-Saharan Africa | 998.851(495.243,1738.346) | 5.184(2.579,9.006) | 2311.469(1069.272,4186.522) | 10.219(4.719,18.547) | 2.876(1.735,4.029) |
| Western Sub-Saharan Africa | 54395.925(22374.080,84880.799) | 65.155(27.397,100.756) | 100922.799(53347.220,141467.692) | 50.234(26.745,70.172) | -0.644(-1.560,0.280) |
| SDI |  |  |  |  |  |
| High-middle SDI | 12926.831(7361.479,19783.256) | 5.059(2.873,7.750) | 5284.474(3405.226,7161.679) | 2.377(1.526,3.232) | -2.772(-3.621,-1.915) |
| High SDI | 4432.044(3206.766,6266.088) | 2.522(1.824,3.565) | 2931.930(1518.210,3906.857) | 1.764(0.909,2.354) | -0.959(-2.078,0.173) |
| Low-middle SDI | 47294.164(23728.016,72057.482) | 10.660(5.375,16.212) | 61131.890(39370.734,85247.088) | 11.279(7.240,15.753) | 0.247(-0.481,0.981) |
| Low SDI | 116342.669(49978.890,185833.725) | 53.595(23.271,85.083) | 166557.293(92861.936,229405.880) | 38.826(21.703,53.415) | -0.901(-1.702,-0.094) |
| Middle SDI | 26002.861(15721.716,35694.952) | 4.828(2.917,6.631) | 19797.876(11841.091,26381.188) | 3.680(2.185,4.926) | -0.903(-1.639,-0.162) |
