# Supplementary material for: Global, regional, and national epidemiology of childhood Burkitt Lymphoma from 1990 to 2021: statistical analysis of incidence, mortality, and DALYs
Source: Front Public Health. 2025 Jul 16;13:1560003. doi: 10.3389/fpubh.2025.1560003 (PMC12307453; doi:10.3389/fpubh.2025.1560003)
Supplement: Supplementary file 9 [file Table_3.docx]

Table S3: The number of incident cases and incidence rates of Burkitt lymphoma in children in 204 countries and regions in 1990 and 2021, as well as the annual percentage change (EAPC) from 1990 to 2021.

| Location |  | Rate per 100,000 (95% UI) |  |  |  |
| --- | --- | --- | --- | --- | --- |
|  | 1990 |  | 2021 |  | 1990-2021 |
|  | Incident cases | Incident rate | Incident cases | Incident rate | EAPC |
| China | 160.989(64.673,281.711) | 0.054(0.022,0.095) | 172.532(88.205,318.129) | 0.071(0.036,0.130) | -0.554(-1.320,0.217) |
| Democratic People's Republic of Korea | 1.805(0.472,4.909) | 0.033(0.009,0.089) | 1.836(0.397,4.756) | 0.041(0.009,0.107) | 0.425(-0.419,1.276) |
| Taiwan (Province of China) | 3.570(2.010,6.318) | 0.068(0.038,0.120) | 3.417(1.117,6.572) | 0.120(0.039,0.232) | 2.633(1.687,3.587) |
| Cambodia | 1.413(0.194,5.026) | 0.032(0.005,0.114) | 1.109(0.313,3.095) | 0.023(0.007,0.065) | -1.670(-2.649,-0.681) |
| Indonesia | 9.107(2.121,22.103) | 0.014(0.003,0.035) | 13.039(5.624,25.068) | 0.021(0.009,0.040) | -0.631(-1.614,0.361) |
| Lao People's Democratic Republic | 0.439(0.047,1.714) | 0.025(0.003,0.098) | 0.554(0.149,1.548) | 0.026(0.007,0.073) | -2.488(-3.872,-1.084) |
| Malaysia | 1.348(0.387,3.806) | 0.022(0.006,0.062) | 2.036(0.492,5.460) | 0.028(0.007,0.075) | 0.516(-0.607,1.651) |
| Maldives | 0.056(0.011,0.184) | 0.057(0.011,0.184) | 0.070(0.019,0.163) | 0.074(0.020,0.173) | 1.299(0.105,2.508) |
| Myanmar | 5.278(0.614,20.259) | 0.039(0.004,0.149) | 3.695(1.015,9.898) | 0.025(0.007,0.068) | -2.147(-2.822,-1.467) |
| Philippines | 10.134(3.856,18.120) | 0.043(0.017,0.077) | 9.923(5.811,16.206) | 0.031(0.018,0.051) | -0.954(-1.939,0.040) |
| Sri Lanka | 2.236(0.677,5.540) | 0.043(0.013,0.106) | 3.543(0.952,7.983) | 0.072(0.019,0.162) | 2.119(1.198,3.048) |
| Thailand | 5.076(0.908,14.055) | 0.032(0.006,0.088) | 6.261(2.089,15.154) | 0.067(0.022,0.162) | 0.885(-0.430,2.217) |
| Timor-Leste | 0.082(0.008,0.310) | 0.025(0.003,0.095) | 0.081(0.020,0.236) | 0.017(0.004,0.049) | -2.088(-3.047,-1.120) |
| Viet Nam | 5.355(1.019,16.683) | 0.021(0.004,0.067) | 19.857(2.988,62.537) | 0.084(0.013,0.266) | 5.154(3.685,6.643) |
| Fiji | 0.073(0.018,0.187) | 0.028(0.007,0.072) | 0.180(0.050,0.466) | 0.071(0.020,0.184) | 3.233(3.003,3.464) |
| Kiribati | 0.001(0.000,0.003) | 0.003(0.001,0.011) | 0.001(0.000,0.004) | 0.003(0.001,0.011) | -0.768(-1.009,-0.527) |
| Marshall Islands | 0.002(0.001,0.006) | 0.011(0.003,0.030) | 0.004(0.001,0.013) | 0.028(0.007,0.080) | 2.542(2.209,2.875) |
| Micronesia (Federated States of) | 0.005(0.001,0.015) | 0.011(0.002,0.035) | 0.007(0.002,0.022) | 0.025(0.006,0.077) | 2.916(2.695,3.138) |
| Papua New Guinea | 0.389(0.072,1.404) | 0.024(0.004,0.086) | 1.577(0.283,4.924) | 0.042(0.007,0.130) | 1.901(1.558,2.245) |
| Samoa | 0.009(0.001,0.034) | 0.014(0.002,0.051) | 0.018(0.002,0.059) | 0.024(0.003,0.077) | 1.390(-1.669,4.543) |
| Solomon Islands | 0.018(0.004,0.056) | 0.012(0.003,0.038) | 0.057(0.014,0.173) | 0.023(0.006,0.071) | 2.050(1.726,2.375) |
| Tonga | 0.018(0.004,0.053) | 0.045(0.011,0.133) | 0.036(0.008,0.113) | 0.099(0.022,0.310) | 1.962(0.569,3.375) |
| Vanuatu | 0.006(0.001,0.018) | 0.009(0.002,0.028) | 0.019(0.004,0.058) | 0.017(0.004,0.053) | 2.158(1.847,2.471) |
| Armenia | 0.258(0.116,0.560) | 0.026(0.012,0.057) | 0.210(0.104,0.386) | 0.038(0.019,0.070) | 1.968(1.317,2.624) |
| Azerbaijan | 0.418(0.116,1.136) | 0.018(0.005,0.050) | 0.625(0.218,1.580) | 0.028(0.010,0.072) | 0.878(0.280,1.480) |
| Georgia | 2.784(0.677,5.419) | 0.216(0.053,0.421) | 0.534(0.227,0.955) | 0.077(0.033,0.138) | -3.754(-4.527,-2.974) |
| Kazakhstan | 2.440(0.920,5.677) | 0.050(0.019,0.117) | 2.237(0.878,4.413) | 0.044(0.017,0.087) | -0.550(-2.075,0.999) |
| Kyrgyzstan | 0.497(0.209,1.089) | 0.032(0.013,0.069) | 0.705(0.306,1.522) | 0.033(0.014,0.071) | -0.002(-1.132,1.142) |
| Mongolia | 0.278(0.041,1.036) | 0.032(0.005,0.120) | 0.627(0.184,1.391) | 0.062(0.018,0.138) | 0.095(-3.054,3.345) |
| Tajikistan | 0.039(0.007,0.134) | 0.002(0.000,0.006) | 0.048(0.013,0.137) | 0.001(0.000,0.004) | -1.732(-2.223,-1.238) |
| Turkmenistan | 0.203(0.065,0.589) | 0.014(0.005,0.040) | 0.256(0.109,0.576) | 0.018(0.008,0.040) | 0.532(0.094,0.972) |
| Uzbekistan | 0.972(0.378,2.361) | 0.012(0.005,0.029) | 1.617(0.718,3.820) | 0.017(0.008,0.041) | 1.239(0.402,2.082) |
| Albania | 0.049(0.015,0.133) | 0.005(0.001,0.013) | 0.088(0.016,0.282) | 0.021(0.004,0.068) | 5.740(4.650,6.842) |
| Bosnia and Herzegovina | 0.311(0.076,0.874) | 0.030(0.007,0.084) | 0.258(0.071,0.633) | 0.054(0.015,0.134) | 2.579(0.946,4.239) |
| Bulgaria | 0.453(0.186,0.937) | 0.028(0.011,0.057) | 0.385(0.160,0.707) | 0.041(0.017,0.076) | 1.271(0.407,2.142) |
| Croatia | 1.158(0.491,2.129) | 0.123(0.052,0.225) | 1.308(0.427,2.547) | 0.229(0.075,0.451) | 2.905(1.687,4.138) |
| Czechia | 1.891(1.056,3.290) | 0.090(0.051,0.157) | 2.210(0.560,5.270) | 0.136(0.035,0.323) | 1.161(-0.195,2.534) |
| Hungary | 1.364(0.655,2.565) | 0.068(0.033,0.126) | 1.677(0.435,3.458) | 0.128(0.034,0.264) | 2.218(0.895,3.559) |
| North Macedonia | 0.072(0.023,0.183) | 0.015(0.005,0.037) | 0.091(0.032,0.205) | 0.029(0.010,0.064) | 2.960(1.747,4.188) |
| Montenegro | 0.355(0.166,0.657) | 0.233(0.109,0.432) | 0.155(0.048,0.328) | 0.144(0.045,0.308) | -1.024(-2.266,0.234) |
| Poland | 3.781(1.083,7.758) | 0.041(0.012,0.085) | 6.371(2.291,9.960) | 0.114(0.041,0.179) | 3.312(2.090,4.549) |
| Romania | 4.975(1.729,12.698) | 0.095(0.033,0.245) | 5.624(2.570,9.619) | 0.196(0.090,0.336) | 3.039(1.829,4.262) |
| Serbia | 1.121(0.354,2.750) | 0.055(0.017,0.136) | 0.747(0.188,1.837) | 0.058(0.014,0.143) | 0.053(-1.056,1.174) |
| Slovakia | 0.529(0.170,1.220) | 0.041(0.013,0.096) | 0.937(0.279,2.433) | 0.117(0.035,0.303) | 3.454(2.142,4.783) |
| Slovenia | 0.293(0.156,0.546) | 0.073(0.039,0.137) | 0.208(0.054,0.409) | 0.070(0.018,0.137) | 0.885(-0.570,2.361) |
| Belarus | 2.054(1.019,3.731) | 0.090(0.045,0.163) | 2.111(0.628,5.044) | 0.136(0.040,0.328) | 3.187(1.890,4.501) |
| Estonia | 0.408(0.204,0.801) | 0.125(0.062,0.244) | 0.329(0.085,0.750) | 0.161(0.041,0.366) | 2.138(1.266,3.017) |
| Latvia | 0.497(0.218,0.986) | 0.093(0.041,0.185) | 0.274(0.067,0.607) | 0.096(0.024,0.214) | 0.977(-0.054,2.019) |
| Lithuania | 0.753(0.340,1.337) | 0.097(0.044,0.172) | 0.463(0.120,1.208) | 0.118(0.031,0.309) | 0.324(-0.766,1.425) |
| Republic of Moldova | 3.559(1.456,7.168) | 0.306(0.125,0.615) | 1.240(0.572,2.115) | 0.246(0.113,0.421) | 0.546(-0.466,1.569) |
| Russian Federation | 43.313(23.927,69.562) | 0.131(0.072,0.211) | 29.747(12.527,44.652) | 0.117(0.049,0.176) | 0.806(-0.149,1.770) |
| Ukraine | 9.204(3.758,22.181) | 0.085(0.035,0.205) | 6.351(2.004,15.077) | 0.099(0.031,0.237) | 0.510(-0.180,1.205) |
| Brunei Darussalam | 0.085(0.030,0.197) | 0.103(0.036,0.240) | 0.095(0.037,0.192) | 0.105(0.041,0.213) | 0.116(-0.485,0.720) |
| Japan | 24.745(9.525,45.854) | 0.110(0.043,0.205) | 29.548(11.858,45.710) | 0.196(0.078,0.306) | 1.861(0.732,3.002) |
| Republic of Korea | 4.830(1.303,12.782) | 0.043(0.012,0.114) | 5.264(1.610,12.166) | 0.085(0.026,0.197) | 2.089(0.548,3.654) |
| Singapore | 0.553(0.244,1.095) | 0.090(0.040,0.180) | 1.384(0.411,2.786) | 0.183(0.054,0.371) | 2.731(1.349,4.132) |
| Australia | 6.215(3.423,10.380) | 0.173(0.096,0.290) | 9.185(3.728,16.481) | 0.200(0.081,0.359) | 0.577(-0.646,1.815) |
| New Zealand | 1.544(0.894,2.496) | 0.208(0.120,0.336) | 2.160(1.178,3.413) | 0.226(0.123,0.357) | 0.049(-0.945,1.053) |
| Andorra | 0.036(0.009,0.092) | 0.401(0.104,1.029) | 0.051(0.017,0.106) | 0.508(0.166,1.064) | 1.150(-0.152,2.469) |
| Austria | 1.938(1.104,3.219) | 0.151(0.086,0.252) | 2.891(1.335,4.976) | 0.235(0.108,0.405) | 1.451(0.458,2.454) |
| Belgium | 2.765(1.302,4.863) | 0.162(0.077,0.286) | 6.033(2.404,10.757) | 0.327(0.130,0.584) | 1.914(0.848,2.991) |
| Cyprus | 0.342(0.109,0.767) | 0.181(0.058,0.408) | 0.552(0.221,1.097) | 0.268(0.107,0.533) | 2.775(1.614,3.949) |
| Denmark | 1.795(0.967,3.054) | 0.219(0.118,0.372) | 1.733(0.725,3.215) | 0.192(0.080,0.356) | -0.020(-1.304,1.280) |
| Finland | 1.434(0.724,2.647) | 0.156(0.079,0.287) | 1.387(0.464,2.568) | 0.164(0.054,0.305) | 0.406(-0.610,1.433) |
| France | 14.719(8.609,23.761) | 0.132(0.077,0.214) | 30.403(8.786,71.519) | 0.273(0.078,0.647) | 2.318(1.145,3.505) |
| Germany | 12.425(6.650,22.847) | 0.103(0.055,0.189) | 18.366(6.362,37.411) | 0.162(0.056,0.331) | 1.563(0.229,2.916) |
| Greece | 1.955(0.634,3.655) | 0.097(0.032,0.182) | 1.968(1.011,3.048) | 0.146(0.076,0.226) | 1.273(0.078,2.482) |
| Iceland | 0.097(0.048,0.172) | 0.161(0.079,0.287) | 0.080(0.036,0.156) | 0.123(0.055,0.240) | -0.053(-1.518,1.433) |
| Ireland | 1.034(0.534,1.794) | 0.108(0.056,0.188) | 2.312(0.840,4.600) | 0.237(0.086,0.474) | 2.357(1.041,3.689) |
| Israel | 4.223(2.006,7.621) | 0.293(0.139,0.529) | 11.449(5.742,18.636) | 0.466(0.234,0.758) | 1.793(0.861,2.734) |
| Italy | 15.075(9.243,25.631) | 0.169(0.103,0.289) | 23.311(7.852,47.947) | 0.310(0.104,0.641) | 2.230(1.162,3.309) |
| Luxembourg | 0.111(0.040,0.194) | 0.179(0.065,0.314) | 0.334(0.155,0.542) | 0.346(0.161,0.563) | 1.319(0.287,2.362) |
| Malta | 0.108(0.044,0.194) | 0.130(0.053,0.234) | 0.242(0.110,0.515) | 0.402(0.183,0.855) | 2.876(2.120,3.638) |
| Netherlands | 8.542(4.727,13.910) | 0.336(0.186,0.547) | 11.046(4.613,18.631) | 0.430(0.179,0.725) | 1.218(0.127,2.322) |
| Norway | 0.717(0.418,1.296) | 0.097(0.056,0.175) | 0.908(0.381,1.572) | 0.101(0.042,0.176) | 0.510(-0.671,1.705) |
| Portugal | 2.821(1.400,6.047) | 0.137(0.068,0.294) | 3.669(1.305,7.060) | 0.281(0.101,0.540) | 2.046(0.834,3.273) |
| Spain | 15.199(8.004,26.219) | 0.196(0.103,0.339) | 21.671(7.343,44.820) | 0.339(0.114,0.705) | 1.778(0.624,2.946) |
| Sweden | 1.193(0.263,2.473) | 0.084(0.018,0.174) | 2.775(1.280,4.878) | 0.159(0.073,0.280) | 2.535(1.532,3.549) |
| Switzerland | 1.921(0.934,3.508) | 0.178(0.086,0.325) | 2.155(0.827,4.594) | 0.170(0.065,0.364) | -0.181(-1.284,0.934) |
| United Kingdom | 10.110(2.472,19.178) | 0.099(0.024,0.188) | 17.593(7.903,28.812) | 0.155(0.069,0.253) | 1.972(0.817,3.140) |
| Argentina | 15.148(8.115,25.799) | 0.159(0.085,0.271) | 23.385(13.019,38.396) | 0.238(0.133,0.393) | 1.577(0.691,2.471) |
| Chile | 5.542(2.350,9.674) | 0.150(0.064,0.262) | 9.875(4.979,15.633) | 0.281(0.141,0.446) | 2.768(1.852,3.693) |
| Uruguay | 1.941(0.995,3.229) | 0.253(0.130,0.421) | 2.346(1.249,3.810) | 0.367(0.195,0.600) | 1.104(0.151,2.066) |
| Canada | 11.425(6.111,19.539) | 0.210(0.112,0.359) | 13.804(5.732,26.321) | 0.228(0.095,0.435) | -0.009(-1.490,1.495) |
| United States of America | 111.118(73.886,156.513) | 0.214(0.142,0.301) | 108.079(66.033,156.525) | 0.188(0.114,0.272) | 0.226(-1.104,1.574) |
| Antigua and Barbuda | 0.016(0.008,0.028) | 0.096(0.047,0.161) | 0.025(0.014,0.042) | 0.156(0.086,0.263) | 1.552(0.605,2.507) |
| Bahamas | 0.090(0.049,0.146) | 0.119(0.065,0.194) | 0.118(0.064,0.191) | 0.148(0.079,0.240) | 0.499(0.018,0.981) |
| Barbados | 0.234(0.134,0.364) | 0.394(0.225,0.613) | 0.189(0.105,0.300) | 0.409(0.224,0.654) | 0.688(-0.464,1.852) |
| Belize | 0.048(0.023,0.103) | 0.063(0.030,0.133) | 0.081(0.045,0.127) | 0.068(0.038,0.108) | 1.012(0.755,1.270) |
| Cuba | 9.944(4.659,17.431) | 0.429(0.201,0.751) | 4.626(2.132,8.916) | 0.271(0.124,0.525) | 0.832(-0.356,2.034) |
| Dominica | 0.018(0.005,0.045) | 0.078(0.023,0.194) | 0.016(0.005,0.039) | 0.120(0.038,0.297) | 1.749(1.010,2.493) |
| Dominican Republic | 1.209(0.347,3.255) | 0.048(0.014,0.129) | 2.703(0.839,6.895) | 0.099(0.031,0.252) | 2.300(1.572,3.033) |
| Grenada | 0.056(0.028,0.103) | 0.180(0.090,0.328) | 0.052(0.025,0.093) | 0.251(0.122,0.446) | 1.128(0.382,1.879) |
| Guyana | 0.236(0.101,0.436) | 0.088(0.038,0.163) | 0.094(0.039,0.206) | 0.047(0.019,0.103) | 0.047(-0.517,0.615) |
| Haiti | 6.574(0.841,24.415) | 0.252(0.034,0.925) | 8.206(1.770,25.634) | 0.202(0.044,0.628) | -0.421(-0.746,-0.094) |
| Jamaica | 0.937(0.410,1.925) | 0.120(0.052,0.247) | 0.623(0.290,1.289) | 0.111(0.051,0.230) | 0.019(-0.625,0.666) |
| Saint Lucia | 0.043(0.022,0.078) | 0.090(0.045,0.163) | 0.047(0.024,0.077) | 0.163(0.085,0.271) | 1.917(1.228,2.611) |
| Saint Vincent and the Grenadines | 0.037(0.009,0.078) | 0.096(0.024,0.202) | 0.033(0.018,0.057) | 0.136(0.073,0.232) | 1.172(0.824,1.521) |
| Suriname | 0.046(0.016,0.108) | 0.037(0.013,0.088) | 0.086(0.026,0.226) | 0.063(0.019,0.165) | 2.529(2.064,2.995) |
| Trinidad and Tobago | 0.484(0.277,0.862) | 0.124(0.071,0.221) | 0.404(0.214,0.667) | 0.153(0.081,0.253) | 1.404(0.435,2.382) |
| Bolivia (Plurinational State of) | 4.420(1.375,10.698) | 0.177(0.055,0.425) | 4.358(1.339,9.880) | 0.133(0.041,0.303) | -1.852(-2.566,-1.132) |
| Ecuador | 2.699(1.532,4.475) | 0.075(0.043,0.124) | 7.147(2.627,13.377) | 0.149(0.055,0.278) | 2.574(1.641,3.516) |
| Peru | 10.799(4.451,21.903) | 0.140(0.058,0.284) | 20.370(7.112,43.144) | 0.228(0.080,0.483) | 1.925(1.034,2.823) |
| Colombia | 14.064(7.855,23.517) | 0.130(0.073,0.218) | 27.776(12.306,54.080) | 0.278(0.123,0.543) | 3.274(2.346,4.210) |
| Costa Rica | 2.080(0.893,3.566) | 0.198(0.085,0.339) | 2.804(0.969,6.206) | 0.288(0.099,0.636) | 1.134(0.288,1.987) |
| El Salvador | 1.041(0.500,1.905) | 0.052(0.025,0.096) | 1.533(0.687,2.756) | 0.089(0.040,0.160) | 1.820(1.375,2.267) |
| Guatemala | 3.737(1.961,7.826) | 0.098(0.052,0.204) | 3.330(1.753,5.247) | 0.071(0.037,0.112) | -0.484(-1.093,0.129) |
| Honduras | 1.094(0.365,2.491) | 0.053(0.018,0.120) | 1.085(0.385,2.323) | 0.035(0.013,0.076) | -1.751(-1.986,-1.516) |
| Mexico | 24.563(14.005,43.150) | 0.079(0.045,0.139) | 36.955(18.286,56.127) | 0.121(0.060,0.185) | 1.677(1.028,2.331) |
| Nicaragua | 1.576(0.647,3.136) | 0.092(0.038,0.182) | 2.114(1.032,3.810) | 0.113(0.055,0.204) | 0.920(0.023,1.825) |
| Panama | 1.368(0.782,2.244) | 0.176(0.100,0.288) | 3.755(2.198,5.655) | 0.344(0.201,0.518) | 1.883(1.318,2.451) |
| Venezuela (Bolivarian Republic of) | 6.989(2.951,12.754) | 0.106(0.045,0.194) | 21.460(12.372,33.068) | 0.347(0.200,0.536) | 3.514(2.587,4.450) |
| Brazil | 62.999(41.608,97.400) | 0.129(0.085,0.200) | 82.016(40.829,119.162) | 0.182(0.090,0.264) | 1.378(0.578,2.185) |
| Paraguay | 1.099(0.378,2.485) | 0.070(0.024,0.157) | 2.078(0.634,4.490) | 0.110(0.034,0.239) | 1.534(0.876,2.197) |
| Algeria | 10.798(2.424,28.325) | 0.106(0.024,0.279) | 23.578(6.709,59.714) | 0.188(0.054,0.477) | 2.308(1.178,3.450) |
| Bahrain | 0.054(0.014,0.139) | 0.036(0.009,0.093) | 0.138(0.030,0.349) | 0.048(0.011,0.122) | 0.992(-0.459,2.465) |
| Egypt | 7.917(1.245,28.517) | 0.039(0.006,0.140) | 11.312(1.051,47.839) | 0.033(0.003,0.139) | -0.380(-1.431,0.681) |
| Iran (Islamic Republic of) | 9.613(3.892,22.141) | 0.039(0.016,0.090) | 15.375(5.734,30.743) | 0.077(0.029,0.155) | 2.321(1.169,3.486) |
| Iraq | 11.809(1.674,35.814) | 0.157(0.022,0.475) | 13.818(3.971,34.247) | 0.107(0.031,0.266) | -0.872(-1.783,0.047) |
| Jordan | 1.704(0.521,4.209) | 0.113(0.035,0.280) | 6.680(2.351,14.689) | 0.191(0.067,0.420) | 1.393(0.428,2.368) |
| Kuwait | 1.055(0.521,2.031) | 0.205(0.102,0.394) | 1.085(0.479,2.130) | 0.132(0.058,0.260) | -0.385(-1.046,0.280) |
| Lebanon | 1.074(0.316,2.554) | 0.113(0.033,0.267) | 2.282(0.707,5.369) | 0.185(0.057,0.435) | 1.570(-0.024,3.189) |
| Libya | 2.122(0.544,5.774) | 0.126(0.032,0.343) | 4.572(1.275,11.974) | 0.326(0.087,0.865) | 3.740(2.523,4.971) |
| Morocco | 5.948(1.715,15.919) | 0.066(0.019,0.176) | 7.283(2.272,17.010) | 0.079(0.024,0.184) | 1.043(0.933,1.153) |
| Palestine | 0.519(0.130,1.350) | 0.060(0.015,0.154) | 1.435(0.381,3.302) | 0.081(0.021,0.186) | 1.548(-0.817,3.970) |
| Oman | 0.578(0.161,1.596) | 0.076(0.021,0.208) | 1.966(0.596,4.480) | 0.170(0.052,0.388) | 2.786(1.472,4.117) |
| Qatar | 0.035(0.007,0.097) | 0.032(0.006,0.088) | 0.304(0.070,0.757) | 0.067(0.016,0.167) | 2.930(1.938,3.932) |
| Saudi Arabia | 4.319(1.534,9.995) | 0.071(0.025,0.163) | 8.930(1.945,20.056) | 0.123(0.027,0.276) | 2.112(1.160,3.074) |
| Syrian Arab Republic | 1.129(0.390,2.775) | 0.021(0.007,0.050) | 2.343(0.571,5.096) | 0.068(0.016,0.148) | 3.328(2.828,3.830) |
| Tunisia | 4.236(1.112,11.644) | 0.145(0.038,0.399) | 5.212(1.477,12.667) | 0.196(0.055,0.478) | 1.139(0.055,2.234) |
| T眉rkiye | 47.169(16.241,102.743) | 0.246(0.084,0.535) | 68.949(31.130,121.829) | 0.380(0.171,0.674) | 1.590(0.386,2.809) |
| United Arab Emirates | 0.444(0.132,1.050) | 0.083(0.025,0.195) | 0.954(0.306,2.158) | 0.075(0.024,0.169) | -0.100(-0.796,0.600) |
| Yemen | 3.651(0.559,12.357) | 0.056(0.009,0.187) | 6.705(1.193,18.627) | 0.052(0.009,0.143) | -0.035(-1.399,1.347) |
| Afghanistan | 4.379(0.727,15.302) | 0.112(0.019,0.387) | 8.817(1.723,25.660) | 0.068(0.013,0.199) | -1.169(-2.284,-0.042) |
| Bangladesh | 24.803(5.087,76.789) | 0.054(0.011,0.167) | 30.127(7.165,77.429) | 0.069(0.016,0.176) | 0.074(-0.657,0.810) |
| Bhutan | 0.131(0.023,0.476) | 0.054(0.010,0.197) | 0.117(0.022,0.328) | 0.067(0.013,0.188) | -0.481(-1.457,0.505) |
| India | 178.870(54.809,369.930) | 0.058(0.018,0.120) | 126.710(73.578,205.015) | 0.036(0.021,0.058) | -1.918(-2.620,-1.211) |
| Nepal | 3.401(0.809,10.292) | 0.044(0.011,0.131) | 3.881(0.778,10.601) | 0.045(0.009,0.123) | -0.766(-1.800,0.279) |
| Pakistan | 67.583(22.270,146.597) | 0.148(0.049,0.320) | 171.856(62.090,378.842) | 0.215(0.078,0.473) | 1.329(1.144,1.514) |
| Angola | 32.224(6.408,67.870) | 0.705(0.148,1.472) | 41.364(15.785,76.074) | 0.289(0.111,0.530) | -2.752(-4.140,-1.344) |
| Central African Republic | 6.626(1.688,13.269) | 0.568(0.151,1.127) | 9.390(3.237,17.821) | 0.441(0.153,0.834) | -0.662(-2.028,0.723) |
| Congo | 4.153(1.257,7.870) | 0.421(0.129,0.796) | 4.627(2.108,7.993) | 0.256(0.116,0.443) | -1.579(-2.830,-0.311) |
| Democratic Republic of the Congo | 86.998(20.947,172.630) | 0.508(0.128,0.997) | 81.103(34.007,145.273) | 0.228(0.096,0.408) | -2.034(-3.280,-0.771) |
| Equatorial Guinea | 0.990(0.262,1.972) | 0.527(0.146,1.038) | 2.065(0.731,4.853) | 0.373(0.131,0.875) | -1.803(-2.999,-0.593) |
| Gabon | 1.288(0.471,2.344) | 0.338(0.125,0.613) | 2.053(0.846,3.993) | 0.342(0.140,0.667) | 0.226(-1.085,1.554) |
| Burundi | 34.567(11.717,67.764) | 1.401(0.481,2.730) | 31.758(14.143,59.759) | 0.586(0.262,1.099) | -2.597(-3.242,-1.948) |
| Comoros | 1.941(0.735,3.588) | 0.988(0.375,1.818) | 1.593(0.762,2.854) | 0.709(0.338,1.271) | -1.414(-1.882,-0.944) |
| Djibouti | 1.225(0.491,2.210) | 0.766(0.309,1.375) | 2.566(1.165,4.649) | 0.665(0.302,1.204) | -0.509(-1.105,0.091) |
| Eritrea | 14.096(5.188,25.749) | 0.948(0.352,1.726) | 17.669(8.205,31.962) | 0.749(0.349,1.353) | -0.869(-1.484,-0.251) |
| Ethiopia | 248.288(70.051,514.918) | 1.094(0.312,2.259) | 220.651(107.157,373.612) | 0.538(0.262,0.909) | -2.600(-3.712,-1.476) |
| Kenya | 31.639(14.503,48.339) | 0.304(0.141,0.461) | 47.655(27.583,68.702) | 0.269(0.155,0.390) | 0.570(-0.474,1.624) |
| Madagascar | 44.057(17.912,77.546) | 0.871(0.358,1.522) | 59.224(29.980,101.124) | 0.539(0.273,0.920) | -1.092(-1.679,-0.501) |
| Malawi | 96.737(35.082,176.646) | 2.269(0.834,4.103) | 106.092(43.640,213.352) | 1.401(0.575,2.827) | -1.352(-1.898,-0.803) |
| Mauritius | 0.050(0.030,0.080) | 0.016(0.010,0.026) | 0.049(0.024,0.084) | 0.025(0.012,0.043) | 0.838(0.136,1.544) |
| Mozambique | 10.971(3.711,26.002) | 0.188(0.064,0.445) | 11.806(4.215,31.178) | 0.089(0.032,0.234) | -2.074(-2.875,-1.267) |
| Rwanda | 47.965(17.018,89.758) | 1.510(0.541,2.810) | 31.125(15.469,56.564) | 0.672(0.334,1.221) | -3.233(-3.750,-2.714) |
| Seychelles | 0.000(0.000,0.000) | 0.000(0.000,0.000) | 0.000(0.000,0.000) | 0.000(0.000,0.000) | 2.965(1.971,3.970) |
| Somalia | 30.054(9.902,61.089) | 0.822(0.275,1.660) | 56.836(21.713,108.323) | 0.602(0.232,1.139) | -1.054(-1.870,-0.230) |
| United Republic of Tanzania | 130.988(49.120,236.183) | 1.170(0.442,2.093) | 176.808(86.370,317.705) | 0.778(0.381,1.394) | -0.903(-1.576,-0.225) |
| Uganda | 118.417(58.085,199.874) | 1.483(0.742,2.483) | 270.581(135.547,487.943) | 1.468(0.738,2.638) | -0.079(-0.728,0.575) |
| Zambia | 40.238(15.058,72.831) | 1.145(0.436,2.054) | 52.519(23.517,98.752) | 0.678(0.304,1.276) | -1.874(-2.633,-1.108) |
| Botswana | 0.420(0.139,0.929) | 0.077(0.025,0.169) | 1.110(0.327,2.427) | 0.171(0.050,0.375) | 3.011(1.964,4.068) |
| Lesotho | 0.439(0.156,0.971) | 0.069(0.025,0.153) | 0.784(0.257,1.725) | 0.131(0.043,0.289) | 2.771(1.536,4.020) |
| Namibia | 0.687(0.240,1.598) | 0.124(0.044,0.287) | 1.900(0.683,4.101) | 0.246(0.089,0.532) | 2.775(1.675,3.887) |
| South Africa | 5.203(2.256,9.779) | 0.041(0.018,0.077) | 10.376(5.954,17.053) | 0.072(0.041,0.119) | 1.628(0.881,2.381) |
| Eswatini | 0.362(0.109,0.896) | 0.101(0.031,0.248) | 0.558(0.169,1.279) | 0.144(0.044,0.331) | 1.615(0.324,2.923) |
| Zimbabwe | 5.250(1.802,11.654) | 0.118(0.040,0.261) | 17.359(5.228,39.012) | 0.295(0.089,0.664) | 5.062(3.337,6.817) |
| Benin | 15.532(5.244,29.967) | 0.668(0.231,1.277) | 37.193(15.916,66.914) | 0.652(0.282,1.167) | 0.047(-1.032,1.138) |
| Burkina Faso | 32.078(10.950,61.917) | 0.713(0.248,1.367) | 64.767(28.793,114.661) | 0.669(0.301,1.177) | 0.266(-0.768,1.311) |
| Cameroon | 31.314(11.977,58.934) | 0.682(0.268,1.270) | 93.353(41.996,161.162) | 0.742(0.334,1.280) | 0.639(-0.202,1.487) |
| Cabo Verde | 0.196(0.082,0.371) | 0.134(0.056,0.252) | 0.951(0.332,1.803) | 0.687(0.241,1.303) | 4.408(3.594,5.228) |
| Chad | 15.414(5.369,30.197) | 0.559(0.200,1.080) | 55.695(22.462,100.052) | 0.664(0.271,1.184) | 0.936(-0.191,2.075) |
| C么te d'Ivoire | 36.174(14.196,66.044) | 0.676(0.270,1.222) | 68.877(29.625,127.003) | 0.641(0.276,1.177) | 0.260(-0.280,0.803) |
| Gambia | 2.609(1.000,5.012) | 0.601(0.236,1.142) | 5.005(2.086,9.699) | 0.542(0.227,1.049) | -0.257(-1.135,0.629) |
| Ghana | 73.720(19.607,149.197) | 1.174(0.313,2.369) | 55.851(27.333,104.620) | 0.465(0.227,0.869) | -3.979(-5.140,-2.805) |
| Guinea | 19.103(6.469,37.693) | 0.719(0.255,1.397) | 27.382(11.953,55.078) | 0.483(0.213,0.967) | -0.681(-1.272,-0.087) |
| Guinea-Bissau | 3.819(1.323,7.481) | 0.844(0.298,1.643) | 4.449(2.039,8.264) | 0.535(0.246,0.995) | -1.021(-2.041,0.009) |
| Liberia | 9.995(3.229,20.135) | 0.919(0.306,1.821) | 12.435(5.313,21.961) | 0.611(0.261,1.078) | -1.351(-2.157,-0.538) |
| Mali | 20.985(7.957,39.844) | 0.529(0.208,0.990) | 37.794(17.863,70.914) | 0.350(0.168,0.648) | -0.936(-1.770,-0.095) |
| Mauritania | 4.169(1.720,7.703) | 0.485(0.203,0.889) | 9.163(4.475,16.688) | 0.528(0.258,0.961) | -0.058(-0.754,0.642) |
| Niger | 35.265(10.233,75.808) | 0.905(0.271,1.919) | 60.469(23.565,114.333) | 0.506(0.200,0.949) | -1.752(-2.825,-0.667) |
| Nigeria | 292.579(126.441,502.117) | 0.791(0.353,1.344) | 726.175(345.609,1107.287) | 0.766(0.367,1.165) | 0.098(-0.821,1.026) |
| Sao Tome and Principe | 0.408(0.154,0.752) | 0.769(0.291,1.418) | 0.303(0.139,0.598) | 0.411(0.187,0.812) | -1.596(-2.311,-0.876) |
| Senegal | 24.619(9.129,45.933) | 0.709(0.269,1.312) | 27.176(13.335,49.068) | 0.460(0.226,0.829) | -1.195(-2.030,-0.353) |
| Sierra Leone | 15.126(4.780,30.509) | 0.868(0.286,1.720) | 22.076(10.587,38.890) | 0.666(0.321,1.169) | -0.610(-1.551,0.339) |
| Togo | 9.781(3.907,17.876) | 0.588(0.238,1.070) | 15.455(7.440,28.316) | 0.498(0.240,0.912) | -0.203(-1.132,0.735) |
| American Samoa | 0.002(0.000,0.005) | 0.009(0.002,0.027) | 0.004(0.001,0.011) | 0.030(0.007,0.084) | 3.925(2.626,5.240) |
| Bermuda | 0.027(0.009,0.053) | 0.246(0.083,0.469) | 0.035(0.012,0.072) | 0.451(0.151,0.930) | 2.260(1.927,2.594) |
| Cook Islands | 0.001(0.000,0.003) | 0.016(0.004,0.044) | 0.002(0.000,0.005) | 0.047(0.005,0.142) | 2.467(2.240,2.693) |
| Greenland | 0.022(0.003,0.058) | 0.170(0.022,0.445) | 0.007(0.002,0.020) | 0.068(0.016,0.182) | -1.563(-2.810,-0.300) |
| Guam | 0.015(0.004,0.036) | 0.037(0.011,0.089) | 0.037(0.018,0.062) | 0.107(0.053,0.183) | 5.779(5.109,6.454) |
| Monaco | 0.002(0.000,0.006) | 0.058(0.009,0.176) | 0.005(0.001,0.013) | 0.105(0.022,0.281) | 1.713(1.127,2.303) |
| Nauru | 0.001(0.000,0.003) | 0.024(0.006,0.066) | 0.002(0.000,0.006) | 0.052(0.011,0.153) | 2.414(2.223,2.605) |
| Niue | 0.000(0.000,0.000) | 0.023(0.005,0.069) | 0.000(0.000,0.001) | 0.102(0.019,0.364) | 4.305(3.587,5.028) |
| Northern Mariana Islands | 0.001(0.000,0.004) | 0.012(0.002,0.038) | 0.003(0.001,0.008) | 0.030(0.008,0.078) | 4.245(3.638,4.856) |
| Palau | 0.001(0.000,0.003) | 0.025(0.007,0.065) | 0.001(0.000,0.003) | 0.043(0.013,0.100) | 1.929(0.554,3.323) |
| Puerto Rico | 2.443(1.009,4.176) | 0.261(0.108,0.447) | 1.218(0.509,2.032) | 0.277(0.115,0.463) | 0.993(0.171,1.821) |
| Saint Kitts and Nevis | 0.006(0.003,0.011) | 0.044(0.022,0.082) | 0.008(0.004,0.018) | 0.089(0.043,0.188) | 3.388(2.579,4.204) |
| San Marino | 0.014(0.003,0.038) | 0.373(0.081,0.988) | 0.019(0.004,0.045) | 0.435(0.093,1.027) | 0.618(-1.264,2.536) |
| Tokelau | 0.000(0.000,0.000) | 0.016(0.004,0.045) | 0.000(0.000,0.001) | 0.064(0.012,0.224) | 3.199(2.597,3.804) |
| Tuvalu | 0.001(0.000,0.003) | 0.029(0.006,0.090) | 0.001(0.000,0.003) | 0.031(0.008,0.081) | 0.378(0.209,0.548) |
| United States Virgin Islands | 0.018(0.005,0.044) | 0.061(0.016,0.148) | 0.007(0.001,0.018) | 0.053(0.011,0.141) | 0.630(-0.124,1.390) |
| South Sudan | 24.781(9.288,46.658) | 1.004(0.380,1.882) | 48.985(20.160,90.862) | 1.247(0.515,2.309) | 0.667(0.015,1.323) |
| Sudan | 6.702(1.022,28.066) | 0.082(0.013,0.339) | 14.554(2.641,39.657) | 0.093(0.017,0.254) | 0.435(-0.557,1.436) |
