# Supplementary material for: Global, regional, and national epidemiology of childhood Burkitt Lymphoma from 1990 to 2021: statistical analysis of incidence, mortality, and DALYs
Source: Front Public Health. 2025 Jul 16;13:1560003. doi: 10.3389/fpubh.2025.1560003 (PMC12307453; doi:10.3389/fpubh.2025.1560003)
Supplement: Supplementary file 10 [file Table_4.docx]

Table S4: The number of deaths and mortality rates of Burkitt lymphoma in children in 204 countries and regions in 1990 and 2021, as well as the annual percentage change (EAPC) from 1990 to 2021.

| Location |  | Rate per 100,000 (95% UI) |  |  |  |
| --- | --- | --- | --- | --- | --- |
|  | 1990 |  | 2021 |  | 1990-2021 |
|  | Number of deaths | Mortality rate | Number of deaths | Mortality rate | EAPC |
| China | 131.975(52.914,223.793) | 0.045(0.018,0.076) | 30.500(15.972,55.373) | 0.013(0.007,0.023) | -6.021(-6.808,-5.227) |
| Democratic People's Republic of Korea | 1.559(0.411,4.247) | 0.029(0.008,0.077) | 0.856(0.184,2.193) | 0.019(0.004,0.050) | -1.748(-2.575,-0.914) |
| Taiwan (Province of China) | 1.149(0.671,1.993) | 0.022(0.013,0.038) | 0.472(0.151,0.910) | 0.017(0.005,0.032) | -0.198(-1.082,0.695) |
| Cambodia | 1.403(0.197,5.069) | 0.032(0.005,0.115) | 0.935(0.274,2.457) | 0.020(0.006,0.052) | -2.066(-3.042,-1.080) |
| Indonesia | 9.029(2.122,21.461) | 0.014(0.003,0.034) | 9.699(4.360,17.986) | 0.015(0.007,0.029) | -1.498(-2.473,-0.514) |
| Lao People's Democratic Republic | 0.442(0.046,1.742) | 0.026(0.003,0.100) | 0.539(0.146,1.491) | 0.025(0.007,0.070) | -2.509(-3.903,-1.096) |
| Malaysia | 1.188(0.360,3.264) | 0.020(0.006,0.054) | 0.771(0.196,2.046) | 0.011(0.003,0.028) | -2.362(-3.472,-1.240) |
| Maldives | 0.056(0.011,0.176) | 0.057(0.011,0.176) | 0.021(0.006,0.048) | 0.022(0.006,0.051) | -3.243(-4.386,-2.086) |
| Myanmar | 5.265(0.586,21.203) | 0.039(0.004,0.157) | 3.177(0.881,8.299) | 0.022(0.006,0.057) | -2.544(-3.214,-1.871) |
| Philippines | 9.109(3.445,16.160) | 0.039(0.015,0.069) | 7.244(4.405,11.314) | 0.023(0.014,0.036) | -1.470(-2.457,-0.472) |
| Sri Lanka | 1.820(0.549,4.313) | 0.035(0.011,0.083) | 1.048(0.291,2.400) | 0.021(0.006,0.049) | -1.461(-2.351,-0.563) |
| Thailand | 3.545(0.671,9.371) | 0.022(0.004,0.059) | 1.674(0.578,4.128) | 0.018(0.006,0.044) | -2.365(-3.641,-1.072) |
| Timor-Leste | 0.079(0.008,0.290) | 0.025(0.003,0.090) | 0.076(0.019,0.231) | 0.016(0.004,0.048) | -2.162(-3.116,-1.199) |
| Viet Nam | 4.969(1.025,15.245) | 0.020(0.004,0.061) | 7.107(1.106,22.289) | 0.030(0.005,0.095) | 1.745(0.323,3.186) |
| Fiji | 0.072(0.017,0.185) | 0.028(0.007,0.071) | 0.149(0.043,0.379) | 0.060(0.017,0.151) | 2.796(2.542,3.050) |
| Kiribati | 0.001(0.000,0.003) | 0.003(0.001,0.011) | 0.001(0.000,0.004) | 0.003(0.001,0.011) | -0.769(-1.005,-0.532) |
| Marshall Islands | 0.002(0.001,0.006) | 0.011(0.003,0.030) | 0.004(0.001,0.013) | 0.028(0.007,0.080) | 2.534(2.195,2.874) |
| Micronesia (Federated States of) | 0.005(0.001,0.015) | 0.011(0.002,0.035) | 0.007(0.002,0.020) | 0.023(0.006,0.071) | 2.687(2.459,2.914) |
| Papua New Guinea | 0.383(0.073,1.369) | 0.024(0.004,0.084) | 1.576(0.286,4.970) | 0.042(0.008,0.132) | 1.963(1.612,2.316) |
| Samoa | 0.009(0.001,0.032) | 0.014(0.002,0.049) | 0.012(0.001,0.037) | 0.016(0.002,0.049) | 0.191(-2.834,3.310) |
| Solomon Islands | 0.018(0.004,0.057) | 0.012(0.003,0.039) | 0.057(0.014,0.176) | 0.023(0.006,0.073) | 2.052(1.734,2.372) |
| Tonga | 0.016(0.004,0.043) | 0.040(0.010,0.110) | 0.022(0.005,0.065) | 0.059(0.014,0.178) | 0.818(-0.558,2.213) |
| Vanuatu | 0.006(0.001,0.018) | 0.009(0.002,0.028) | 0.019(0.004,0.056) | 0.017(0.004,0.052) | 2.160(1.851,2.470) |
| Armenia | 0.142(0.065,0.307) | 0.015(0.007,0.031) | 0.059(0.030,0.105) | 0.011(0.005,0.019) | -0.516(-1.144,0.115) |
| Azerbaijan | 0.349(0.106,0.956) | 0.015(0.005,0.042) | 0.270(0.095,0.691) | 0.012(0.004,0.032) | -1.600(-2.222,-0.975) |
| Georgia | 1.350(0.337,2.575) | 0.105(0.026,0.201) | 0.197(0.085,0.352) | 0.029(0.012,0.051) | -4.303(-5.063,-3.538) |
| Kazakhstan | 1.633(0.634,3.604) | 0.034(0.013,0.075) | 0.731(0.287,1.459) | 0.015(0.006,0.029) | -3.475(-4.909,-2.020) |
| Kyrgyzstan | 0.398(0.173,0.834) | 0.026(0.011,0.053) | 0.296(0.133,0.635) | 0.014(0.006,0.030) | -2.270(-3.343,-1.185) |
| Mongolia | 0.274(0.041,1.019) | 0.032(0.005,0.119) | 0.367(0.109,0.800) | 0.037(0.011,0.080) | -1.758(-4.851,1.435) |
| Tajikistan | 0.037(0.007,0.133) | 0.002(0.000,0.006) | 0.035(0.011,0.096) | 0.001(0.000,0.003) | -2.510(-2.980,-2.037) |
| Turkmenistan | 0.179(0.060,0.508) | 0.012(0.004,0.035) | 0.135(0.058,0.293) | 0.010(0.004,0.021) | -1.319(-1.751,-0.885) |
| Uzbekistan | 0.763(0.318,1.870) | 0.009(0.004,0.023) | 0.858(0.400,2.026) | 0.009(0.004,0.022) | -0.368(-1.191,0.462) |
| Albania | 0.030(0.010,0.080) | 0.003(0.001,0.008) | 0.018(0.004,0.059) | 0.004(0.001,0.014) | 1.616(0.702,2.537) |
| Bosnia and Herzegovina | 0.154(0.037,0.431) | 0.015(0.004,0.042) | 0.053(0.015,0.130) | 0.011(0.003,0.028) | -0.910(-2.459,0.664) |
| Bulgaria | 0.192(0.080,0.401) | 0.012(0.005,0.025) | 0.099(0.040,0.181) | 0.011(0.004,0.019) | -0.460(-1.326,0.414) |
| Croatia | 0.301(0.128,0.547) | 0.032(0.014,0.058) | 0.155(0.051,0.296) | 0.027(0.009,0.053) | 0.116(-1.063,1.309) |
| Czechia | 0.603(0.346,1.018) | 0.029(0.017,0.049) | 0.270(0.071,0.625) | 0.017(0.004,0.039) | -1.701(-2.971,-0.414) |
| Hungary | 0.535(0.255,0.950) | 0.027(0.013,0.047) | 0.268(0.071,0.560) | 0.021(0.005,0.043) | -0.560(-1.810,0.705) |
| North Macedonia | 0.038(0.012,0.099) | 0.008(0.003,0.020) | 0.021(0.007,0.046) | 0.006(0.002,0.015) | -0.324(-1.466,0.830) |
| Montenegro | 0.084(0.039,0.151) | 0.055(0.025,0.100) | 0.024(0.008,0.051) | 0.023(0.007,0.048) | -2.845(-4.071,-1.604) |
| Poland | 1.782(0.533,3.657) | 0.020(0.006,0.040) | 1.110(0.403,1.674) | 0.020(0.007,0.030) | -0.035(-1.171,1.114) |
| Romania | 2.827(1.041,6.879) | 0.054(0.020,0.134) | 1.149(0.551,1.954) | 0.040(0.019,0.069) | -0.531(-1.681,0.632) |
| Serbia | 0.498(0.156,1.226) | 0.025(0.008,0.061) | 0.132(0.035,0.327) | 0.010(0.003,0.026) | -3.258(-4.333,-2.170) |
| Slovakia | 0.198(0.063,0.456) | 0.016(0.005,0.036) | 0.157(0.046,0.411) | 0.020(0.006,0.051) | 0.734(-0.539,2.023) |
| Slovenia | 0.071(0.039,0.131) | 0.018(0.010,0.033) | 0.019(0.005,0.036) | 0.006(0.002,0.012) | -2.710(-4.089,-1.311) |
| Belarus | 0.810(0.420,1.431) | 0.036(0.018,0.063) | 0.383(0.112,0.934) | 0.025(0.007,0.061) | 0.276(-1.032,1.601) |
| Estonia | 0.150(0.076,0.295) | 0.046(0.023,0.091) | 0.049(0.012,0.110) | 0.024(0.006,0.054) | -1.284(-2.104,-0.458) |
| Latvia | 0.190(0.085,0.377) | 0.036(0.016,0.071) | 0.056(0.014,0.119) | 0.020(0.005,0.042) | -1.011(-2.018,0.007) |
| Lithuania | 0.265(0.123,0.459) | 0.034(0.016,0.059) | 0.099(0.028,0.259) | 0.025(0.007,0.067) | -1.083(-2.164,0.010) |
| Republic of Moldova | 1.922(0.840,3.868) | 0.166(0.073,0.334) | 0.338(0.163,0.573) | 0.067(0.033,0.115) | -1.610(-2.564,-0.648) |
| Russian Federation | 18.620(10.899,29.030) | 0.057(0.033,0.089) | 6.139(2.744,9.104) | 0.024(0.011,0.036) | -2.040(-2.886,-1.186) |
| Ukraine | 3.482(1.455,8.578) | 0.032(0.014,0.080) | 1.765(0.578,4.199) | 0.028(0.009,0.066) | -0.807(-1.475,-0.133) |
| Brunei Darussalam | 0.058(0.020,0.136) | 0.071(0.025,0.166) | 0.034(0.014,0.068) | 0.038(0.015,0.076) | -1.895(-2.386,-1.402) |
| Japan | 4.141(1.678,7.623) | 0.019(0.008,0.034) | 2.684(1.107,3.914) | 0.018(0.007,0.026) | -0.129(-1.212,0.967) |
| Republic of Korea | 2.252(0.655,5.780) | 0.020(0.006,0.052) | 0.493(0.152,1.164) | 0.008(0.002,0.019) | -3.339(-4.761,-1.894) |
| Singapore | 0.169(0.075,0.333) | 0.028(0.012,0.055) | 0.136(0.042,0.264) | 0.018(0.006,0.035) | -1.124(-2.412,0.181) |
| Australia | 0.987(0.550,1.649) | 0.028(0.015,0.046) | 0.754(0.316,1.330) | 0.017(0.007,0.029) | -1.560(-2.726,-0.379) |
| New Zealand | 0.315(0.194,0.490) | 0.043(0.026,0.066) | 0.231(0.140,0.346) | 0.024(0.015,0.036) | -2.188(-3.121,-1.247) |
| Andorra | 0.005(0.001,0.013) | 0.061(0.016,0.152) | 0.004(0.001,0.008) | 0.042(0.015,0.084) | -0.756(-1.992,0.496) |
| Austria | 0.366(0.216,0.591) | 0.029(0.017,0.047) | 0.257(0.120,0.422) | 0.021(0.010,0.035) | -0.925(-1.851,0.010) |
| Belgium | 0.504(0.248,0.863) | 0.030(0.015,0.051) | 0.564(0.227,1.007) | 0.031(0.012,0.055) | -0.261(-1.287,0.775) |
| Cyprus | 0.094(0.030,0.213) | 0.050(0.016,0.114) | 0.051(0.022,0.102) | 0.025(0.011,0.050) | -1.267(-2.344,-0.178) |
| Denmark | 0.373(0.212,0.622) | 0.046(0.026,0.076) | 0.170(0.072,0.308) | 0.019(0.008,0.034) | -2.700(-3.939,-1.445) |
| Finland | 0.309(0.161,0.545) | 0.034(0.018,0.059) | 0.125(0.044,0.233) | 0.015(0.005,0.028) | -2.541(-3.492,-1.581) |
| France | 3.066(1.824,5.057) | 0.028(0.016,0.046) | 2.666(0.791,6.099) | 0.024(0.007,0.056) | -0.701(-1.828,0.439) |
| Germany | 2.655(1.474,4.730) | 0.022(0.012,0.039) | 1.707(0.587,3.398) | 0.015(0.005,0.030) | -0.865(-2.112,0.397) |
| Greece | 0.303(0.101,0.574) | 0.015(0.005,0.029) | 0.209(0.110,0.319) | 0.016(0.008,0.024) | 0.138(-1.009,1.299) |
| Iceland | 0.013(0.007,0.023) | 0.022(0.011,0.039) | 0.006(0.003,0.011) | 0.009(0.004,0.017) | -2.097(-3.504,-0.669) |
| Ireland | 0.208(0.111,0.358) | 0.022(0.012,0.038) | 0.185(0.069,0.363) | 0.019(0.007,0.038) | -1.040(-2.295,0.231) |
| Israel | 1.049(0.498,1.899) | 0.073(0.035,0.133) | 1.239(0.640,2.000) | 0.051(0.026,0.082) | -1.033(-1.899,-0.159) |
| Italy | 2.621(1.668,4.356) | 0.030(0.019,0.049) | 1.904(0.653,3.745) | 0.026(0.009,0.050) | -0.207(-1.198,0.794) |
| Luxembourg | 0.024(0.009,0.041) | 0.039(0.015,0.066) | 0.030(0.014,0.047) | 0.031(0.015,0.049) | -1.646(-2.617,-0.666) |
| Malta | 0.023(0.010,0.042) | 0.028(0.012,0.051) | 0.024(0.011,0.049) | 0.040(0.019,0.082) | 0.262(-0.441,0.971) |
| Netherlands | 1.286(0.738,2.005) | 0.051(0.029,0.079) | 0.868(0.379,1.450) | 0.034(0.015,0.057) | -1.198(-2.248,-0.136) |
| Norway | 0.130(0.079,0.232) | 0.018(0.011,0.031) | 0.075(0.033,0.126) | 0.008(0.004,0.014) | -2.084(-3.191,-0.965) |
| Portugal | 0.853(0.424,1.773) | 0.042(0.021,0.087) | 0.383(0.137,0.729) | 0.030(0.011,0.056) | -1.488(-2.629,-0.333) |
| Spain | 2.899(1.554,4.978) | 0.038(0.020,0.065) | 1.760(0.595,3.691) | 0.028(0.009,0.058) | -1.029(-2.118,0.072) |
| Sweden | 0.180(0.041,0.362) | 0.013(0.003,0.026) | 0.226(0.109,0.391) | 0.013(0.006,0.023) | 0.520(-0.395,1.444) |
| Switzerland | 0.272(0.134,0.497) | 0.025(0.012,0.046) | 0.164(0.065,0.348) | 0.013(0.005,0.028) | -2.178(-3.232,-1.111) |
| United Kingdom | 2.017(0.524,3.820) | 0.020(0.005,0.038) | 1.904(0.878,2.981) | 0.017(0.008,0.026) | -0.228(-1.330,0.885) |
| Argentina | 9.623(5.365,15.548) | 0.102(0.057,0.165) | 7.669(4.436,12.320) | 0.079(0.045,0.127) | -0.504(-1.368,0.368) |
| Chile | 3.099(1.350,5.248) | 0.084(0.037,0.143) | 1.874(0.981,2.908) | 0.054(0.028,0.084) | -0.910(-1.769,-0.043) |
| Uruguay | 0.944(0.494,1.491) | 0.124(0.065,0.195) | 0.594(0.323,0.955) | 0.094(0.051,0.151) | -0.937(-1.853,-0.013) |
| Canada | 1.554(0.849,2.532) | 0.029(0.016,0.047) | 1.084(0.468,2.001) | 0.018(0.008,0.033) | -1.848(-3.295,-0.379) |
| United States of America | 19.482(13.633,26.956) | 0.038(0.026,0.052) | 13.372(8.435,18.453) | 0.023(0.015,0.032) | -0.906(-2.213,0.418) |
| Antigua and Barbuda | 0.010(0.005,0.016) | 0.058(0.030,0.092) | 0.009(0.005,0.014) | 0.054(0.030,0.088) | -0.217(-1.134,0.710) |
| Bahamas | 0.066(0.039,0.105) | 0.088(0.052,0.140) | 0.053(0.030,0.085) | 0.067(0.038,0.108) | -1.154(-1.594,-0.711) |
| Barbados | 0.134(0.080,0.202) | 0.226(0.135,0.341) | 0.061(0.036,0.094) | 0.133(0.076,0.206) | -0.976(-2.114,0.175) |
| Belize | 0.041(0.021,0.085) | 0.054(0.027,0.111) | 0.043(0.025,0.066) | 0.037(0.022,0.057) | -0.657(-0.861,-0.453) |
| Cuba | 4.312(2.071,7.368) | 0.187(0.090,0.319) | 1.068(0.506,2.014) | 0.063(0.030,0.119) | -1.066(-2.241,0.124) |
| Dominica | 0.016(0.005,0.036) | 0.068(0.022,0.158) | 0.010(0.003,0.022) | 0.074(0.025,0.173) | 0.741(0.005,1.481) |
| Dominican Republic | 1.187(0.340,3.183) | 0.048(0.014,0.127) | 1.649(0.529,4.269) | 0.061(0.019,0.157) | 0.805(0.128,1.486) |
| Grenada | 0.052(0.027,0.091) | 0.167(0.086,0.292) | 0.026(0.013,0.045) | 0.126(0.063,0.218) | -0.574(-1.302,0.159) |
| Guyana | 0.236(0.100,0.445) | 0.089(0.038,0.168) | 0.084(0.036,0.186) | 0.042(0.018,0.094) | -0.193(-0.759,0.376) |
| Haiti | 6.362(0.845,23.119) | 0.246(0.034,0.882) | 8.136(1.801,25.146) | 0.201(0.045,0.620) | -0.391(-0.714,-0.066) |
| Jamaica | 0.572(0.260,1.132) | 0.073(0.033,0.146) | 0.253(0.125,0.528) | 0.045(0.022,0.095) | -1.289(-1.923,-0.652) |
| Saint Lucia | 0.039(0.020,0.065) | 0.080(0.042,0.136) | 0.021(0.011,0.034) | 0.073(0.040,0.118) | -0.252(-0.889,0.389) |
| Saint Vincent and the Grenadines | 0.033(0.008,0.067) | 0.085(0.022,0.173) | 0.019(0.011,0.031) | 0.077(0.044,0.129) | -0.309(-0.631,0.014) |
| Suriname | 0.045(0.015,0.107) | 0.037(0.013,0.088) | 0.061(0.020,0.152) | 0.045(0.015,0.113) | 1.304(0.856,1.754) |
| Trinidad and Tobago | 0.413(0.233,0.680) | 0.107(0.060,0.176) | 0.184(0.104,0.294) | 0.070(0.039,0.112) | -1.003(-1.940,-0.058) |
| Bolivia (Plurinational State of) | 4.391(1.397,10.390) | 0.177(0.057,0.416) | 3.423(1.134,7.357) | 0.105(0.035,0.227) | -2.525(-3.240,-1.805) |
| Ecuador | 2.664(1.511,4.367) | 0.074(0.042,0.122) | 3.143(1.235,5.491) | 0.066(0.026,0.115) | -0.131(-1.033,0.779) |
| Peru | 10.590(4.455,20.991) | 0.138(0.058,0.274) | 6.548(2.283,13.709) | 0.074(0.026,0.154) | -2.154(-3.008,-1.293) |
| Colombia | 12.063(6.987,19.637) | 0.112(0.065,0.183) | 8.373(3.651,16.355) | 0.084(0.037,0.165) | -0.207(-1.091,0.684) |
| Costa Rica | 0.954(0.441,1.567) | 0.091(0.042,0.150) | 0.693(0.239,1.440) | 0.072(0.025,0.149) | -0.971(-1.800,-0.136) |
| El Salvador | 1.026(0.498,1.839) | 0.052(0.025,0.093) | 0.605(0.275,1.045) | 0.035(0.016,0.061) | -1.398(-1.755,-1.041) |
| Guatemala | 3.738(1.947,7.749) | 0.099(0.052,0.203) | 2.304(1.265,3.465) | 0.049(0.027,0.074) | -1.745(-2.332,-1.155) |
| Honduras | 1.095(0.369,2.527) | 0.053(0.018,0.123) | 0.911(0.336,1.929) | 0.030(0.011,0.063) | -2.297(-2.555,-2.040) |
| Mexico | 22.315(13.049,38.256) | 0.072(0.042,0.124) | 16.017(8.251,23.829) | 0.053(0.027,0.079) | -0.646(-1.242,-0.047) |
| Nicaragua | 1.484(0.622,2.830) | 0.087(0.036,0.165) | 0.964(0.486,1.692) | 0.052(0.026,0.091) | -1.746(-2.623,-0.862) |
| Panama | 0.909(0.551,1.430) | 0.117(0.071,0.185) | 1.177(0.693,1.733) | 0.108(0.064,0.160) | -0.336(-0.882,0.213) |
| Venezuela (Bolivarian Republic of) | 6.043(2.597,10.449) | 0.092(0.040,0.160) | 9.402(5.547,14.168) | 0.153(0.090,0.231) | 1.225(0.331,2.127) |
| Brazil | 56.704(38.339,85.962) | 0.117(0.079,0.177) | 36.553(19.188,51.493) | 0.081(0.043,0.115) | -0.924(-1.690,-0.153) |
| Paraguay | 0.920(0.336,1.970) | 0.059(0.021,0.125) | 0.961(0.295,2.109) | 0.051(0.016,0.113) | -0.386(-1.042,0.273) |
| Algeria | 9.269(2.102,23.038) | 0.092(0.021,0.228) | 7.670(2.258,19.336) | 0.062(0.018,0.155) | -0.901(-2.004,0.213) |
| Bahrain | 0.035(0.009,0.086) | 0.023(0.006,0.058) | 0.029(0.007,0.073) | 0.010(0.002,0.026) | -2.863(-4.269,-1.436) |
| Egypt | 7.820(1.254,27.868) | 0.039(0.006,0.139) | 5.174(0.484,21.461) | 0.015(0.001,0.063) | -2.867(-3.910,-1.813) |
| Iran (Islamic Republic of) | 6.277(2.498,16.224) | 0.026(0.010,0.066) | 3.655(1.496,6.973) | 0.018(0.008,0.035) | -0.819(-1.985,0.360) |
| Iraq | 10.084(1.473,29.571) | 0.135(0.020,0.394) | 4.748(1.381,11.902) | 0.037(0.011,0.093) | -4.178(-5.077,-3.271) |
| Jordan | 1.161(0.377,2.793) | 0.078(0.025,0.187) | 1.693(0.619,3.775) | 0.049(0.018,0.109) | -2.363(-3.308,-1.408) |
| Kuwait | 0.326(0.167,0.632) | 0.064(0.033,0.123) | 0.158(0.073,0.308) | 0.019(0.009,0.038) | -3.268(-3.877,-2.655) |
| Lebanon | 0.643(0.196,1.526) | 0.068(0.021,0.161) | 0.444(0.145,1.055) | 0.036(0.012,0.086) | -2.224(-3.745,-0.679) |
| Libya | 1.417(0.383,3.724) | 0.085(0.023,0.223) | 1.492(0.419,3.800) | 0.107(0.029,0.275) | 1.355(0.163,2.560) |
| Morocco | 5.864(1.705,15.761) | 0.065(0.019,0.175) | 3.714(1.269,8.629) | 0.040(0.014,0.094) | -1.229(-1.365,-1.093) |
| Palestine | 0.399(0.105,1.038) | 0.046(0.012,0.119) | 0.483(0.120,1.105) | 0.027(0.007,0.062) | -1.068(-3.371,1.290) |
| Oman | 0.388(0.111,0.987) | 0.051(0.015,0.130) | 0.433(0.132,0.981) | 0.038(0.011,0.085) | -0.631(-1.903,0.658) |
| Qatar | 0.018(0.004,0.048) | 0.016(0.003,0.043) | 0.045(0.011,0.109) | 0.010(0.003,0.024) | -1.341(-2.308,-0.365) |
| Saudi Arabia | 3.898(1.445,9.157) | 0.064(0.024,0.150) | 2.252(0.510,5.097) | 0.031(0.007,0.071) | -2.319(-3.223,-1.407) |
| Syrian Arab Republic | 0.951(0.321,2.313) | 0.017(0.006,0.042) | 0.724(0.180,1.614) | 0.021(0.005,0.047) | -0.146(-0.732,0.443) |
| Tunisia | 2.631(0.732,7.347) | 0.091(0.025,0.253) | 1.337(0.405,3.260) | 0.051(0.015,0.124) | -1.779(-2.829,-0.718) |
| T眉rkiye | 42.267(15.438,89.592) | 0.221(0.081,0.469) | 16.532(7.560,29.319) | 0.092(0.042,0.163) | -3.008(-4.155,-1.848) |
| United Arab Emirates | 0.352(0.104,0.802) | 0.066(0.020,0.151) | 0.314(0.100,0.695) | 0.025(0.008,0.055) | -2.776(-3.448,-2.099) |
| Yemen | 3.656(0.572,12.560) | 0.056(0.009,0.191) | 5.679(1.030,15.767) | 0.044(0.008,0.122) | -0.767(-2.141,0.626) |
| Afghanistan | 4.395(0.718,15.447) | 0.113(0.019,0.394) | 8.818(1.721,25.501) | 0.069(0.013,0.199) | -1.174(-2.310,-0.025) |
| Bangladesh | 24.409(5.153,72.300) | 0.054(0.011,0.159) | 19.022(4.719,47.705) | 0.044(0.011,0.109) | -1.391(-2.112,-0.665) |
| Bhutan | 0.131(0.024,0.467) | 0.055(0.010,0.194) | 0.080(0.015,0.218) | 0.046(0.009,0.126) | -1.539(-2.529,-0.540) |
| India | 178.359(54.668,371.154) | 0.058(0.018,0.121) | 89.607(54.808,143.492) | 0.026(0.016,0.041) | -3.083(-3.770,-2.391) |
| Nepal | 3.380(0.834,10.448) | 0.044(0.011,0.134) | 3.150(0.640,8.321) | 0.037(0.007,0.097) | -1.338(-2.365,-0.301) |
| Pakistan | 67.392(22.331,146.116) | 0.148(0.050,0.321) | 164.146(58.851,351.753) | 0.206(0.074,0.442) | 1.213(1.021,1.406) |
| Angola | 32.336(6.543,68.259) | 0.712(0.152,1.490) | 41.143(15.953,73.249) | 0.289(0.112,0.514) | -2.763(-4.148,-1.358) |
| Central African Republic | 6.633(1.725,13.231) | 0.572(0.155,1.130) | 9.380(3.286,17.478) | 0.443(0.156,0.823) | -0.663(-2.024,0.717) |
| Congo | 4.151(1.277,7.777) | 0.424(0.132,0.791) | 4.493(2.059,7.610) | 0.250(0.114,0.424) | -1.635(-2.880,-0.374) |
| Democratic Republic of the Congo | 87.173(21.118,171.138) | 0.512(0.130,0.995) | 80.951(34.180,144.718) | 0.229(0.097,0.409) | -2.038(-3.281,-0.780) |
| Equatorial Guinea | 0.992(0.272,1.958) | 0.531(0.152,1.036) | 1.465(0.545,3.502) | 0.266(0.098,0.635) | -2.857(-4.020,-1.680) |
| Gabon | 1.288(0.469,2.289) | 0.341(0.126,0.602) | 1.657(0.729,2.961) | 0.278(0.122,0.497) | -0.325(-1.625,0.992) |
| Burundi | 34.634(11.763,69.593) | 1.412(0.486,2.818) | 31.757(14.027,61.576) | 0.590(0.261,1.138) | -2.603(-3.247,-1.954) |
| Comoros | 1.945(0.736,3.642) | 0.996(0.378,1.855) | 1.573(0.772,2.811) | 0.704(0.345,1.260) | -1.442(-1.906,-0.977) |
| Djibouti | 1.229(0.489,2.227) | 0.772(0.310,1.394) | 2.491(1.148,4.507) | 0.650(0.299,1.175) | -0.575(-1.173,0.026) |
| Eritrea | 14.113(5.175,25.755) | 0.955(0.353,1.735) | 17.694(8.201,33.047) | 0.755(0.351,1.408) | -0.866(-1.478,-0.251) |
| Ethiopia | 248.685(67.408,512.433) | 1.102(0.302,2.261) | 214.086(105.454,355.688) | 0.525(0.259,0.870) | -2.664(-3.768,-1.547) |
| Kenya | 31.381(14.186,47.714) | 0.303(0.138,0.458) | 43.233(25.218,60.865) | 0.246(0.143,0.348) | 0.421(-0.616,1.468) |
| Madagascar | 44.059(18.176,77.401) | 0.876(0.365,1.528) | 59.072(30.374,100.548) | 0.541(0.278,0.920) | -1.091(-1.676,-0.503) |
| Malawi | 96.838(34.645,178.795) | 2.285(0.827,4.174) | 105.993(43.113,212.638) | 1.408(0.572,2.835) | -1.367(-1.910,-0.822) |
| Mauritius | 0.033(0.021,0.050) | 0.011(0.007,0.016) | 0.018(0.009,0.030) | 0.009(0.005,0.016) | -0.731(-1.413,-0.044) |
| Mozambique | 11.019(3.679,26.387) | 0.190(0.064,0.454) | 11.839(4.273,31.327) | 0.090(0.032,0.236) | -2.081(-2.881,-1.275) |
| Rwanda | 47.946(16.562,88.715) | 1.519(0.530,2.796) | 30.755(15.275,55.631) | 0.668(0.332,1.208) | -3.264(-3.778,-2.748) |
| Seychelles | 0.000(0.000,0.000) | 0.000(0.000,0.000) | 0.000(0.000,0.000) | 0.000(0.000,0.000) | 0.808(-0.173,1.797) |
| Somalia | 30.141(9.653,62.076) | 0.830(0.269,1.695) | 56.941(20.867,108.858) | 0.607(0.224,1.152) | -1.053(-1.867,-0.233) |
| United Republic of Tanzania | 131.240(49.126,232.773) | 1.179(0.445,2.076) | 172.875(82.571,306.833) | 0.765(0.366,1.354) | -0.960(-1.633,-0.283) |
| Uganda | 118.495(58.323,200.081) | 1.492(0.749,2.497) | 265.776(134.263,473.844) | 1.450(0.735,2.576) | -0.112(-0.761,0.541) |
| Zambia | 40.230(14.749,71.770) | 1.152(0.430,2.037) | 51.873(23.583,97.429) | 0.674(0.306,1.266) | -1.896(-2.653,-1.133) |
| Botswana | 0.421(0.140,0.916) | 0.077(0.026,0.168) | 0.976(0.300,2.068) | 0.151(0.046,0.321) | 2.657(1.617,3.706) |
| Lesotho | 0.439(0.154,0.939) | 0.070(0.024,0.149) | 0.785(0.262,1.731) | 0.132(0.044,0.292) | 2.776(1.543,4.025) |
| Namibia | 0.691(0.235,1.618) | 0.125(0.043,0.292) | 1.522(0.576,3.266) | 0.198(0.075,0.427) | 2.157(1.072,3.254) |
| South Africa | 4.819(2.125,8.913) | 0.038(0.017,0.070) | 6.741(4.159,10.711) | 0.047(0.029,0.075) | 0.409(-0.289,1.113) |
| Eswatini | 0.360(0.109,0.860) | 0.101(0.031,0.239) | 0.545(0.166,1.221) | 0.142(0.043,0.318) | 1.585(0.291,2.897) |
| Zimbabwe | 5.239(1.764,11.869) | 0.118(0.040,0.267) | 17.337(5.323,38.735) | 0.297(0.091,0.663) | 5.121(3.395,6.876) |
| Benin | 15.579(5.118,29.830) | 0.674(0.227,1.278) | 36.800(16.617,66.007) | 0.649(0.295,1.157) | 0.023(-1.054,1.112) |
| Burkina Faso | 32.120(11.134,61.420) | 0.718(0.254,1.364) | 64.651(28.290,116.374) | 0.672(0.298,1.201) | 0.257(-0.776,1.300) |
| Cameroon | 31.441(11.685,58.848) | 0.689(0.263,1.277) | 90.551(40.667,155.622) | 0.724(0.326,1.243) | 0.568(-0.271,1.414) |
| Cabo Verde | 0.186(0.079,0.350) | 0.128(0.054,0.240) | 0.464(0.162,0.837) | 0.337(0.119,0.607) | 1.748(0.898,2.604) |
| Chad | 15.497(5.165,30.075) | 0.565(0.194,1.081) | 55.666(22.557,97.284) | 0.667(0.274,1.162) | 0.924(-0.204,2.065) |
| C么te d'Ivoire | 36.219(14.707,66.249) | 0.680(0.281,1.232) | 66.144(29.097,120.769) | 0.619(0.273,1.125) | 0.181(-0.360,0.725) |
| Gambia | 2.605(1.010,5.040) | 0.603(0.240,1.154) | 4.808(2.018,9.196) | 0.524(0.221,1.000) | -0.361(-1.242,0.528) |
| Ghana | 73.885(19.873,151.102) | 1.184(0.319,2.411) | 51.341(26.327,93.853) | 0.430(0.220,0.784) | -4.169(-5.325,-2.999) |
| Guinea | 19.145(6.519,38.674) | 0.725(0.259,1.441) | 27.374(11.937,54.342) | 0.486(0.214,0.961) | -0.689(-1.282,-0.092) |
| Guinea-Bissau | 3.827(1.342,7.573) | 0.851(0.303,1.673) | 4.440(2.058,7.906) | 0.537(0.250,0.957) | -1.033(-2.051,-0.004) |
| Liberia | 10.004(3.140,19.937) | 0.925(0.300,1.817) | 11.951(5.163,20.735) | 0.591(0.255,1.024) | -1.459(-2.262,-0.650) |
| Mali | 20.948(8.212,39.439) | 0.531(0.215,0.986) | 37.770(17.438,69.443) | 0.351(0.165,0.638) | -0.935(-1.770,-0.093) |
| Mauritania | 4.177(1.727,7.728) | 0.489(0.205,0.897) | 6.835(3.336,11.916) | 0.396(0.193,0.690) | -0.864(-1.564,-0.159) |
| Niger | 35.365(10.169,75.174) | 0.913(0.271,1.917) | 60.412(24.276,115.103) | 0.508(0.207,0.960) | -1.772(-2.843,-0.690) |
| Nigeria | 290.420(123.266,497.645) | 0.790(0.346,1.339) | 620.431(292.004,932.311) | 0.658(0.312,0.986) | -0.382(-1.300,0.544) |
| Sao Tome and Principe | 0.408(0.154,0.763) | 0.775(0.292,1.447) | 0.217(0.101,0.390) | 0.295(0.137,0.534) | -2.520(-3.233,-1.803) |
| Senegal | 24.702(9.115,45.838) | 0.715(0.270,1.318) | 26.218(13.249,45.788) | 0.446(0.226,0.779) | -1.285(-2.119,-0.444) |
| Sierra Leone | 15.152(4.690,30.990) | 0.874(0.282,1.753) | 21.995(10.208,39.106) | 0.667(0.312,1.182) | -0.611(-1.553,0.340) |
| Togo | 9.798(3.816,17.443) | 0.593(0.234,1.051) | 15.102(7.415,27.461) | 0.490(0.240,0.890) | -0.261(-1.189,0.676) |
| American Samoa | 0.001(0.000,0.004) | 0.007(0.001,0.022) | 0.002(0.001,0.007) | 0.018(0.004,0.049) | 3.089(1.805,4.390) |
| Bermuda | 0.011(0.004,0.020) | 0.097(0.034,0.182) | 0.005(0.002,0.010) | 0.062(0.021,0.123) | -1.158(-1.399,-0.917) |
| Cook Islands | 0.001(0.000,0.002) | 0.010(0.002,0.026) | 0.000(0.000,0.001) | 0.012(0.001,0.038) | -0.347(-0.566,-0.128) |
| Greenland | 0.014(0.002,0.035) | 0.106(0.013,0.271) | 0.002(0.001,0.006) | 0.020(0.005,0.055) | -4.124(-5.339,-2.894) |
| Guam | 0.007(0.002,0.016) | 0.017(0.005,0.041) | 0.013(0.006,0.022) | 0.039(0.019,0.065) | 4.951(4.386,5.519) |
| Monaco | 0.000(0.000,0.001) | 0.008(0.001,0.025) | 0.000(0.000,0.001) | 0.009(0.002,0.026) | 0.166(-0.373,0.708) |
| Nauru | 0.001(0.000,0.003) | 0.023(0.006,0.065) | 0.002(0.000,0.005) | 0.047(0.011,0.133) | 2.258(2.043,2.473) |
| Niue | 0.000(0.000,0.000) | 0.020(0.005,0.060) | 0.000(0.000,0.001) | 0.054(0.010,0.193) | 2.519(1.803,3.240) |
| Northern Mariana Islands | 0.001(0.000,0.002) | 0.006(0.001,0.019) | 0.001(0.000,0.003) | 0.011(0.003,0.031) | 3.721(3.137,4.309) |
| Palau | 0.001(0.000,0.002) | 0.021(0.006,0.052) | 0.001(0.000,0.001) | 0.022(0.007,0.050) | 0.651(-0.712,2.033) |
| Puerto Rico | 1.016(0.426,1.735) | 0.109(0.046,0.187) | 0.228(0.098,0.362) | 0.052(0.022,0.084) | -1.863(-2.646,-1.074) |
| Saint Kitts and Nevis | 0.006(0.003,0.010) | 0.043(0.022,0.079) | 0.004(0.002,0.008) | 0.042(0.021,0.086) | 0.661(-0.096,1.424) |
| San Marino | 0.002(0.000,0.005) | 0.049(0.011,0.126) | 0.002(0.000,0.004) | 0.037(0.008,0.089) | -0.669(-2.515,1.211) |
| Tokelau | 0.000(0.000,0.000) | 0.016(0.004,0.046) | 0.000(0.000,0.000) | 0.036(0.007,0.127) | 1.335(0.767,1.906) |
| Tuvalu | 0.001(0.000,0.003) | 0.029(0.006,0.091) | 0.001(0.000,0.002) | 0.026(0.007,0.069) | -0.022(-0.179,0.135) |
| United States Virgin Islands | 0.011(0.003,0.026) | 0.037(0.010,0.089) | 0.002(0.001,0.006) | 0.019(0.004,0.049) | -1.002(-1.719,-0.279) |
| South Sudan | 24.796(9.179,46.652) | 1.011(0.377,1.892) | 48.988(19.919,91.391) | 1.255(0.511,2.336) | 0.668(0.020,1.320) |
| Sudan | 6.750(1.037,28.826) | 0.083(0.013,0.350) | 9.653(1.823,26.122) | 0.062(0.012,0.169) | -0.895(-1.884,0.104) |
