# Supplementary material for: Global, regional, and national epidemiology of childhood Burkitt Lymphoma from 1990 to 2021: statistical analysis of incidence, mortality, and DALYs
Source: Front Public Health. 2025 Jul 16;13:1560003. doi: 10.3389/fpubh.2025.1560003 (PMC12307453; doi:10.3389/fpubh.2025.1560003)
Supplement: Supplementary file 11 [file Table_5.docx]

Table S5: The number of DALYs and DALYs rates of Burkitt lymphoma in children in 204 countries and regions in 1990 and 2021, as well as the annual percentage change (EAPC) from 1990 to 2021.

| Location |  | Rate per 100,000 (95% UI) |  |  |  |
| --- | --- | --- | --- | --- | --- |
|  | 1990 |  | 2021 |  | 1990-2021 |
|  | Number of DALYs | DALYs rate | Number of DALYs | DALYs rate | EAPC |
| China | 11155.054(4425.392,19006.841) | 3.775(1.501,6.427) | 2612.173(1365.971,4746.122) | 1.080(0.555,1.954) | -5.962(-6.734,-5.183) |
| Democratic People's Republic of Korea | 130.854(34.433,357.455) | 2.371(0.626,6.437) | 71.715(15.324,183.644) | 1.623(0.345,4.166) | -1.728(-2.537,-0.912) |
| Taiwan (Province of China) | 95.864(55.906,166.084) | 1.832(1.065,3.182) | 40.271(12.839,77.673) | 1.418(0.449,2.748) | -0.128(-1.004,0.755) |
| Cambodia | 118.294(16.391,429.091) | 2.688(0.383,9.669) | 77.547(22.700,204.244) | 1.620(0.474,4.271) | -2.107(-3.077,-1.128) |
| Indonesia | 755.382(174.720,1801.259) | 1.198(0.273,2.868) | 805.480(358.810,1499.523) | 1.281(0.564,2.395) | -1.519(-2.489,-0.540) |
| Lao People's Democratic Republic | 37.384(3.789,148.005) | 2.151(0.225,8.465) | 44.701(12.041,123.871) | 2.098(0.567,5.802) | -2.570(-3.956,-1.164) |
| Malaysia | 98.030(29.635,269.033) | 1.606(0.486,4.408) | 63.736(16.223,169.010) | 0.879(0.223,2.328) | -2.332(-3.440,-1.212) |
| Maldives | 4.713(0.890,14.843) | 4.738(0.918,14.746) | 1.740(0.467,4.060) | 1.844(0.497,4.306) | -3.202(-4.339,-2.052) |
| Myanmar | 444.654(48.311,1802.401) | 3.263(0.350,13.284) | 263.980(72.711,693.362) | 1.813(0.497,4.782) | -2.592(-3.257,-1.923) |
| Philippines | 762.942(284.970,1360.288) | 3.257(1.226,5.785) | 601.145(364.538,937.844) | 1.888(1.142,2.945) | -1.484(-2.467,-0.492) |
| Sri Lanka | 150.422(45.489,356.479) | 2.872(0.870,6.809) | 87.073(24.114,199.296) | 1.780(0.492,4.066) | -1.436(-2.322,-0.541) |
| Thailand | 292.238(55.143,774.927) | 1.829(0.342,4.859) | 139.756(48.239,344.726) | 1.499(0.514,3.714) | -2.307(-3.584,-1.014) |
| Timor-Leste | 6.716(0.688,24.869) | 2.083(0.229,7.605) | 6.341(1.612,19.324) | 1.319(0.335,4.015) | -2.225(-3.170,-1.271) |
| Viet Nam | 414.258(84.593,1276.072) | 1.662(0.341,5.112) | 593.711(91.997,1868.264) | 2.521(0.390,7.947) | 1.760(0.344,3.196) |
| Fiji | 6.028(1.461,15.425) | 2.317(0.562,5.919) | 12.535(3.621,31.735) | 4.984(1.444,12.599) | 2.830(2.563,3.098) |
| Kiribati | 0.087(0.016,0.273) | 0.295(0.056,0.925) | 0.108(0.019,0.368) | 0.279(0.048,0.949) | -0.811(-1.061,-0.560) |
| Marshall Islands | 0.184(0.045,0.499) | 0.912(0.226,2.484) | 0.371(0.090,1.074) | 2.296(0.562,6.627) | 2.532(2.223,2.841) |
| Micronesia (Federated States of) | 0.401(0.081,1.242) | 0.943(0.189,2.918) | 0.545(0.133,1.681) | 1.919(0.477,5.825) | 2.666(2.476,2.856) |
| Papua New Guinea | 32.735(6.207,116.619) | 1.991(0.379,7.115) | 134.053(24.418,422.335) | 3.525(0.639,11.118) | 1.947(1.622,2.274) |
| Samoa | 0.762(0.093,2.718) | 1.155(0.140,4.133) | 1.009(0.127,3.137) | 1.326(0.168,4.135) | 0.201(-2.814,3.309) |
| Solomon Islands | 1.512(0.320,4.762) | 1.018(0.214,3.223) | 4.781(1.176,14.691) | 1.950(0.478,6.000) | 2.036(1.745,2.328) |
| Tonga | 1.311(0.330,3.603) | 3.297(0.825,9.087) | 1.825(0.438,5.385) | 4.950(1.173,14.716) | 0.825(-0.502,2.169) |
| Vanuatu | 0.509(0.116,1.517) | 0.778(0.175,2.320) | 1.581(0.375,4.670) | 1.450(0.344,4.291) | 2.157(1.856,2.459) |
| Armenia | 12.026(5.442,25.981) | 1.222(0.553,2.633) | 4.956(2.497,8.895) | 0.891(0.446,1.608) | -0.499(-1.115,0.121) |
| Azerbaijan | 29.348(8.877,80.622) | 1.283(0.389,3.511) | 22.551(7.910,58.223) | 1.012(0.347,2.659) | -1.592(-2.203,-0.977) |
| Georgia | 113.194(28.409,216.058) | 8.791(2.209,16.776) | 16.443(7.033,29.426) | 2.381(1.019,4.263) | -4.305(-5.058,-3.547) |
| Kazakhstan | 136.669(52.844,302.440) | 2.814(1.089,6.226) | 61.519(24.092,122.707) | 1.213(0.476,2.419) | -3.445(-4.863,-2.005) |
| Kyrgyzstan | 33.233(14.400,69.968) | 2.128(0.926,4.445) | 24.896(11.144,53.619) | 1.165(0.522,2.512) | -2.248(-3.308,-1.175) |
| Mongolia | 23.210(3.463,86.676) | 2.709(0.406,10.042) | 30.594(9.056,66.601) | 3.014(0.892,6.568) | -1.812(-4.884,1.359) |
| Tajikistan | 3.163(0.547,11.465) | 0.138(0.025,0.491) | 2.964(0.889,8.146) | 0.087(0.026,0.239) | -2.521(-2.978,-2.061) |
| Turkmenistan | 15.249(5.037,43.658) | 1.052(0.356,2.962) | 11.319(4.866,24.739) | 0.794(0.342,1.734) | -1.348(-1.764,-0.930) |
| Uzbekistan | 64.458(26.758,158.452) | 0.791(0.331,1.936) | 71.854(33.478,169.964) | 0.763(0.355,1.802) | -0.390(-1.199,0.425) |
| Albania | 2.541(0.832,6.720) | 0.243(0.080,0.644) | 1.538(0.295,4.971) | 0.371(0.070,1.205) | 1.679(0.778,2.588) |
| Bosnia and Herzegovina | 12.778(3.061,35.871) | 1.232(0.295,3.472) | 4.473(1.238,10.861) | 0.946(0.261,2.300) | -0.880(-2.420,0.684) |
| Bulgaria | 15.957(6.678,33.240) | 0.975(0.409,2.037) | 8.222(3.323,15.098) | 0.882(0.353,1.618) | -0.440(-1.295,0.423) |
| Croatia | 25.186(10.789,45.803) | 2.673(1.146,4.860) | 13.316(4.397,25.480) | 2.348(0.781,4.523) | 0.208(-0.962,1.391) |
| Czechia | 49.996(28.752,84.131) | 2.398(1.386,4.025) | 23.087(6.049,53.188) | 1.420(0.374,3.270) | -1.616(-2.875,-0.340) |
| Hungary | 44.224(21.074,78.468) | 2.206(1.054,3.891) | 22.786(6.056,47.629) | 1.746(0.467,3.647) | -0.496(-1.734,0.756) |
| North Macedonia | 3.181(1.030,8.256) | 0.643(0.210,1.667) | 1.710(0.621,3.806) | 0.538(0.195,1.199) | -0.304(-1.436,0.841) |
| Montenegro | 7.056(3.261,12.856) | 4.639(2.144,8.472) | 2.028(0.654,4.269) | 1.896(0.605,4.009) | -2.831(-4.046,-1.600) |
| Poland | 148.095(44.715,303.783) | 1.621(0.499,3.324) | 93.795(34.037,141.583) | 1.683(0.609,2.547) | 0.019(-1.107,1.157) |
| Romania | 233.994(85.718,572.219) | 4.502(1.636,11.092) | 96.497(46.166,163.985) | 3.379(1.613,5.744) | -0.480(-1.618,0.672) |
| Serbia | 41.487(12.960,102.437) | 2.045(0.636,5.083) | 11.070(2.882,27.447) | 0.864(0.219,2.155) | -3.217(-4.279,-2.143) |
| Slovakia | 16.340(5.251,37.782) | 1.287(0.411,2.982) | 13.284(3.877,35.030) | 1.655(0.483,4.370) | 0.810(-0.454,2.090) |
| Slovenia | 5.936(3.233,10.945) | 1.494(0.811,2.750) | 1.643(0.445,3.090) | 0.552(0.149,1.041) | -2.564(-3.935,-1.173) |
| Belarus | 67.468(35.007,119.516) | 2.952(1.529,5.233) | 32.312(9.373,78.503) | 2.092(0.602,5.110) | 0.328(-0.976,1.649) |
| Estonia | 12.478(6.304,24.651) | 3.808(1.925,7.526) | 4.142(1.041,9.336) | 2.025(0.506,4.561) | -1.210(-2.021,-0.392) |
| Latvia | 15.882(7.098,31.358) | 2.976(1.328,5.875) | 4.708(1.186,10.026) | 1.654(0.415,3.546) | -0.990(-1.992,0.022) |
| Lithuania | 22.098(10.272,38.279) | 2.844(1.322,4.924) | 8.326(2.352,21.723) | 2.133(0.600,5.572) | -1.079(-2.152,0.005) |
| Republic of Moldova | 160.553(70.163,324.183) | 13.783(6.023,27.799) | 28.233(13.640,47.988) | 5.620(2.710,9.604) | -1.603(-2.555,-0.641) |
| Russian Federation | 1553.456(906.761,2431.334) | 4.713(2.747,7.383) | 514.951(228.969,764.150) | 2.025(0.892,3.013) | -2.002(-2.841,-1.155) |
| Ukraine | 288.856(120.570,709.171) | 2.674(1.116,6.561) | 146.032(47.641,348.136) | 2.287(0.737,5.496) | -0.777(-1.435,-0.115) |
| Brunei Darussalam | 4.760(1.682,11.148) | 5.786(2.045,13.533) | 2.788(1.134,5.615) | 3.091(1.254,6.238) | -1.852(-2.340,-1.361) |
| Japan | 348.518(141.363,641.080) | 1.558(0.638,2.877) | 232.448(96.098,340.892) | 1.548(0.637,2.288) | -0.036(-1.118,1.058) |
| Republic of Korea | 184.906(53.500,475.068) | 1.649(0.475,4.251) | 42.498(13.068,100.223) | 0.687(0.208,1.638) | -3.154(-4.580,-1.706) |
| Singapore | 13.983(6.258,27.649) | 2.282(1.021,4.546) | 11.800(3.614,22.762) | 1.560(0.478,3.022) | -0.988(-2.280,0.320) |
| Australia | 83.495(46.502,139.064) | 2.334(1.299,3.892) | 65.677(27.537,115.081) | 1.432(0.598,2.514) | -1.451(-2.625,-0.264) |
| New Zealand | 26.448(16.283,41.259) | 3.561(2.192,5.562) | 19.698(11.885,29.314) | 2.067(1.243,3.081) | -2.110(-3.050,-1.161) |
| Andorra | 0.467(0.121,1.149) | 5.222(1.349,12.985) | 0.368(0.129,0.732) | 3.687(1.282,7.411) | -0.674(-1.907,0.574) |
| Austria | 30.870(18.262,49.898) | 2.415(1.428,3.907) | 22.380(10.424,36.577) | 1.822(0.847,2.981) | -0.821(-1.746,0.113) |
| Belgium | 42.533(20.872,73.114) | 2.502(1.229,4.303) | 48.948(19.577,87.375) | 2.661(1.061,4.765) | -0.165(-1.190,0.871) |
| Cyprus | 7.827(2.523,17.867) | 4.155(1.335,9.503) | 4.436(1.864,8.875) | 2.152(0.903,4.311) | -1.125(-2.197,-0.041) |
| Denmark | 31.244(17.712,52.066) | 3.819(2.165,6.343) | 14.702(6.188,26.670) | 1.631(0.685,2.966) | -2.588(-3.827,-1.334) |
| Finland | 25.849(13.483,45.675) | 2.813(1.466,4.968) | 10.809(3.737,20.091) | 1.286(0.439,2.393) | -2.425(-3.378,-1.463) |
| France | 257.019(152.997,422.945) | 2.313(1.376,3.806) | 232.678(69.117,536.531) | 2.094(0.620,4.881) | -0.544(-1.669,0.593) |
| Germany | 222.622(123.724,396.352) | 1.839(1.021,3.274) | 148.313(50.853,296.306) | 1.313(0.451,2.625) | -0.759(-2.006,0.504) |
| Greece | 25.527(8.612,48.478) | 1.277(0.436,2.419) | 17.946(9.490,27.413) | 1.339(0.713,2.046) | 0.199(-0.946,1.358) |
| Iceland | 1.138(0.576,1.978) | 1.897(0.959,3.298) | 0.508(0.249,0.994) | 0.781(0.383,1.527) | -1.982(-3.391,-0.552) |
| Ireland | 17.407(9.335,30.005) | 1.823(0.977,3.147) | 16.131(6.027,31.489) | 1.660(0.617,3.253) | -0.870(-2.124,0.400) |
| Israel | 88.071(41.977,159.600) | 6.109(2.913,11.096) | 106.586(54.910,172.262) | 4.335(2.233,7.007) | -0.942(-1.806,-0.070) |
| Italy | 219.986(139.750,366.050) | 2.484(1.567,4.142) | 165.888(57.125,326.949) | 2.220(0.762,4.393) | -0.086(-1.080,0.917) |
| Luxembourg | 2.011(0.761,3.422) | 3.259(1.232,5.547) | 2.602(1.237,4.043) | 2.705(1.287,4.208) | -1.513(-2.476,-0.540) |
| Malta | 1.973(0.826,3.569) | 2.382(0.999,4.310) | 2.043(0.975,4.201) | 3.391(1.617,6.977) | 0.350(-0.352,1.058) |
| Netherlands | 109.194(62.491,170.162) | 4.298(2.459,6.699) | 75.962(33.097,126.654) | 2.966(1.291,4.954) | -1.073(-2.125,-0.010) |
| Norway | 10.953(6.631,19.669) | 1.477(0.892,2.656) | 6.577(2.831,10.915) | 0.736(0.312,1.229) | -1.964(-3.068,-0.846) |
| Portugal | 70.634(34.998,146.950) | 3.445(1.700,7.179) | 32.942(11.736,62.615) | 2.539(0.911,4.829) | -1.362(-2.503,-0.208) |
| Spain | 242.233(129.640,415.097) | 3.149(1.679,5.401) | 153.573(51.729,322.974) | 2.416(0.807,5.104) | -0.883(-1.972,0.218) |
| Sweden | 15.388(3.571,30.896) | 1.083(0.247,2.177) | 19.763(9.495,33.815) | 1.134(0.543,1.944) | 0.611(-0.308,1.538) |
| Switzerland | 23.193(11.394,42.538) | 2.149(1.054,3.943) | 14.445(5.767,30.656) | 1.144(0.456,2.431) | -2.063(-3.119,-0.996) |
| United Kingdom | 169.747(44.191,321.824) | 1.668(0.434,3.162) | 163.405(75.191,256.430) | 1.441(0.663,2.261) | -0.138(-1.238,0.975) |
| Argentina | 798.935(445.092,1292.399) | 8.409(4.683,13.607) | 639.788(370.175,1024.479) | 6.549(3.775,10.549) | -0.477(-1.333,0.386) |
| Chile | 258.518(113.219,438.224) | 6.998(3.059,11.864) | 158.284(82.956,246.446) | 4.522(2.361,7.065) | -0.856(-1.709,0.005) |
| Uruguay | 78.439(40.990,123.701) | 10.245(5.360,16.141) | 49.692(26.945,80.147) | 7.811(4.213,12.661) | -0.913(-1.820,0.003) |
| Canada | 131.973(71.800,217.080) | 2.426(1.319,3.991) | 94.421(40.965,173.797) | 1.565(0.680,2.882) | -1.750(-3.193,-0.286) |
| United States of America | 1636.335(1142.057,2271.022) | 3.146(2.196,4.365) | 1136.366(713.411,1565.137) | 1.981(1.237,2.731) | -0.856(-2.157,0.463) |
| Antigua and Barbuda | 0.813(0.422,1.301) | 4.743(2.461,7.592) | 0.711(0.401,1.157) | 4.474(2.523,7.309) | -0.156(-1.058,0.754) |
| Bahamas | 5.481(3.213,8.698) | 7.286(4.277,11.563) | 4.391(2.462,7.007) | 5.558(3.092,8.942) | -1.164(-1.592,-0.734) |
| Barbados | 10.992(6.560,16.582) | 18.561(11.044,27.966) | 5.024(2.921,7.784) | 10.953(6.261,17.078) | -0.992(-2.117,0.145) |
| Belize | 3.462(1.735,7.210) | 4.500(2.259,9.333) | 3.562(2.080,5.467) | 3.032(1.765,4.662) | -0.692(-0.902,-0.482) |
| Cuba | 358.523(171.957,612.738) | 15.477(7.441,26.418) | 89.677(42.406,169.207) | 5.272(2.482,9.984) | -1.031(-2.200,0.151) |
| Dominica | 1.283(0.417,3.005) | 5.559(1.809,13.032) | 0.793(0.274,1.843) | 6.090(2.074,14.351) | 0.750(0.030,1.475) |
| Dominican Republic | 100.120(28.517,269.450) | 3.987(1.141,10.707) | 136.913(43.758,356.480) | 4.998(1.597,13.021) | 0.764(0.096,1.436) |
| Grenada | 4.314(2.203,7.563) | 13.804(7.054,24.176) | 2.148(1.066,3.715) | 10.377(5.149,17.997) | -0.576(-1.292,0.145) |
| Guyana | 19.596(8.328,37.082) | 7.355(3.137,13.875) | 6.907(2.993,15.454) | 3.458(1.499,7.738) | -0.207(-0.766,0.355) |
| Haiti | 543.628(70.579,1988.212) | 20.812(2.816,75.222) | 684.523(149.515,2127.317) | 16.810(3.695,52.094) | -0.426(-0.743,-0.108) |
| Jamaica | 47.593(21.520,94.796) | 6.085(2.739,12.167) | 20.940(10.278,43.664) | 3.754(1.831,7.855) | -1.298(-1.922,-0.670) |
| Saint Lucia | 3.204(1.690,5.419) | 6.648(3.509,11.246) | 1.717(0.947,2.766) | 6.021(3.317,9.736) | -0.250(-0.874,0.378) |
| Saint Vincent and the Grenadines | 2.699(0.690,5.508) | 6.959(1.788,14.259) | 1.529(0.889,2.571) | 6.272(3.618,10.573) | -0.319(-0.641,0.003) |
| Suriname | 3.756(1.267,8.870) | 3.074(1.036,7.272) | 5.060(1.664,12.554) | 3.732(1.226,9.285) | 1.295(0.846,1.746) |
| Trinidad and Tobago | 34.099(19.274,56.130) | 8.762(4.961,14.399) | 15.192(8.556,24.242) | 5.781(3.232,9.243) | -0.984(-1.914,-0.047) |
| Bolivia (Plurinational State of) | 367.848(116.563,874.414) | 14.692(4.683,34.728) | 284.323(94.247,611.121) | 8.710(2.887,18.735) | -2.542(-3.245,-1.833) |
| Ecuador | 219.972(124.508,361.061) | 6.112(3.458,10.035) | 260.586(102.370,454.536) | 5.427(2.132,9.473) | -0.115(-1.011,0.788) |
| Peru | 881.247(369.695,1752.458) | 11.434(4.798,22.729) | 547.211(190.833,1146.502) | 6.132(2.138,12.849) | -2.135(-2.978,-1.284) |
| Colombia | 1005.966(582.517,1637.643) | 9.322(5.403,15.163) | 700.728(305.816,1369.778) | 7.043(3.074,13.775) | -0.162(-1.039,0.723) |
| Costa Rica | 79.689(36.562,131.329) | 7.583(3.497,12.480) | 58.109(19.919,120.636) | 5.990(2.039,12.441) | -0.931(-1.755,-0.099) |
| El Salvador | 85.915(41.705,154.309) | 4.314(2.096,7.742) | 50.323(22.814,86.905) | 2.919(1.319,5.049) | -1.420(-1.774,-1.065) |
| Guatemala | 314.228(163.192,653.906) | 8.223(4.287,17.033) | 190.740(104.863,287.131) | 4.071(2.234,6.140) | -1.782(-2.362,-1.199) |
| Honduras | 91.680(30.877,211.768) | 4.430(1.493,10.212) | 75.610(27.805,160.814) | 2.470(0.906,5.266) | -2.307(-2.562,-2.052) |
| Mexico | 1867.038(1094.544,3211.025) | 6.030(3.535,10.373) | 1330.219(683.686,1981.617) | 4.382(2.242,6.544) | -0.654(-1.242,-0.061) |
| Nicaragua | 125.062(52.320,239.052) | 7.257(3.035,13.861) | 80.348(40.420,141.288) | 4.312(2.167,7.588) | -1.770(-2.638,-0.895) |
| Panama | 75.921(45.978,119.522) | 9.750(5.904,15.355) | 98.730(58.191,145.218) | 9.053(5.329,13.332) | -0.323(-0.863,0.220) |
| Venezuela (Bolivarian Republic of) | 502.924(216.627,868.534) | 7.649(3.296,13.209) | 786.680(463.206,1188.165) | 12.758(7.495,19.305) | 1.254(0.367,2.149) |
| Brazil | 4730.454(3197.065,7180.378) | 9.703(6.528,14.774) | 3033.465(1587.317,4277.233) | 6.721(3.516,9.482) | -0.941(-1.697,-0.179) |
| Paraguay | 77.441(28.263,166.565) | 4.900(1.790,10.517) | 80.372(24.608,176.611) | 4.284(1.308,9.436) | -0.388(-1.037,0.264) |
| Algeria | 764.145(172.325,1902.926) | 7.527(1.704,18.731) | 639.613(187.780,1612.595) | 5.102(1.497,12.864) | -0.860(-1.959,0.251) |
| Bahrain | 2.876(0.772,7.131) | 1.914(0.517,4.742) | 2.436(0.553,6.131) | 0.852(0.193,2.144) | -2.799(-4.200,-1.378) |
| Egypt | 646.877(103.641,2315.236) | 3.206(0.513,11.420) | 427.805(40.050,1781.368) | 1.241(0.116,5.156) | -2.857(-3.897,-1.805) |
| Iran (Islamic Republic of) | 520.738(207.668,1348.183) | 2.124(0.849,5.480) | 304.544(124.544,582.666) | 1.531(0.623,2.933) | -0.794(-1.961,0.386) |
| Iraq | 833.314(120.909,2452.735) | 11.052(1.621,32.416) | 394.118(114.386,991.121) | 3.055(0.884,7.702) | -4.148(-5.044,-3.243) |
| Jordan | 95.834(31.164,231.013) | 6.372(2.072,15.351) | 141.174(51.736,314.557) | 4.045(1.484,9.042) | -2.304(-3.246,-1.352) |
| Kuwait | 27.243(13.892,53.014) | 5.283(2.702,10.252) | 13.425(6.139,26.133) | 1.641(0.749,3.192) | -3.193(-3.799,-2.584) |
| Lebanon | 52.988(16.197,125.822) | 5.554(1.701,13.150) | 37.128(12.023,88.255) | 3.007(0.968,7.166) | -2.170(-3.689,-0.626) |
| Libya | 117.323(31.553,309.445) | 6.977(1.876,18.404) | 124.836(34.607,318.739) | 8.938(2.375,23.089) | 1.411(0.226,2.609) |
| Morocco | 485.691(140.330,1310.951) | 5.370(1.558,14.466) | 306.943(104.558,714.373) | 3.319(1.127,7.747) | -1.224(-1.361,-1.087) |
| Palestine | 33.058(8.633,86.377) | 3.787(0.994,9.774) | 40.081(10.007,91.974) | 2.255(0.563,5.177) | -1.046(-3.341,1.304) |
| Oman | 32.119(9.130,81.642) | 4.191(1.203,10.648) | 36.241(11.022,82.315) | 3.127(0.949,7.108) | -0.590(-1.860,0.695) |
| Qatar | 1.453(0.296,3.941) | 1.317(0.270,3.571) | 3.819(0.947,9.357) | 0.844(0.210,2.061) | -1.253(-2.214,-0.282) |
| Saudi Arabia | 322.977(119.655,761.599) | 5.275(1.956,12.384) | 187.141(42.083,425.391) | 2.576(0.576,5.869) | -2.303(-3.205,-1.391) |
| Syrian Arab Republic | 78.588(26.421,191.581) | 1.426(0.481,3.471) | 60.002(14.960,133.948) | 1.746(0.434,3.916) | -0.091(-0.678,0.500) |
| Tunisia | 218.182(60.212,612.339) | 7.472(2.060,20.990) | 111.700(33.607,272.924) | 4.204(1.260,10.305) | -1.752(-2.801,-0.693) |
| T眉rkiye | 3470.687(1264.673,7372.341) | 18.093(6.583,38.458) | 1371.882(624.604,2431.556) | 7.580(3.432,13.498) | -2.969(-4.107,-1.817) |
| United Arab Emirates | 29.056(8.552,66.166) | 5.402(1.594,12.306) | 25.914(8.273,57.589) | 2.030(0.648,4.506) | -2.773(-3.437,-2.104) |
| Yemen | 303.783(47.427,1051.552) | 4.645(0.727,15.846) | 466.757(84.880,1293.083) | 3.598(0.655,9.964) | -0.785(-2.151,0.600) |
| Afghanistan | 366.985(59.452,1294.701) | 9.392(1.550,32.771) | 726.518(142.359,2102.149) | 5.619(1.096,16.240) | -1.195(-2.329,-0.047) |
| Bangladesh | 2037.424(427.681,6062.733) | 4.460(0.945,13.191) | 1566.565(389.129,3926.973) | 3.576(0.887,8.948) | -1.424(-2.140,-0.704) |
| Bhutan | 11.006(2.018,39.315) | 4.559(0.839,16.240) | 6.634(1.239,18.099) | 3.813(0.711,10.410) | -1.573(-2.556,-0.579) |
| India | 14843.329(4521.464,31010.227) | 4.802(1.467,10.032) | 7359.747(4488.656,11775.259) | 2.097(1.270,3.364) | -3.110(-3.792,-2.423) |
| Nepal | 282.769(69.181,878.584) | 3.634(0.905,11.187) | 259.549(52.700,686.186) | 3.018(0.613,7.975) | -1.379(-2.402,-0.346) |
| Pakistan | 5611.119(1845.574,12201.505) | 12.265(4.067,26.606) | 13620.840(4861.255,29268.916) | 17.008(6.068,36.569) | 1.215(1.026,1.405) |
| Angola | 2768.518(548.918,5860.169) | 60.338(12.666,126.702) | 3450.253(1330.177,6150.167) | 24.030(9.305,42.803) | -2.830(-4.230,-1.409) |
| Central African Republic | 563.449(144.110,1128.973) | 48.078(12.843,95.405) | 788.171(273.008,1473.007) | 36.936(12.897,68.880) | -0.681(-2.056,0.713) |
| Congo | 351.307(106.607,659.711) | 35.582(10.941,66.640) | 372.831(170.173,632.393) | 20.634(9.361,35.064) | -1.697(-2.964,-0.415) |
| Democratic Republic of the Congo | 7439.735(1775.923,14650.385) | 43.231(10.789,84.299) | 6743.109(2825.785,12112.357) | 18.965(7.971,34.014) | -2.103(-3.361,-0.829) |
| Equatorial Guinea | 84.207(22.777,166.792) | 44.632(12.615,87.315) | 121.159(44.936,288.923) | 21.965(8.091,52.227) | -2.928(-4.109,-1.733) |
| Gabon | 108.228(39.031,192.906) | 28.367(10.378,50.332) | 137.756(60.449,246.780) | 22.990(10.044,41.271) | -0.339(-1.662,1.003) |
| Burundi | 2923.006(987.001,5889.838) | 117.924(40.333,235.952) | 2628.999(1159.618,5123.951) | 48.453(21.428,94.034) | -2.644(-3.285,-2.000) |
| Comoros | 162.888(61.620,305.987) | 82.714(31.416,154.588) | 130.263(63.671,233.164) | 58.032(28.301,104.006) | -1.462(-1.920,-1.003) |
| Djibouti | 102.811(40.745,187.101) | 64.216(25.642,116.330) | 206.268(94.880,374.387) | 53.463(24.606,96.991) | -0.604(-1.197,-0.007) |
| Eritrea | 1188.830(434.138,2174.007) | 79.695(29.392,145.139) | 1476.905(682.064,2765.140) | 62.575(28.971,116.945) | -0.894(-1.497,-0.287) |
| Ethiopia | 20952.913(5656.356,43275.031) | 92.015(25.086,189.216) | 17790.414(8730.917,29618.205) | 43.349(21.311,72.029) | -2.704(-3.799,-1.598) |
| Kenya | 2634.156(1183.698,4022.104) | 25.239(11.456,38.277) | 3563.478(2075.395,5025.182) | 20.199(11.727,28.604) | 0.380(-0.648,1.419) |
| Madagascar | 3699.482(1516.972,6531.183) | 72.894(30.235,127.743) | 4894.777(2510.588,8345.703) | 44.520(22.818,75.941) | -1.122(-1.705,-0.535) |
| Malawi | 8209.824(2926.332,15197.587) | 191.757(69.182,351.201) | 8794.650(3567.530,17714.167) | 116.320(47.117,235.181) | -1.419(-1.959,-0.877) |
| Mauritius | 2.751(1.738,4.144) | 0.901(0.569,1.354) | 1.521(0.775,2.530) | 0.785(0.396,1.301) | -0.712(-1.390,-0.030) |
| Mozambique | 936.448(310.915,2240.400) | 15.981(5.354,38.238) | 980.661(353.492,2603.461) | 7.368(2.654,19.503) | -2.164(-2.957,-1.365) |
| Rwanda | 4029.546(1385.383,7467.331) | 126.393(43.879,233.036) | 2555.836(1267.971,4631.932) | 55.179(27.404,99.962) | -3.293(-3.801,-2.782) |
| Seychelles | 0.000(0.000,0.002) | 0.002(0.000,0.009) | 0.001(0.000,0.002) | 0.003(0.000,0.011) | 0.840(-0.133,1.822) |
| Somalia | 2552.419(812.756,5272.217) | 69.628(22.458,142.681) | 4735.858(1730.871,9084.568) | 49.958(18.413,95.189) | -1.101(-1.906,-0.291) |
| United Republic of Tanzania | 11055.094(4126.263,19680.170) | 98.426(37.092,173.966) | 14389.594(6856.677,25642.894) | 63.228(30.205,112.386) | -0.987(-1.652,-0.317) |
| Uganda | 10061.726(4924.055,17043.137) | 125.304(62.596,210.277) | 22114.486(11114.123,39632.911) | 119.784(60.435,213.835) | -0.146(-0.789,0.500) |
| Zambia | 3408.464(1241.949,6102.440) | 96.680(35.878,171.582) | 4312.202(1958.319,8093.486) | 55.660(25.256,104.520) | -1.939(-2.691,-1.182) |
| Botswana | 35.104(11.596,76.594) | 6.391(2.115,13.923) | 81.612(24.992,173.484) | 12.581(3.846,26.799) | 2.697(1.662,3.742) |
| Lesotho | 36.451(12.740,77.988) | 5.730(2.004,12.259) | 64.688(21.586,142.554) | 10.858(3.622,23.944) | 2.773(1.540,4.020) |
| Namibia | 57.667(19.470,135.264) | 10.388(3.536,24.261) | 126.413(47.752,271.900) | 16.404(6.194,35.350) | 2.166(1.082,3.261) |
| South Africa | 405.123(177.430,752.395) | 3.190(1.402,5.912) | 560.007(344.782,889.061) | 3.910(2.402,6.208) | 0.402(-0.295,1.104) |
| Eswatini | 30.149(9.075,72.311) | 8.372(2.534,19.957) | 45.161(13.800,101.098) | 11.704(3.577,26.192) | 1.567(0.274,2.878) |
| Zimbabwe | 434.358(145.899,986.200) | 9.720(3.272,22.031) | 1433.588(439.190,3211.456) | 24.379(7.467,54.621) | 5.157(3.430,6.913) |
| Benin | 1319.551(429.321,2536.624) | 56.539(18.870,107.544) | 3091.188(1390.286,5562.108) | 54.030(24.468,96.636) | 0.003(-1.095,1.112) |
| Burkina Faso | 2728.071(937.537,5229.274) | 60.461(21.163,115.104) | 5432.068(2364.110,9801.598) | 55.933(24.728,100.200) | 0.218(-0.830,1.277) |
| Cameroon | 2653.037(976.727,4983.946) | 57.525(21.746,107.022) | 7552.045(3384.374,12994.860) | 59.934(26.918,103.075) | 0.547(-0.310,1.411) |
| Cabo Verde | 15.463(6.576,29.168) | 10.527(4.484,19.801) | 38.240(13.413,68.753) | 27.688(9.775,49.738) | 1.736(0.885,2.595) |
| Chad | 1312.783(433.429,2559.027) | 47.312(16.136,91.008) | 4670.768(1883.934,8170.600) | 55.463(22.671,96.630) | 0.894(-0.256,2.057) |
| C么te d'Ivoire | 3048.302(1227.939,5595.039) | 56.669(23.229,103.013) | 5507.641(2419.254,10082.851) | 51.119(22.516,93.172) | 0.158(-0.394,0.714) |
| Gambia | 220.110(84.472,427.790) | 50.490(19.882,97.055) | 400.722(167.363,769.390) | 43.387(18.189,83.110) | -0.393(-1.291,0.512) |
| Ghana | 6205.792(1668.559,12717.109) | 98.569(26.583,201.136) | 4256.571(2183.062,7818.774) | 35.375(18.142,64.849) | -4.201(-5.381,-3.007) |
| Guinea | 1628.715(546.991,3305.355) | 60.993(21.475,121.802) | 2291.547(991.363,4567.386) | 40.381(17.604,80.114) | -0.724(-1.328,-0.117) |
| Guinea-Bissau | 322.780(111.844,641.370) | 71.137(25.074,140.406) | 367.496(169.960,653.828) | 44.138(20.449,78.558) | -1.085(-2.126,-0.033) |
| Liberia | 848.887(263.106,1700.573) | 77.668(24.842,153.387) | 988.929(426.034,1720.445) | 48.599(20.948,84.513) | -1.532(-2.350,-0.707) |
| Mali | 1783.547(691.449,3374.328) | 44.733(17.908,83.511) | 3171.204(1456.855,5864.070) | 29.237(13.626,53.379) | -0.977(-1.825,-0.120) |
| Mauritania | 350.717(144.101,650.858) | 40.619(16.966,74.814) | 568.449(276.443,993.349) | 32.729(15.927,57.162) | -0.887(-1.607,-0.162) |
| Niger | 3011.462(854.366,6437.979) | 77.001(22.519,162.583) | 5078.658(2033.107,9706.784) | 42.314(17.147,80.171) | -1.820(-2.906,-0.721) |
| Nigeria | 24699.947(10362.883,42458.792) | 66.509(28.784,113.188) | 52225.414(24367.626,78765.077) | 54.984(25.826,82.690) | -0.405(-1.330,0.529) |
| Sao Tome and Principe | 34.151(12.808,64.084) | 64.397(24.164,120.737) | 17.884(8.342,32.276) | 24.293(11.265,44.031) | -2.563(-3.295,-1.825) |
| Senegal | 2093.751(764.244,3895.164) | 60.063(22.459,110.900) | 2171.594(1095.517,3801.258) | 36.744(18.561,64.242) | -1.345(-2.200,-0.484) |
| Sierra Leone | 1290.346(394.593,2654.738) | 73.636(23.431,148.634) | 1835.252(847.949,3270.867) | 55.263(25.717,98.131) | -0.680(-1.639,0.288) |
| Togo | 826.718(319.665,1475.777) | 49.569(19.400,88.139) | 1255.950(615.255,2290.232) | 40.482(19.827,73.738) | -0.295(-1.246,0.665) |
| American Samoa | 0.109(0.020,0.308) | 0.611(0.112,1.760) | 0.198(0.046,0.541) | 1.506(0.362,4.068) | 3.125(1.893,4.371) |
| Bermuda | 0.920(0.332,1.726) | 8.210(2.951,15.390) | 0.415(0.141,0.826) | 5.317(1.787,10.648) | -1.088(-1.338,-0.839) |
| Cook Islands | 0.051(0.012,0.135) | 0.835(0.195,2.205) | 0.035(0.004,0.109) | 1.044(0.111,3.202) | -0.329(-0.579,-0.079) |
| Greenland | 1.133(0.140,2.890) | 8.717(1.088,22.220) | 0.187(0.046,0.508) | 1.703(0.420,4.628) | -4.049(-5.268,-2.814) |
| Guam | 0.585(0.185,1.385) | 1.463(0.459,3.459) | 1.159(0.565,1.943) | 3.395(1.650,5.696) | 5.009(4.467,5.554) |
| Monaco | 0.023(0.004,0.069) | 0.697(0.110,2.104) | 0.039(0.008,0.106) | 0.811(0.164,2.233) | 0.226(-0.306,0.761) |
| Nauru | 0.082(0.022,0.224) | 1.987(0.526,5.470) | 0.148(0.035,0.420) | 3.978(0.927,11.261) | 2.259(2.042,2.478) |
| Niue | 0.012(0.003,0.036) | 1.733(0.404,5.137) | 0.015(0.003,0.056) | 4.586(0.888,16.497) | 2.541(1.763,3.324) |
| Northern Mariana Islands | 0.057(0.008,0.185) | 0.496(0.074,1.605) | 0.097(0.023,0.265) | 0.967(0.245,2.568) | 3.778(3.212,4.347) |
| Palau | 0.072(0.019,0.181) | 1.760(0.477,4.435) | 0.053(0.016,0.118) | 1.930(0.609,4.293) | 0.677(-0.737,2.111) |
| Puerto Rico | 84.483(35.418,143.932) | 9.066(3.789,15.470) | 19.038(8.129,30.365) | 4.373(1.853,7.056) | -1.793(-2.565,-1.014) |
| Saint Kitts and Nevis | 0.475(0.238,0.867) | 3.568(1.786,6.542) | 0.323(0.161,0.666) | 3.426(1.691,7.071) | 0.664(-0.078,1.412) |
| San Marino | 0.160(0.037,0.417) | 4.157(0.952,10.873) | 0.141(0.031,0.332) | 3.248(0.703,7.705) | -0.626(-2.466,1.249) |
| Tokelau | 0.007(0.002,0.021) | 1.309(0.320,3.898) | 0.010(0.002,0.034) | 3.103(0.568,10.798) | 1.340(0.793,1.890) |
| Tuvalu | 0.089(0.018,0.281) | 2.465(0.506,7.767) | 0.076(0.021,0.201) | 2.179(0.600,5.773) | -0.059(-0.202,0.084) |
| United States Virgin Islands | 0.903(0.243,2.180) | 3.012(0.807,7.290) | 0.201(0.043,0.519) | 1.565(0.334,4.060) | -0.937(-1.632,-0.238) |
| South Sudan | 2109.191(777.543,3975.436) | 85.306(31.727,159.943) | 4109.276(1663.723,7681.764) | 104.623(42.482,195.197) | 0.643(0.000,1.291) |
| Sudan | 566.277(86.350,2432.282) | 6.897(1.063,29.319) | 797.936(150.717,2160.730) | 5.120(0.967,13.870) | -0.931(-1.914,0.063) |
